# Identification of novel dysregulated key genes in Breast cancer through high throughput ChIP-Seq data analysis

**DOI:** 10.1038/s41598-017-03534-x

**Published:** 2017-06-12

**Authors:** Utkarsh Raj, Imlimaong Aier, Rahul Semwal, Pritish Kumar Varadwaj

**Affiliations:** Department of Bioinformatics & Applied Sciences, Indian Institute of Information Technology, Allahabad, Uttar Pradesh India

**Keywords:** Computational biology and bioinformatics, Genome informatics

## Abstract

Breast cancer is the most common cancer in women both in the developed and less developed countries, and it imposes a considerable threat to human health. Therefore, in order to develop effective targeted therapies against Breast cancer, a deep understanding of its underlying molecular mechanisms is required. The application of deep transcriptional sequencing has been found to be reported to provide an efficient genomic assay to delve into the insights of the diseases and may prove to be useful in the study of Breast cancer. In this study, ChIP-Seq data for normal samples and Breast cancer were compared, and differential peaks identified, based upon fold enrichment (with P-values obtained via t-tests). The Protein–protein interaction (PPI) network analysis was carried out, following which the highly connected genes were screened and studied, and the most promising ones were selected. Biological pathway involved in the process were then identified. Our findings regarding potential Breast cancer-related genes enhances the understanding of the disease and provides prognostic information in addition to standard tumor prognostic factors for future research.

## Introduction

Breast cancer is the topmost cause of cancer in women as reported in both developed and developing nations^1^. Increase in life expectancy, urbanization and adoption of western lifestyle has played a prominent role in the significant rise of breast cancer incidents in developing countries^2^. The worldwide threat to this cancer is expected go beyond two million by the year 2030, with growing proportions from developing nations^3^. Although age- standardized incidence rates in India are lower than in the United Kingdom (UK) (25.8 versus 95 per 100,000), mortality rates are nearly as high (12.7 versus 17.1 per 100,000, respectively) as those of the UK^1^. The symptoms may comprise of a lump in the breast, a change in breast shape, dimpling of the skin, fluid coming from the nipple, or a red scaly patch of skin. In those with distant spread of the disease, there may be bone pain, swollen lymph nodes, shortness of breath, or yellow skin^4^. However, some reduction in the risk of its occurrence might be possible with prevention, these measure cannot eradicate the major proportion of breast cancers that develop in low- as well as middle- income nations where breast cancer is diagnosed in very late stages^5, 6^. Control of breast cancer, therefore, lies in early detection. Breast cancer is a heterogeneous disease exhibiting high tumor variability in terms of the underlying biological mechanisms, response to treatment, and overall survival rate^7^. Genetic background contributes to the risk of the Breast cancer, as suggested by associations with race, family and specific gene variants^8^. Many genes have been reported to be involved in Breast cancer, for instance, most of the inherited cases of this disease are associated with two abnormal genes: *BRCA1* (BReast CAncer gene one) and *BRCA2* (BReast CAncer gene two)^9^. *BRCA1* and *BRCA2* are two breast cancer susceptibility genes. However, the expression of these two genes vary greatly depending on the cell lines. For instance, mutation of the genes can result in their overexpression. These mutations, however, are not seen in the MCF7 or MDA-MB-231 cell lines selected for this study. However, *BRCA1* and *BRCA2* mutations are very rare and only accounts for around 5% of all breast cancers. Moreover, most breast cancer cases are thought to be sporadic.

Deciding the collaboration of proteins with DNA is critical in understanding the state of diseases and it’s relation to biological pathways. Information thus obtained in this manner can prompt a more profound comprehension of tumor development. Therefore, to investigate interactions between chromatin-associated protein and DNA, a Chromatin immunoprecipitation sequencing (ChIP-Seq) is utilized^10^. ChIP-Seq helps in the identification of the binding sites of any DNA-associated proteins^10^.

Recently, ChIP-Seq analysis has been carried out to study Breast cancer. The study reports that the molecular signature at histone H3K4me3 and H3K27me3 are involved in the epigenetic control of normal (MCF10A) and transformed (MCF7, MDA-MB-231) breast cells using ChIP-Seq technology^11, 12^. MCF7 belongs to the class estrogen receptor-positive breast cancer cell lines, while MDA-MB-231 belongs to the class estrogen receptor-negative breast cancer cell line, also known as triple negative breast cancer, where the receptors for estrogen, progesterone and HER2 are all negative (ER-, PR- and HER-). Such studies provide meaningful insights for understanding the molecular mechanisms and the dynamic distribution of H3K4me3, associated with active chromatin, and H3K27me3, associated with repressed chromatin, histone modifications to provide an understanding of the changes in epigenetic regulation associated with the unique breast cancer subtypes from ChIP-Seq data^12^. Very few studies using ChIP-Seq data for Breast cancer-related gene identification has been carried out till date^13, 14^.

**Figure 1:**
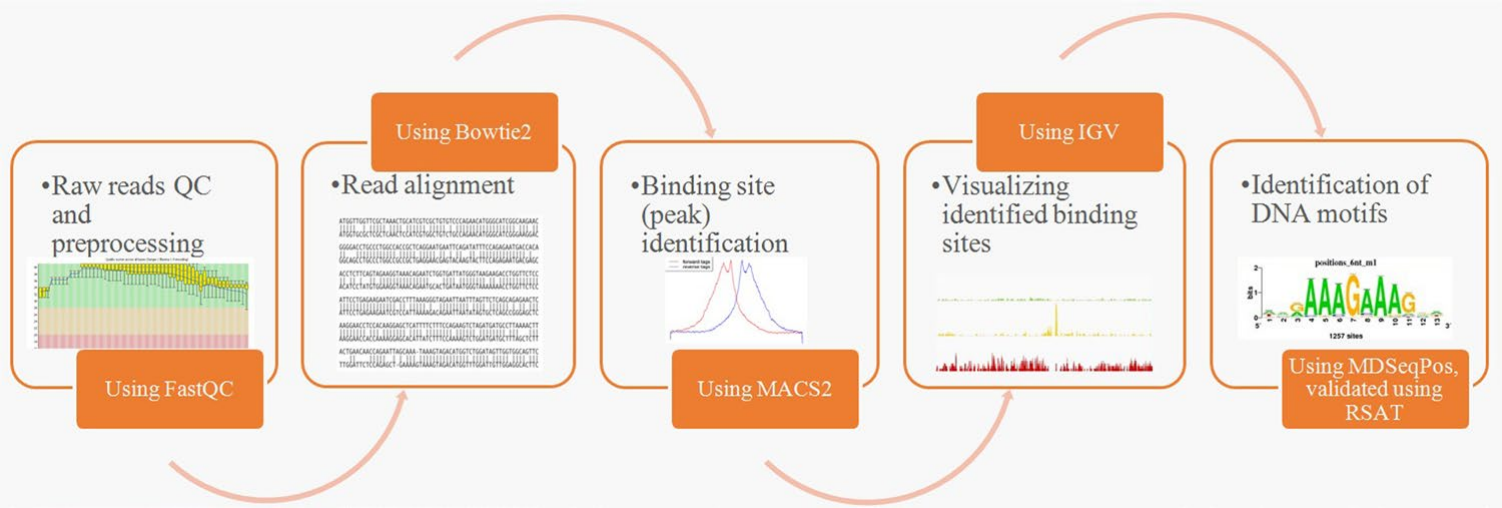
ChIP-Seq analysis workflow used in this study. Diagrammatic representation of the steps performed for Chip-Seq data analysis used in this study.

The study of protein–protein interaction (PPI) networks and biological pathways provide important information required for understanding the various cellular functions and biological processes. With the gigantic increment in the data of human protein interaction, this approach can be utilized to comprehend underlying molecular mechanism of the disease, especially to analyze the phenomena related to cancer^15, 16^. It also provides insights into distinct topological features of genes related to cancer^17^. Therefore, ChIP-Seq data derived from normal and cancerous breast samples were analyzed in this study, and differential peaks were screened out. Enriched peaks were annotated and PPI network analysis and pathway analysis of novel genes were carried out for the identification of critical genes related to the Breast cancer. The basic steps involved in ChIP-Seq analysis is represented in the form of flowchart in Fig. 1.

## Results and Discussion

### Quality Analysis of reads for normal and cancerous tissue samples

**Figure 2:**
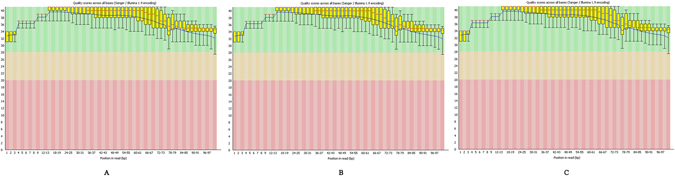
Box plot generated by FastQC. Quality score for A) MCF10A B) MCF7 C) MDA-MB-231.

The results obtained from FastQC gave a basic idea about the quality of the data. Results obtained from quality check were shown in Fig. 2.

A basic statistics report gave information about the experimental genome like number of bases, the number of bases per read and number of reads etc. Fig. 2 shows the quality score values across all bases in the genome for the cancerous cell lines MCF-7 & MDA-MD-231 as well as for the normal cell line MCF-10A. For each position, a Box-Whisker plot is drawn. For the data under study, the quality scores for the bases in all reads do not fall below 22 which were good. The plot consists of: the central red line is the median value; the yellow box represents the inter-quartile range (25–75%); the upper and lower whiskers represent the 10% and 90% points and the blue line represents the mean quality. As evident from the Fig. 2, the y-axis on the graph shows the quality scores. The higher the score the better the base call. The background of the graph divides the y-axis into very good quality calls (green), calls of reasonable quality (orange), and calls of poor quality (red). The quality of calls on most platforms will degrade as the run progresses, so it is common to see base calls falling into the orange area towards the end of a read. In this case, the quality score in all the cell lines MCF-7, MDA-MB-231 & MCF-10A qualifies all the criteria and all the values lie in the acceptable range.

### Comparison between peak regions of MCF10A, MCF7 and MDA-MB-231

**Figure 3:**
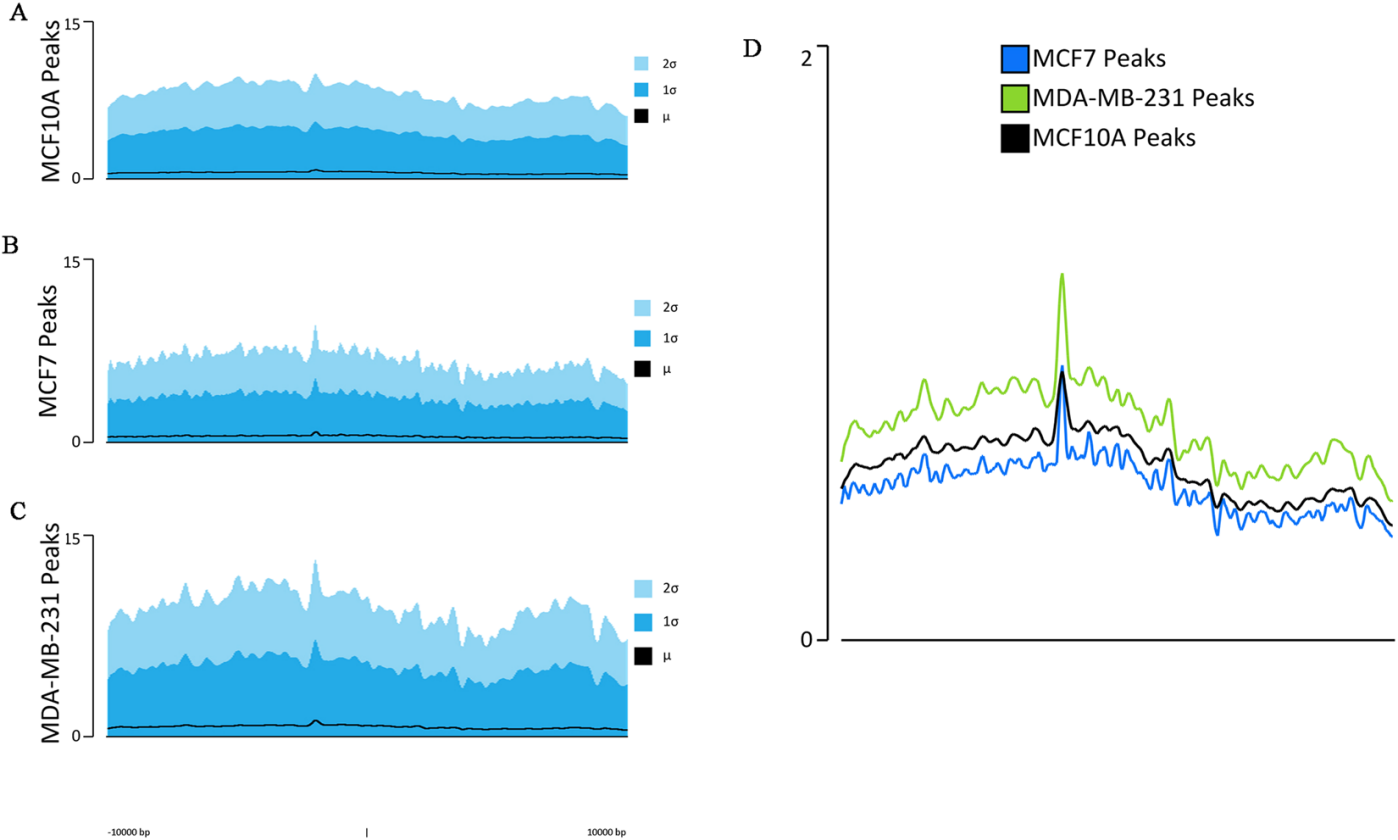
Comparison of average peak signal intensity of MCF10A, MCF7 and MDA-MB-231. (**A**) Average signal (μ) and standard deviation (1σ, 2σ) for MCF10A with intensity at y-axis for 2006 regions. 20000 bp surrounding each region, segmented into 400 bins were represented on the x-axis. (**B**) Average signal and standard deviation for MCF7 with intensity at y-axis for 2589 regions. 20000 bp surrounding each region, segmented into 400 bins were represented on the x-axis. (**C**) Average signal and standard deviation for MDA-MB-231 with intensity at y-axis for 2945 regions. 20000 bp surrounding each region, segmented into 400 bins were represented on the x-axis. (**D**) The average overlay peaks for all three samples.

A total of 2,589 enriched peaks were observed in the MCF7 sample, while the MDA- MB-231 sample exhibited a total of 2,945 enriched peaks. The normal sample displayed a total of 2,006 enriched peaks for the differential analysis in both the cancer types. It was observed that the peaks were significantly higher in both the cancer type as compared to peaks in the normal sample (Fig. 3).

**Figure 4:**
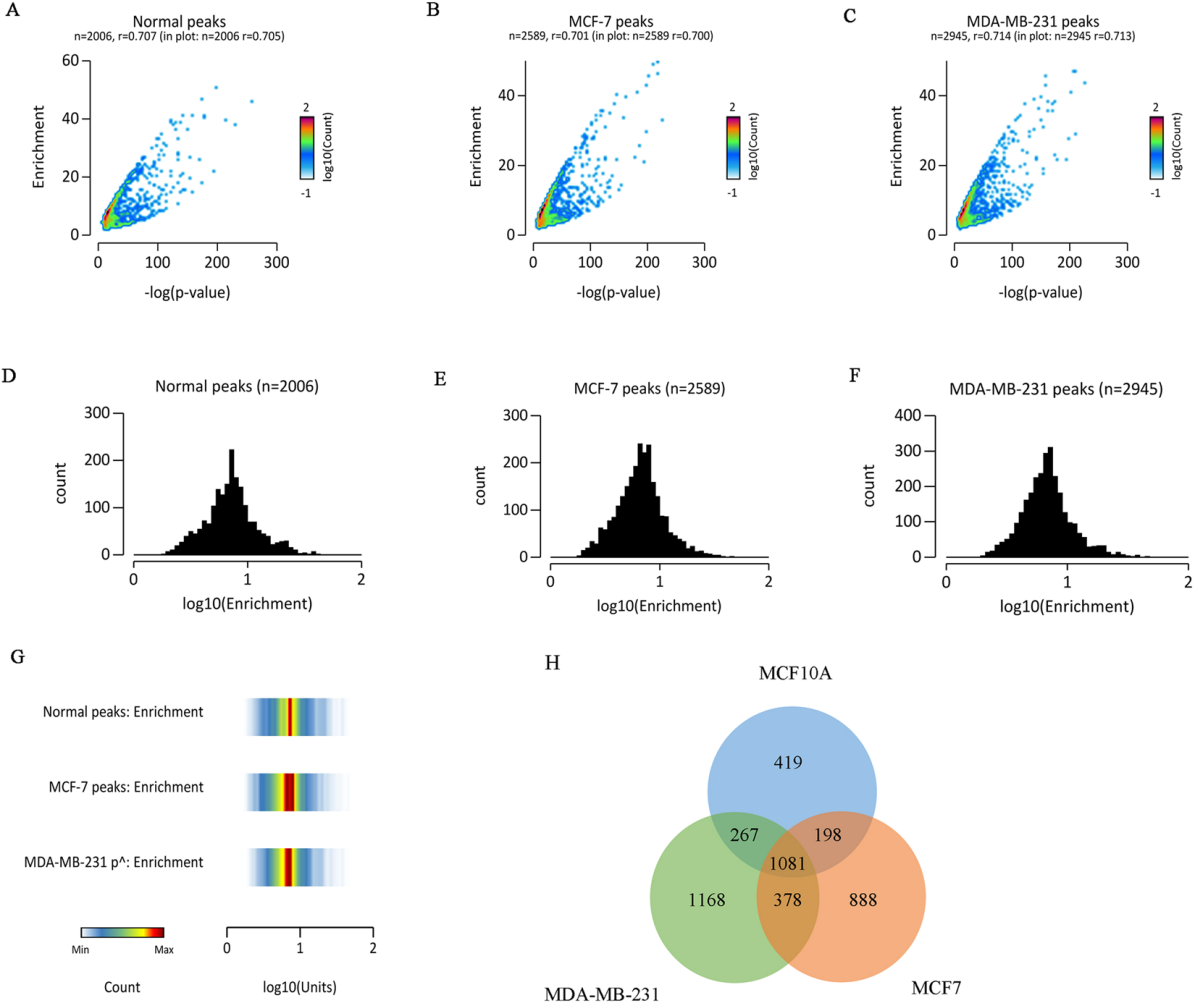
Comparison of enriched regions between MCF10A, MCF7 and MDA-MB-231. (**A B** and **C**) are 2D histograms of the –log (p-value) on the x-axis and the enrichment value (FE) on the y-axis for MCF10A (Normal), MCF7 and MDA-MB-231 respectively. Each region on the x and y axis were segmented into 75 and 75 bins. The number of regions within each bin were counted. (**D**,**E** and **F**) shows the histogram of the enrichment parameter found in MCF10A (Normal), MCF7 and MDA-MB-231 peaks respectively. The values on the x-axis were segmented into 50 bins and presented on a log scale with a base of 10. (**G**) Density plot of the distribution selected parameters for MCF10A (Normal), MCF7 and MDA-MB-231 respectively. The y-axis was segmented into 49 bins and the number of regions within each bin was represented by a color code. The color coding is shown in the bottom corner of the plot. (**H**) Venn diagram of common locations shared between MCF10A, MCF7 and MDA-MB-231.

The enrichment plots obtained from EaSeq displayed differences in the overall distribution of expressed regions in Fig. 4A, B and C. The R value or the correlation coefficient, which indicates the linear relationship between two variables on a scatter plot, were found to be 0.705, 0.700 and 0.713 for normal, MCF7 and MDA-MB-231 respectively. The histogram for the datasets, as given in Fig. 4D, E and F, indicated that the enrichment peak count for the normal sample lies around 230, while the count for MCF7 and MDA-MB-231 lies around 250 and 310 respectively, indicating that the two cancer samples are more enriched than the normal type. Density plot of normal peaks were visualized as a thin orange band, while MCF7 and MDA-MB-231 peaks were more prominent and broad; MCF7 peaks being the broadest (Fig. 4G). A Venn diagram was constructed to find out the common regions between the three datasets. It was found that MCF10A contained 419 locations, while MCF7 and MDA-MB-231 each contained 888 and 1168 locations respectively. The common regions between MCF10A and MCF7 was found to be 198, while common regions between MCF10A and MDA-MB-231 was found to be 267. MCF7 and MDA-MB-231 shared a common region of 378 and the region common to all three datasets were 1081 in total (Fig. 4H).

### Peak enrichment annotation and motif analysis

**Figure 5:**
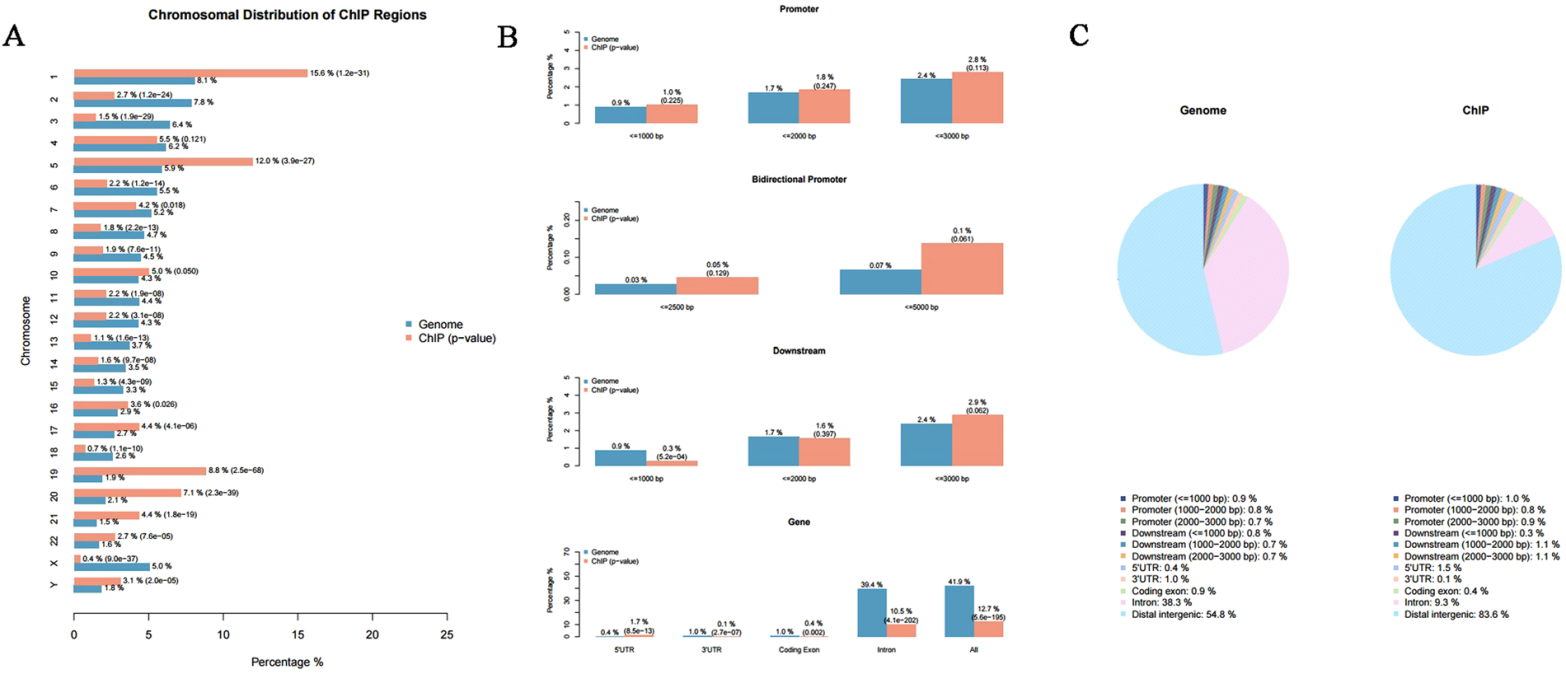
Distribution of ChIP regions over chromosomes for MCF7. (**A**) The blue bars represent the percentages of the whole tiled or mappable regions in the chromosomes (genome background) and the red bars the percentages of the whole ChIP. These percentages are also marked right next to the bars. P-values for the significance of the relative enrichment of ChIP regions with respect to the gnome background are shown in parentheses next to the percentages of the red bars. (**B**) Relative enrichments of ChIP regions in important genomic features, such as promoters, immediate downstream of genes, and gene bodies, with respect to the genome background. (**C**) Pie chart showing how ChIP regions are distributed over important genomic features.

The peak region of MCF7 was annotated using EaSeq (Supplementary Table S1) and the functional regions present across the entire genome were examined using the CEAS package present in Cistrome. From Fig. 5A, it is clear that 15.6% of ChIP regions were present on chr1, while 8.1% of the whole tiled (or mappable regions) occupy chr1 with a P-value of 1.2e-31. A sum of 100% was calculated for the red bars (or blue bars, equivalently). The mappable regions present in the genome were represented by blue bars while the percentage of ChIP regions were shown in red bars. Relatively high enrichments, 12.7% of ChIP regions, were found in gene bodies for H3K27me3 compared to 41.9% of the genome background (Fig. 5B lower panel). However, promoter regions displayed very low enrichment, including bidirectional ones (only 2.8% of ChIP regions within 3 kb upstream of TSS), due to the fact that H3K27me3 is a transcriptional elongation (Fig. 5B upper panel) mark. In addition, considering the high enrichment in H3K27me3’s immediate downstream (Fig. 5B), H3K27me3 was observed to contain a long tail after Transcription terminating site (TTS). Figure 4C was used to represent the distribution of promoter regions, downstream regions, 5′ UTR and 3′ UTR, coding exons, introns and distal intergenic regions in the form of a pie chart.

**Figure 6:**
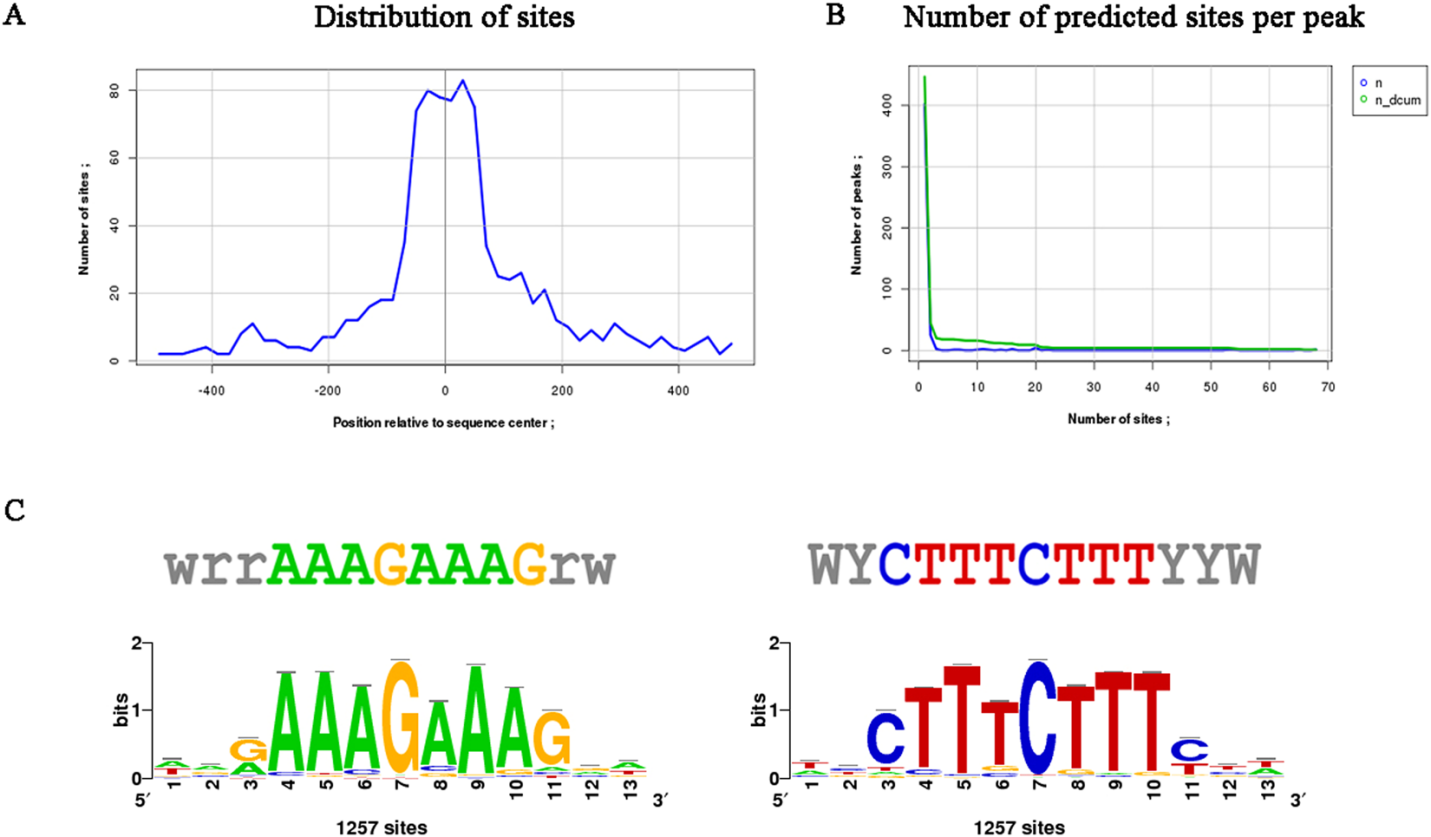
Motif analysis and distribution of sites for MCF7 obtained from RSAT. (**A**) Distribution of sites shows the position relative to the sequence center vs. the number of sites on the x and y axis respectively. (**B**) The number of sites predicted per peak is represented by a graph between the number of sites on the x-axis and the number of peaks on the y-axis. n and n_dcum stands for occurrences and decreasing cumulative occurrences (inclusive) respectively. (**C**) Forward and reverse sequence logo for *LEF1* obtained using RSAT.

The peak regions of MCF7 were scanned for motifs using MDSeqPos, available in Cistrome. Motifs with lower Z-score are generally considered more significant. An MDSeqPos Z-score cutoff of < = −15 was used for this study. Motif identification was further validated using the RSAT tool available online. Regulatory Sequence Analysis Tools (RSAT) is an online modular genome sequence analysis suite for the discovery of cis-regulatory elements. The programs available on RSAT can be accessed individually or collectively through interconnected pipelines. The main purpose of RSAT is to identify regulatory signals and locate enriched motifs in a set of sequence. One of the top motif identified by both MDSeqPos and RSAT for MCF7 was *LEF1* (Fig. 6), which possessed a Z-score of −9.275 and a significance index (−log10 (e-value)) of 10.21. The significance index represents the log-transformation of the e-value.

**Figure 7:**
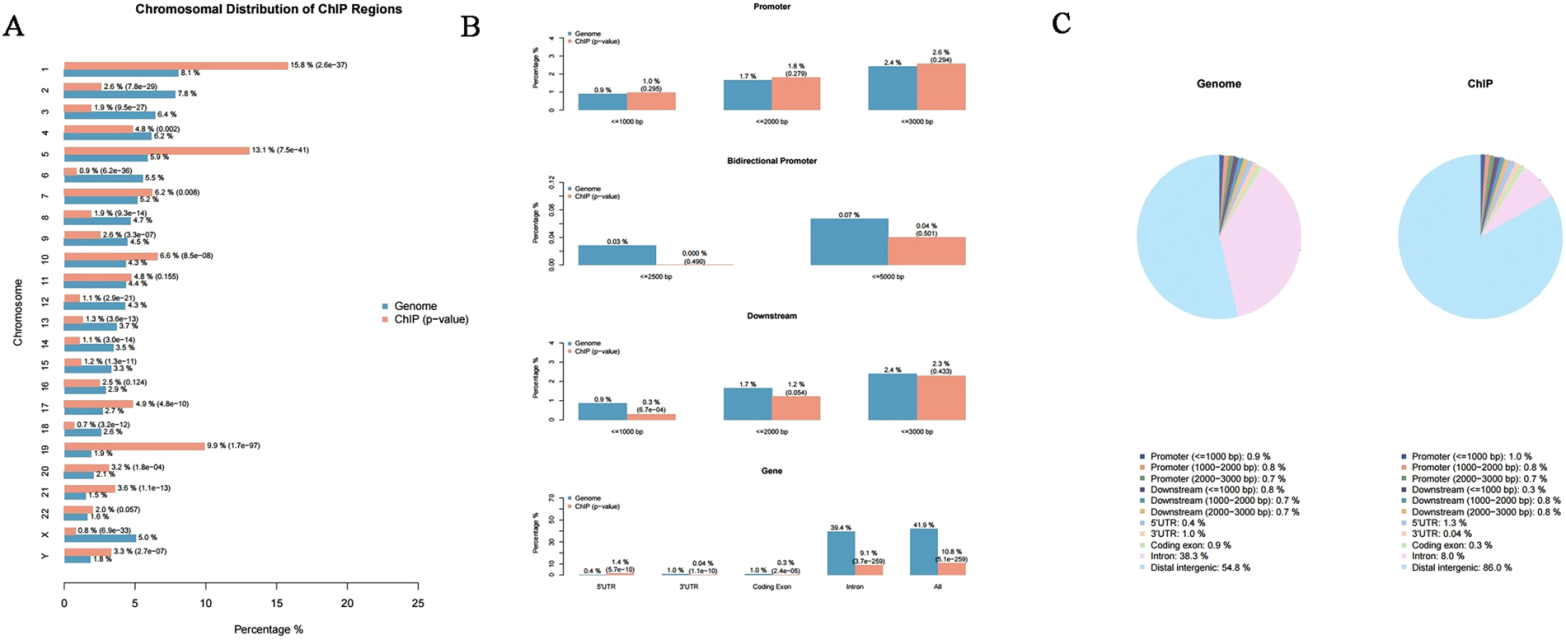
Distribution of ChIP regions over chromosomes for MDA-MB-231. (**A**) The blue bars represent the percentages of the whole tiled or mappable regions in the chromosomes (genome background) and the red bars the percentages of the whole ChIP. These percentages are also marked right next to the bars. P-values for the significance of the relative enrichment of ChIP regions with respect to the gnome background are shown in parentheses next to the percentages of the red bars. (**B**) Relative enrichments of ChIP regions in important genomic features, such as promoters, immediate downstream of genes, and gene bodies, with respect to the genome background. (**C**) Pie chart showing how ChIP regions are distributed over important genomic features.

Figure 7 shows the ChIP region annotation (Supplementary Table S2) & average profiling within/near important genomic features for MDA-MB-231. From the Fig. 7A, it is clear that 15.8% of ChIP regions were present on chr1, while 8.1% of the whole tiled (or mappable regions) occupy chr1 with a P-value of 2.6e-37. A sum of 100% was calculated for the red bars (or blue bars, equivalently). The mappable regions present in the genome were represented by blue bars while the percentage of ChIP regions were shown in red bars (Fig. 7A). Relatively high enrichments, 10.8% of ChIP regions, were found in gene bodies for H3K27me3 compared to 41.9% of the genome background. However, promoter regions displayed very low enrichment, including bidirectional ones (only 2.6% of ChIP regions within 3 kb upstream of TSS), due to the fact that H3K27me3 is a transcriptional elongation mark. In addition, considering the high enrichment in H3K27me3’s immediate downstream (Fig. 7B), H3K27me3 was observed to contain a relatively shorter tail after Transcription terminating site (TTS).

**Figure 8:**
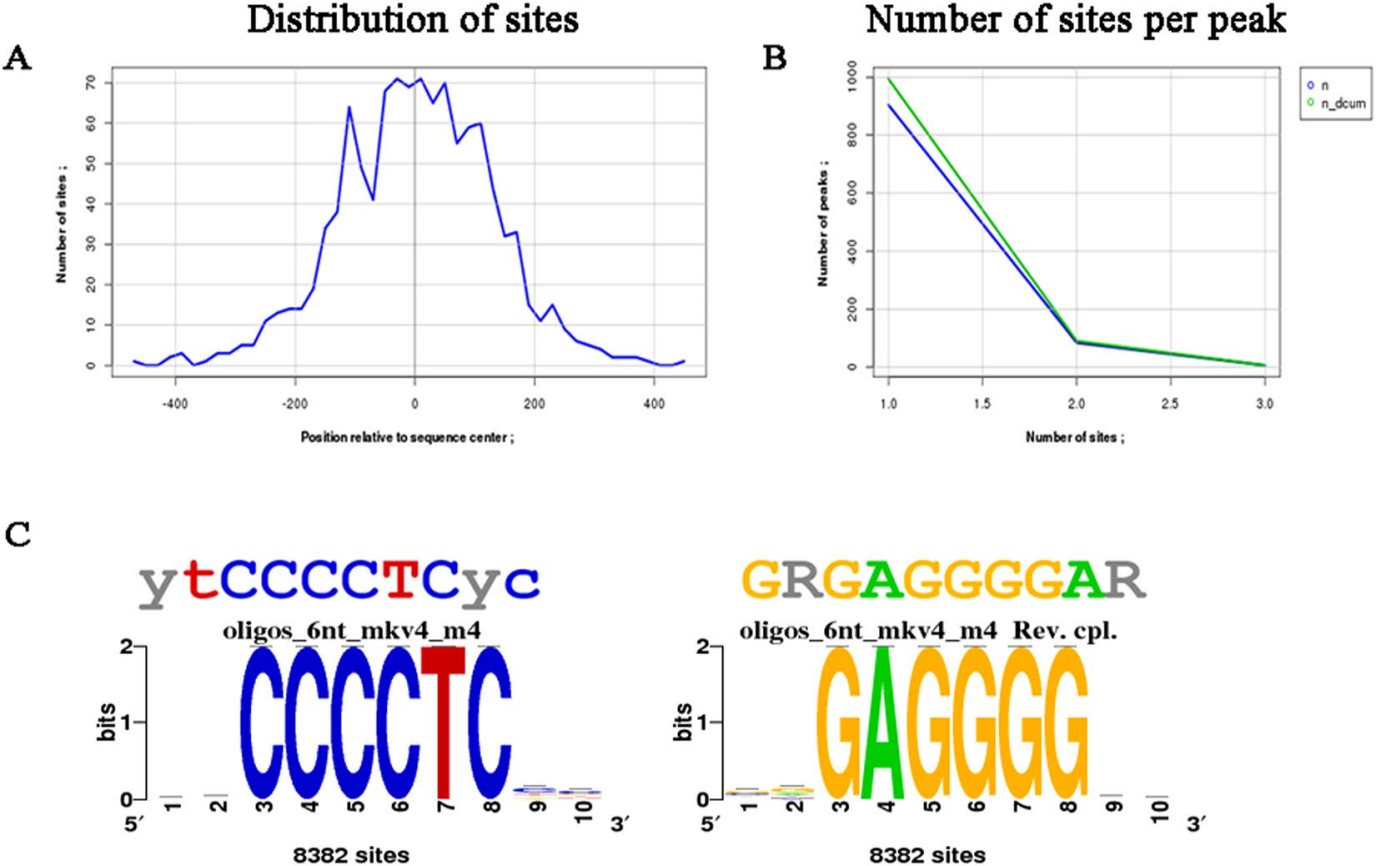
Motif analysis and distribution of sites for MDA-MB-231 obtained from RSAT. (**A**) Distribution of sites shows the position relative to the sequence center vs. the number of sites on the x and y axis respectively. (**B**) The number of sites predicted per peak is represented by a graph between the number of sites on the x axis and the number of peaks on the y axis. n and n_dcum stands for occurrences and decreasing cumulative occurrences (inclusive) respectively. (**C**) Forward and reverse sequence logo for MZF1 obtained using RSAT.

One of the top motif identified by both MDSeqPos and RSAT for MDA-MB-231 was *MZF1* (Fig. 8), which possessed a Z-score of −11.565 and a significance index (−log10 (e-value)) of 21.47.

### Protein-protein interaction network of peak associated genes

**Figure 9:**
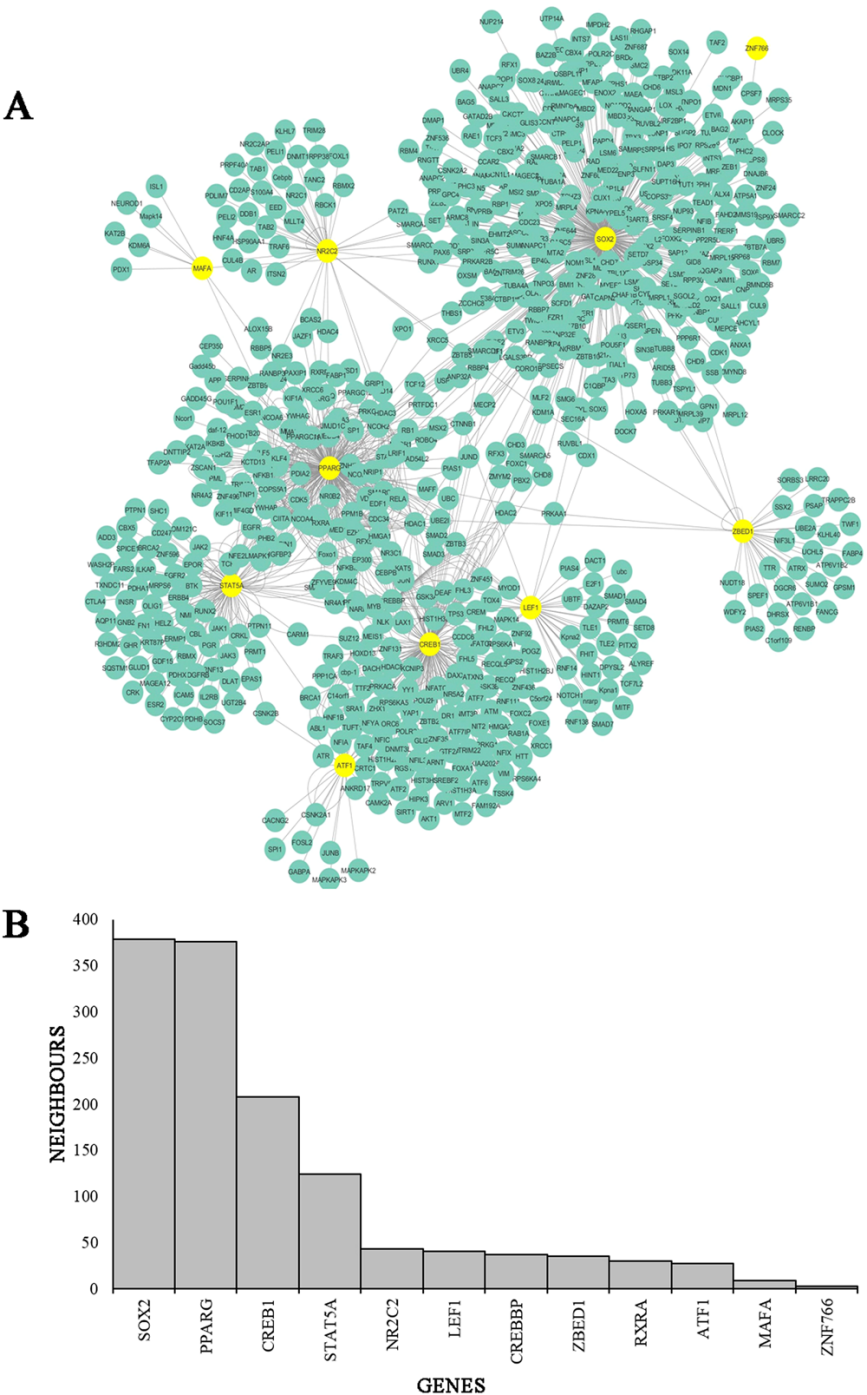
Network analysis of top 12 genes with their key interactions for MCF7. (**A**) Genes were represented as nodes while edges were used to represent interactions between genes. The top genes were highlighted in yellow. (**B**) Top 12 genes were plotted with name of genes on the x-axis and number of neighbors on the y-axis.

The protein-protein interaction network for MCF7 was visualized as displayed in Fig. 9. The network contained a total of 739 nodes and 1225 edges. The top 12 genes identified from the ChIP-Seq data were *SOX2* (SRY-Box 2), with the highest weight, *PPRAG* (Peroxisome Proliferator Activated Receptor Gamma), *CREB1* (CAMP Responsive Element Binding Protein 1), *STAT5A* (Signal Transducer And Activator Of Transcription 5A),*NR2C2* (Nuclear Receptor Subfamily 2 Group C Member 2), *LEF1* (Lymphoid Enhancer Binding Factor 1), *CREBBP* (CREB Binding Protein), *ZBED1* (Zinc Finger BED-Type Containing 1), *RXRA* (Retinoid X Receptor Alpha), *ATF1* (Activating Transcription Factor 1), *MAFA* (MAF BZIP Transcription Factor A) and *ZNF766* (Zinc Finger Protein 766) (Fig. 9A), out of which 2 were identified from the PPI network interaction network. The motifs with lowest Z-score and p-value were selected and a graph was plotted for name of genes versus the number of neighbors (Fig. 9B).

**Figure 10:**
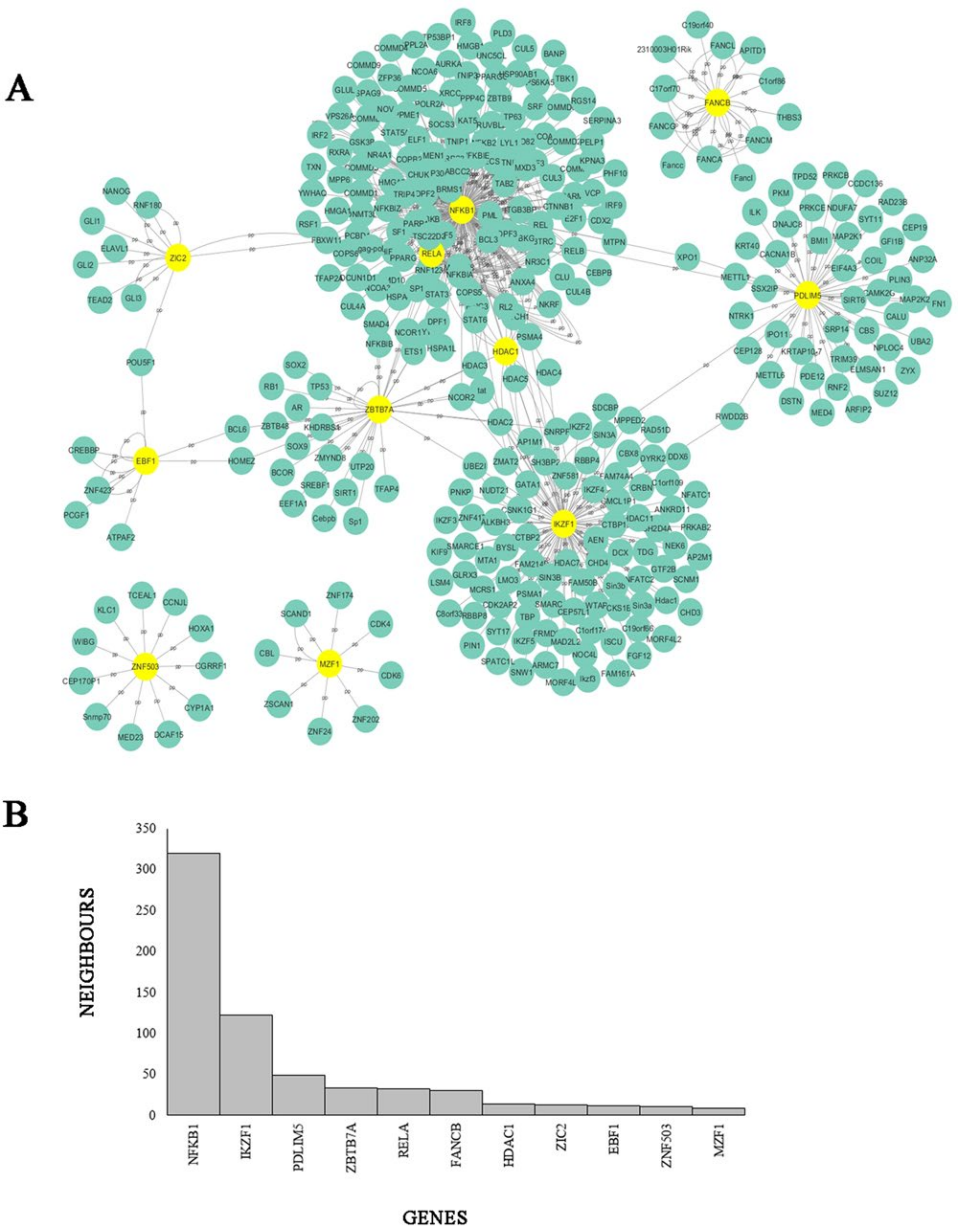
Network analysis of top 11 genes with their key interactions for MDA-MB-231. (**A**) Genes were represented as nodes while edges were used to represent interactions between genes. The top genes were highlighted in yellow. (**B**) Top 11 genes were plotted with name of genes on the x-axis and number of neighbors on the y-axis.

The protein-protein interaction network for MDA-MB-231 was found to contain 345 nodes and 596 edges as visualized in Fig. 10. The top 11 interacting genes present in MDA-MB-231, identified from the ChIP-Seq data were *NFKB1* (Nuclear Factor Kappa B Subunit 1), *IKZF1* (IKAROS Family Zinc Finger 1), *PDLIM5* (PDZ and LIM Domain 5), *ZBTB7A* (Zinc Finger and BTB Domain Containing 7 A), *RELA* (RELA Proto-Oncogene, *NF-KB* Subunit), *FANCB* (Fanconi Anemia Complementation Group B), *HDAC1* (Histone Deacetylase 1), *ZIC2* (Zic Family Member 2), *EBF1* (Early B-Cell Factor 1), *ZNF503* (Zinc Finger Protein 503) and *MZF1* (Myeloid Zinc Finger 1) (Fig. 10A). Figure 10B was used to represent the top motifs plotted against their neighbors.

### Pathway analysis of novel genes

**Figure 11:**
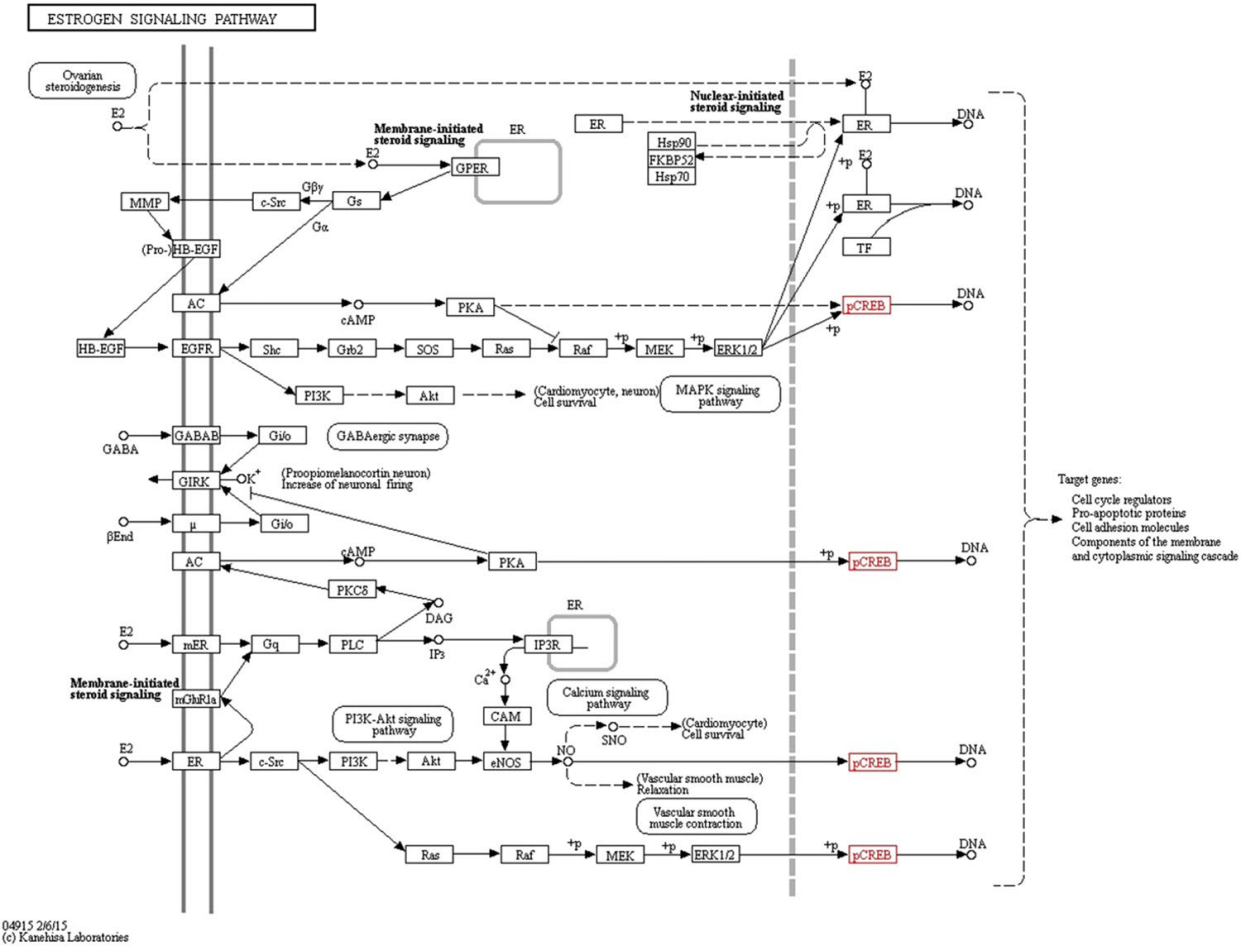
The estrogen signaling pathway. CREB highlighted in red is among the most prominent oncotarget involved in its signaling^29, 30^.

Out of several genes, two genes were identified to be prominent in their action, which might lead to the promotion of breast cancer.*CREB1* is a gene identified in MCF7 breast cancer, which belongs to a family of transcriptional factors, leading to the malignancy of breast. The *CREB* transcriptional factor is present in the estrogen signaling pathway and has been proposed as a target for breast cancer therapeutics. Aromatase, an enzyme responsible for the synthesis of estrogen has been found to induce breast cancer on over expression. In MCF7, the level of *CREB* was found to be over expressed. Therefore, the inhibition of *CREB* activity could potentially lead to the inhibition of aromatase, decreasing the level of estrogen. The estrogen signaling pathway is represented in Fig. 11.

*ZBTB7A* is an oncogenic transcriptional factor found to be over expressed in MDA-MB-231. It was found that *ZBTB7A*, also known as *Pokemon*, was over expressed in 86% of breast cancer and the expression was correlated with tumor size and lymph node metastasis, however, the mechanism of action is not exactly understood. *ZBTB7A* acts as an oncoprotein which inhibits the ARF/p53 pathway by regulating several genes, out of which one is Survivin, an anti-apoptotic protein and a negative prognostic indicator of breast cancer.

**Figure 12:**
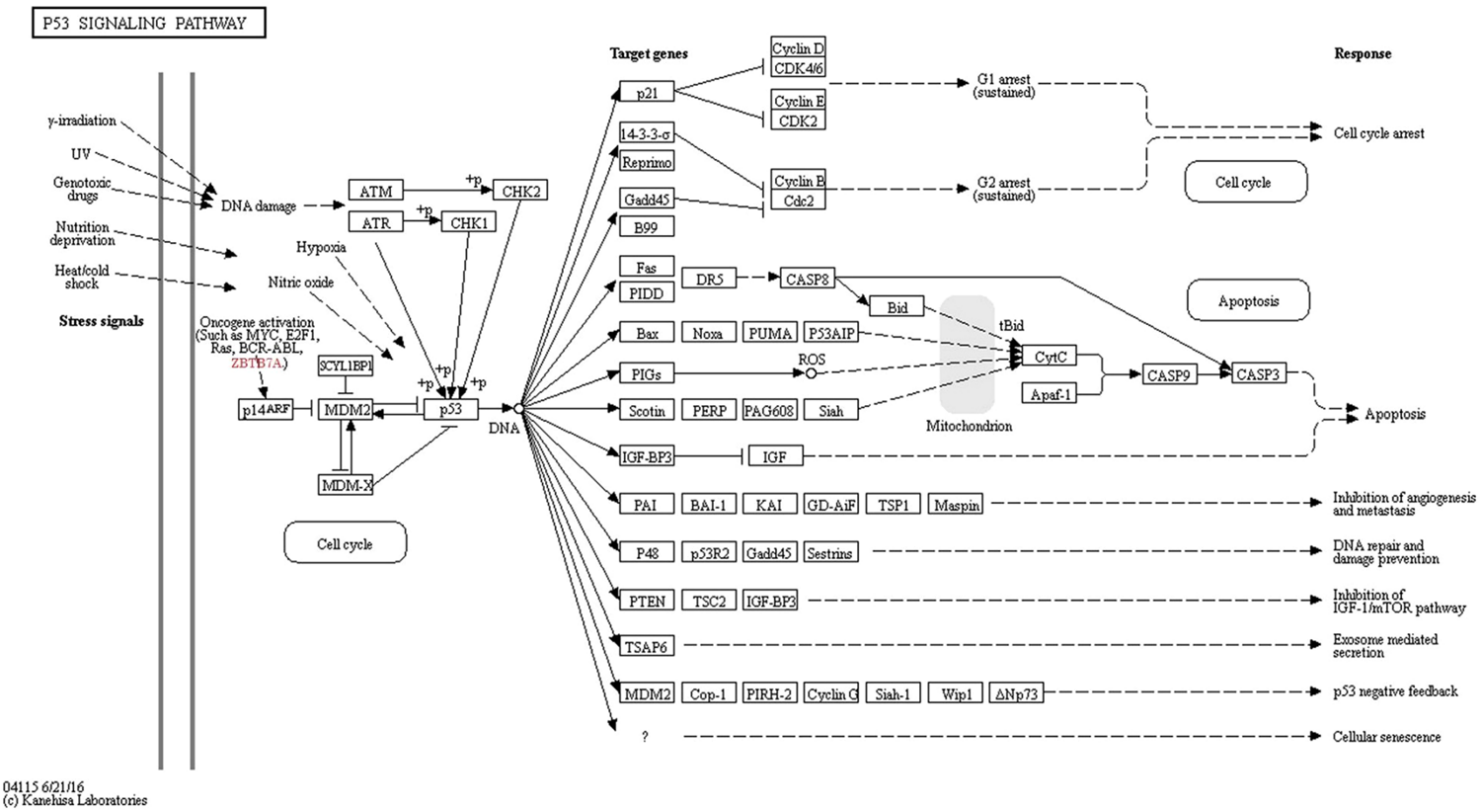
The ARF/p53 pathway. *ZBTB7A* (highlighted in red) is a part of the oncogenic activators responsible for triggering the ARF pathway^29, 30^.

ARF itself is not prominent in normal breast cancer cell type, however its expression was found to be elevated in the case of breast cancer cells by the action of oncogene activators such as *ZBTB7A*, which induces its transcription activity. This in turn antagonizes the activity of MDM2.Studies have also suggested that *ZBTB7A* acts as an upstream inducer of survivin breast cancer. Thus, *ZBTB7A* suppresses the ARF/p53 pathway and activates the survivin pathway. The ARF/p53 pathway is described in Fig. 12.

## Conclusion

Breast cancer is the most common female cancer worldwide representing nearly a quarter (23%) of all cancers in women. An early detection in order to improve breast cancer outcome and survival remains the cornerstone of breast cancer control. In this study, we identified an array of key genes related to Breast cancer by the analysis of ChIP-Seq data. These genes include *SOX2* (SRY-Box 2), with the highest weight, *PPRAG* (Peroxisome Proliferator Activated Receptor Gamma), *CREB1* (CAMP Responsive Element Binding Protein 1), *STAT5A* (Signal Transducer And Activator Of Transcription 5A), *NR2C2* (Nuclear Receptor Subfamily 2 Group C Member 2), *LEF1* (Lymphoid Enhancer Binding Factor 1), *CREBBP* (CREB Binding Protein), *ZBED1* (Zinc Finger BED-Type Containing 1), *RXRA* (Retinoid X Receptor Alpha), *ATF1* (Activating Transcription Factor 1), *MAFA* (MAF BZIP Transcription Factor A), *ZNF766* (Zinc Finger Protein 766), *NFKB1* (Nuclear Factor Kappa B Subunit 1), which was found to be elevated in both the cancer cell types, NFKB2 (Nuclear Factor Kappa B Subunit 2), *REL* (REL Proto-Oncogene, NF-KB Subunit), CENPB (Centromere Protein B), *NPAS4* (Neuronal PAS Domain Protein 4), *ZBTB7A* (Zinc Finger and BTB Domain Containing 7A), *PATZ1* (POZ/BTB and AT Hook Containing Zinc Finger 1) and *MZF1*, of which about one-half of them were located in the intergenic regions of chromosomes with differential peak annotations.

*SOX2* and *PPRAG* have already been reported to take part in the activation of breast cancer. *CREB1*, on the other hand, could prove to be a novel gene since its inhibition could potentially reduce the growth of cancer cells in the breast by keeping the estrogen levels at check. *LEF1* was found to be prominent in colon cancer and was also discovered in several types of MCF7 breast cancer. *MAFA*, another gene, was identified among the top genes present in breast cancer cell lines in this study. The gene *NFKB1* was found to be mutated and elevated in breast cancer, while *REL* is suggested to play some role as the *NFKB* genes belong to the *REL* family. *NPAS4* and *CENPB*, on the other hand, are both involved with breast cancer. *ZBTB7A*, though its proper function in the breast cancer pathway is unknown, acts as a tumor suppressor making it a novel target for combating breast cancer, while *PATZ1* possesses the potential for being a suitable candidate for the suppression of cancer. Other genes like *MZF1* have been targeted for the inhibition of invasive growth of breast cancer due to *MZF1* being one of the central node activated in breast cancer. These identified genes may prove to be helpful in enhancing the understanding the underlying molecular mechanism of the Breast cancer and also provides direction for future research. However, future research is needed to characterize their roles in detail and consequently develop effectual molecular target therapies for the treatment of Breast cancer.

## Methods

### Collection of data

Three Sequence Read Archive files, SRR3159917 for normal cell type (MCF10A), SRR3159923 for tumor related to MCF7 cancer cells and SRR3159929 for tumor related to MDA-MB-231 cancerous cells, were downloaded from Gene Expression Omnibus database with accession number [GSM2058903 for normal; GSM2058909 for MCF7 cell line and GSM2058915 for MDA-MB-231 cell line] of National Center for Biotechnology Information (NCBI)^18^.

Three well-established human mammary cell lines were utilized for studying the H3K27me3 gene suppression activity. These data were obtained from breast cancer patient tumor samples. Normal like subtype MCF10A and two epithelial subtypes, MCF7 (ESR/PGRþ) and MDA-MB-231 (ESR/PGR/HER2-) were selected for the study.

### Quality check and alignment

FASTQC was used to check the quality of reads for all respective data files of normal and two cancerous cell lines^19^. Initially, low quality reads were discarded and poor-quality ends of reads were trimmed to prevent filtering of high-quality reads during quality check^20^. ChIP-Seq reads were then aligned to the human genome (hg38) using the Bowtie2 tool^21–23^, which allows up to two mismatches in the alignment. For further analysis, exact matches for one or more regions were retained.

### Filtering and comparison of peak regions

Model-based analysis of ChIP-Seq (MACS2)^24^ was carried out for the identification of peaks (read-enriched regions) from BAM format files as well as comparison of peaks b/w cancerous and normal samples along with their annotation. A BAM file (*.bam) is the compressed binary version of a Sequence Alignment/Map (SAM) file that is used to represent aligned sequences. It runs on command line and designed to analyze data obtained via ChIP-Seq in eukaryotes, particularly in mammals^24, 25^. The parameters used in this study for calling peaks are mentioned as: (1) effective genome size = 2.70e + 09, (2) bandwidth = 300 bp and (3) P-value cutoff = 1.00e-10. To get into the insights of biological functioning and interpretation, fold enrichment scores for peaks above 2.00 were annotated to the hg38 refGenes data for known genomic sequence features (such as genes and transcription start site (TSS)). Based on the proximity of peaks to TSSs, genes were assigned to varying widths. The locations of those peaks were then extracted by MACS2. Identification of DNA motifs were carried out using MDSeqPos (P < 0.001) and validated using RSAT.

EaSeq^26^, is a tool developed for the analysis and visualization of ChIP-Seq data. EaSeq enables interactive exploration, visualization and analysis of genome-wide single-read sequencing data (mainly ChIP-seq). Visualizations can switch between individual genomic loci (as in a genome browser) and thousands of loci at a time as e.g. a plot of average signal, a scatter diagram, or a clustered heatmap. Subsets of loci can be inspected just by selecting them in a plot. It deals with the integration of so many analysis such as peak-finding, quantization, normalization, clustering, distance analysis, randomization, scoring, and normalization. EaSeq was used for annotation of gene data, comparison of peaks between normal and cancerous samples, plotting of enrichment analysis vs. –log (p-value), histogram of log (enrichment) and the density plot for the enrichment of all three datasets.

### Analysis of PPI network

Proteins (genes) work hand in hand in order to execute various biological functions. Proteins with more interacting neighbors are considered important in the entire process. Hence, PPI network analysis of genes obtained from MACS2 were carried out using Biological General Repository for Interaction Datasets (BioGRID)^27^, an open source database for physical and genetic interactions for all major model organism species (combined score >0.4). This network was further built by utilizing Cytoscape^28^, a software package accessible on the web for visualization of biological network, integration of data and generation of interaction network.

### Analysis of essential pathways

Pathway analysis is a technique used for identifying related proteins and genes within biological pathways. A pathway gives the various interactions among molecules in a cell related to its metabolism, gene regulation and signal transmission. KEGG pathway^29, 30^ is a molecular database which contains information related to biological pathways and the genes and proteins associated with it. KEGG pathway was used for the retrieval of pathways associated with novel genes identified from the present study.

## Electronic supplementary material

Supplementary Information
