# Author Correction: Identification of novel dysregulated key genes in Breast cancer through high throughput ChIP-Seq data analysis

**DOI:** 10.1038/s41598-020-61541-x

**Published:** 2020-03-06

**Authors:** Utkarsh Raj, Imlimaong Aier, Rahul Semwal, Pritish Kumar Varadwaj

**Affiliations:** Department of Bioinformatics & Applied Sciences, Indian Institute of Information Technology, Allahabad, Uttar Pradesh India

Correction to: *Scientific Reports* 10.1038/s41598-017-03534-x, published online 12 June 2017

This Article contains errors.

The “Collection of data” section is incomplete and does not make it clear which of the public datasets were used as control input against H3K27me3 treated sample files for peak identification. This information should be included as below.

In the Methods, the “Collection of data” subsection which reads:

“Three Sequence Read Archive files, SRR3159917 for normal cell type (MCF10A), SRR3159923 for tumor related to MCF7 cancer cells and SRR3159929 for tumor related to MDA-MB-231 cancerous cells, were downloaded from Gene Expression Omnibus database with accession number [GSM2058903 for normal; GSM2058909 for MCF7 cell line and GSM2058915 for MDA-MB-231 cell line] of National Center for Biotechnology Information (NCBI)^18^.

Three well-established human mammary cell lines were utilized for studying the H3K27me3 gene suppression activity. These data were obtained from breast cancer patient tumor samples. Normal like subtype MCF10A and two epithelial subtypes, MCF7 (ESR/PGRþ) and MDA-MB-231 (ESR/PGR/HER2-) were selected for the study.”

should read:

“Three Sequence Read Archive files, SRR3159917 for normal cell type (MCF10A), SRR3159923 for tumor related to MCF7 cancer cells and SRR3159929 for tumor related to MDA-MB-231 cancerous cells, were downloaded from Gene Expression Omnibus database with accession number [GSM2058903 for normal; GSM2058909 for MCF7 cell line and GSM2058915 for MDA-MB-231 cell line] of National Center for Biotechnology Information (NCBI)^18^. These input files were used as the control against H3K27Me3 treated sample files for peak identification. Similarly, the three H3K27Me3 treated sample Sequence Read Archive files, SRX1569788 for normal cell type (MCF10A), SRX1569794 for tumor related to MCF7 cancer cells and SRX1569800 for tumor related to MDA-MB-231 cancerous cells, were downloaded from Gene Expression Omnibus database with accession number GSM2058899 for normal; GSM2058905 for MCF7 cell line and GSM2058911 for MDA-MB-231 cell line. MCF10A cell line data was used only for the comparison purpose. All the data in the original manuscript were downloaded from Gene Expression Omnibus dataset series GSE77772.

Three well-established human mammary cell lines were utilized for studying the H3K27me3 gene suppression activity. These data were obtained from breast cancer patient tumor samples. Normal like subtype MCF10A and two epithelial subtypes, MCF7 (ESR/PGRþ) and MDA-MB-231 (ESR/PGR/HER2-) were selected for the study.”

The authors apologise for this error.

