# Supplementary material for: Identification of novel dysregulated key genes in Breast cancer through high throughput ChIP-Seq data analysis
Source: Sci Rep. 2017 Jun 12;7:3229. doi: 10.1038/s41598-017-03534-x (PMC5468232; doi:10.1038/s41598-017-03534-x)
Supplement: Supplementary file 1 — Supplementary Information [file 41598_2017_3534_MOESM1_ESM.pdf]

Identification of novel dysregulated key genes in Breast cancer through high throughput ChIP-Seq data analysis

Utkarsh Raj[$], Imlimaong Aier[$], Rahul Semwal, Pritish Kumar Varadwaj[*]

[*]Department of Bioinformatics & Applied Sciences, Indian Institute of Information Technology Allahabad, Uttar Pradesh, India

[$] Authors have contributed equally.

Table S1: Annotated gene table for 100 MCF7 peak regions sorted according to chromosome number.

Chromosome	Start	End	Name	Score	Fold enrichment	Minus log10(pvalue)	Minus log10(qvalue)	Summit position	Gene Name	Acc#	Relative position	dir
chr1	10017	10132	MACS2_peak_1	153	9.70613	19.83994	15.37945	65	DDX11L1	NR_046018	Upstream	+
chr1	121613177	121613285	MACS2_peak_21	237	12.0155	28.42089	23.75283	51	EMBP1	NR_003955	Downstream	+
chr1	121615164	121615269	MACS2_peak_22	159	9.43396	20.44173	15.96598	71	EMBP1	NR_003955	Downstream	+
chr1	121616030	121616151	MACS2_peak_23	437	17.55725	48.79121	43.7193	69	EMBP1	NR_003955	Downstream	+
chr1	121716985	121717085	MACS2_peak_24	96	7.5961	13.90991	9.64568	46	EMBP1	NR_003955	Downstream	+
chr1	121720714	121720815	MACS2_peak_25	107	8.01811	15.05372	10.74467	41	EMBP1	NR_003955	Downstream	+
chr1	121741472	121741575	MACS2_peak_26	112	7.50853	15.56878	11.24388	55	EMBP1	NR_003955	Downstream	+
chr1	121755094	121755339	MACS2_peak_27	91	7.25806	13.34932	9.1135	53	EMBP1	NR_003955	Downstream	+
chr1	121762047	121762206	MACS2_peak_28	131	8.36364	17.54986	13.15705	74	EMBP1	NR_003955	Downstream	+
chr1	121773335	121773458	MACS2_peak_29	178	9.62199	22.41519	17.8885	45	EMBP1	NR_003955	Downstream	+
chr1	121860424	121860540	MACS2_peak_30	205	11.15538	25.10539	20.51151	48	EMBP1	NR_003955	Downstream	+
chr1	121862419	121862523	MACS2_peak_31	144	9.16335	18.92366	14.49006	13	EMBP1	NR_003955	Downstream	+
chr1	121863287	121863409	MACS2_peak_32	409	17.26908	45.96629	40.94873	62	EMBP1	NR_003955	Downstream	+
chr1	121968468	121968613	MACS2_peak_33	96	7.5961	13.90991	9.64568	64	EMBP1	NR_003955	Downstream	+
chr1	122404323	122404485	MACS2_peak_34	216	11.81616	26.23304	21.6115	77	EMBP1	NR_003955	Downstream	+
chr1	122433694	122433886	MACS2_peak_35	202	11.34454	24.83766	20.25258	130	EMBP1	NR_003955	Downstream	+
chr1	122502987	122503087	MACS2_peak_36	91	6.80272	13.43073	9.19197	36	EMBP1	NR_003955	Downstream	+
chr1	122519371	122519477	MACS2_peak_37	84	6.36943	12.66081	8.45764	27	EMBP1	NR_003955	Downstream	+
chr1	122524643	122524756	MACS2_peak_38	77	6.16883	11.89781	7.72386	67	EMBP1	NR_003955	Downstream	+
chr1	122531634	122531748	MACS2_peak_39	120	7.20461	16.40275	12.04816	65	EMBP1	NR_003955	Downstream	+
chr1	122536006	122536134	MACS2_peak_40	412	14.24581	46.29644	41.27077	73	EMBP1	NR_003955	Downstream	+
chr1	122570108	122570212	MACS2_peak_41	130	8.86212	17.40662	13.01772	40	EMBP1	NR_003955	Downstream	+
chr1	122575839	122575939	MACS2_peak_42	85	7.1741	12.78938	8.57249	39	EMBP1	NR_003955	Downstream	+
chr1	122601304	122601430	MACS2_peak_43	152	9.30233	19.68285	15.22991	43	EMBP1	NR_003955	Downstream	+
chr1	122616041	122616141	MACS2_peak_44	45	5.48607	8.56886	4.58206	45	EMBP1	NR_003955	Downstream	+
chr1	122625052	122625180	MACS2_peak_45	70	6.4257	11.15712	7.0248	37	EMBP1	NR_003955	Downstream	+
chr1	122629300	122629412	MACS2_peak_46	52	5.69106	9.22937	5.21068	73	EMBP1	NR_003955	Downstream	+
chr1	122687814	122687922	MACS2_peak_47	47	5.19031	8.73624	4.74159	36	EMBP1	NR_003955	Downstream	+
chr1	122689959	122690193	MACS2_peak_48	172	9.27152	21.7803	17.27014	157	EMBP1	NR_003955	Downstream	+
chr1	122706320	122706427	MACS2_peak_49	134	8.51852	17.81866	13.41821	20	EMBP1	NR_003955	Downstream	+
chr1	122709974	122710081	MACS2_peak_50	59	5.9761	10.04485	5.97346	62	EMBP1	NR_003955	Downstream	+
chr1	122725539	122725649	MACS2_peak_51	75	6.75209	11.69346	7.52784	34	EMBP1	NR_003955	Downstream	+
chr1	122738082	122738236	MACS2_peak_52	33	4.83871	7.22796	3.35292	75	EMBP1	NR_003955	Downstream	+
chr1	122751924	122752038	MACS2_peak_53	161	9.87654	20.66016	16.17942	75	EMBP1	NR_003955	Downstream	+
chr1	122775134	122775270	MACS2_peak_54	150	8.8968	19.50351	15.05542	69	EMBP1	NR_003955	Downstream	+
chr1	122777441	122777574	MACS2_peak_55	251	11.76471	29.86721	25.1685	66	EMBP1	NR_003955	Downstream	+
chr1	122788454	122788586	MACS2_peak_56	91	6.80272	13.43073	9.19197	99	EMBP1	NR_003955	Downstream	+
chr1	122791937	122792084	MACS2_peak_57	105	7.63359	14.86465	10.56818	75	EMBP1	NR_003955	Downstream	+
chr1	122797032	122797147	MACS2_peak_58	115	7.95455	15.87534	11.54091	70	EMBP1	NR_003955	Downstream	+
chr1	122798276	122798406	MACS2_peak_59	143	8.79121	18.79988	14.36971	68	EMBP1	NR_003955	Downstream	+
chr1	122844059	122844163	MACS2_peak_60	119	8.23529	16.35291	11.99962	64	EMBP1	NR_003955	Downstream	+
chr1	122888893	122889188	MACS2_peak_61	194	10.21127	24.03849	19.47279	229	EMBP1	NR_003955	Downstream	+
chr1	122908360	122908471	MACS2_peak_62	119	7.91367	16.27971	11.92869	71	EMBP1	NR_003955	Downstream	+
chr1	122911339	122911465	MACS2_peak_63	134	8.24742	17.843	13.44166	81	EMBP1	NR_003955	Downstream	+
chr1	122916837	122916955	MACS2_peak_64	65	5.90278	10.65925	6.54919	72	EMBP1	NR_003955	Downstream	+
chr1	122919072	122919190	MACS2_peak_65	141	9.28412	18.61368	14.18773	69	EMBP1	NR_003955	Downstream	+
chr1	122950460	122950566	MACS2_peak_66	78	6.71937	12.0374	7.85797	81	EMBP1	NR_003955	Downstream	+
chr1	122981190	122981290	MACS2_peak_67	51	5.66802	9.1919	5.17489	41	EMBP1	NR_003955	Downstream	+
chr1	122999419	122999519	MACS2_peak_68	77	6.40569	11.91096	7.73583	55	EMBP1	NR_003955	Downstream	+
chr1	123001295	123001465	MACS2_peak_69	169	9.375	21.42109	16.91893	82	EMBP1	NR_003955	Downstream	+
chr1	123021985	123022102	MACS2_peak_70	168	8.76133	21.37031	16.86922	44	EMBP1	NR_003955	Downstream	+
chr1	123022950	123023075	MACS2_peak_71	65	5.4755	10.63971	6.53026	34	EMBP1	NR_003955	Downstream	+
chr1	123027245	123027352	MACS2_peak_72	160	8.61539	20.56522	16.08635	65	EMBP1	NR_003955	Downstream	+
chr1	123028955	123029068	MACS2_peak_73	140	8.33333	18.49883	14.0798	69	EMBP1	NR_003955	Downstream	+
chr1	123096416	123096556	MACS2_peak_74	278	13.33333	32.6524	27.89759	68	EMBP1	NR_003955	Downstream	+
chr1	123105075	123105225	MACS2_peak_75	277	13.22957	32.47152	27.71997	62	EMBP1	NR_003955	Downstream	+
chr1	123109879	123109980	MACS2_peak_76	99	7.53968	14.25601	9.97954	47	EMBP1	NR_003955	Downstream	+
chr1	123120384	123120484	MACS2_peak_77	85	7.1741	12.78938	8.57249	30	EMBP1	NR_003955	Downstream	+
chr1	123146920	123147030	MACS2_peak_78	164	9.09091	20.91035	16.42369	82	EMBP1	NR_003955	Downstream	+
chr1	123169697	123169839	MACS2_peak_79	67	6.27451	10.90719	6.78587	93	EMBP1	NR_003955	Downstream	+
chr1	123175953	123176142	MACS2_peak_80	49	5.51181	8.93723	4.93333	84	EMBP1	NR_003955	Downstream	+
chr1	123177064	123177236	MACS2_peak_81	104	7.57576	14.76632	10.47315	85	EMBP1	NR_003955	Downstream	+
chr1	123195673	123195796	MACS2_peak_82	87	7.00389	12.92471	8.70317	68	EMBP1	NR_003955	Downstream	+
chr1	123200473	123200598	MACS2_peak_83	80	6.82731	12.21666	8.03031	81	EMBP1	NR_003955	Downstream	+
chr1	123236843	123236967	MACS2_peak_84	191	10.3321	23.67655	19.11871	51	EMBP1	NR_003955	Downstream	+
chr1	123247326	123247431	MACS2_peak_85	107	8.01811	15.05372	10.74467	57	EMBP1	NR_003955	Downstream	+
chr1	123258810	123258911	MACS2_peak_86	94	7.19697	13.67831	9.4292	50	EMBP1	NR_003955	Downstream	+
chr1	123261171	123261271	MACS2_peak_87	97	7.16846	14.06793	9.79821	68	EMBP1	NR_003955	Downstream	+
chr1	123264285	123264475	MACS2_peak_88	82	6.46258	12.41532	8.22179	141	EMBP1	NR_003955	Downstream	+
chr1	123268192	123268301	MACS2_peak_89	114	8.16327	15.75753	11.42753	44	EMBP1	NR_003955	Downstream	+
chr1	123286017	123286166	MACS2_peak_90	111	7.96813	15.43008	11.10935	64	EMBP1	NR_003955	Downstream	+
chr1	123303876	123304012	MACS2_peak_91	190	10.97215	23.62614	19.0699	77	EMBP1	NR_003955	Downstream	+
chr1	123311443	123311559	MACS2_peak_92	65	6.33008	10.6236	6.51494	45	EMBP1	NR_003955	Downstream	+
chr1	123360744	123360916	MACS2_peak_93	116	7.7193	15.94015	11.60416	86	EMBP1	NR_003955	Downstream	+
chr1	123377508	123377666	MACS2_peak_94	75	6.75209	11.69346	7.52784	71	EMBP1	NR_003955	Downstream	+
chr1	123393617	123393744	MACS2_peak_95	54	5.61798	9.45349	5.42347	59	EMBP1	NR_003955	Downstream	+
chr1	123395176	123395283	MACS2_peak_96	99	7.02341	14.25568	9.97944	41	EMBP1	NR_003955	Downstream	+
chr1	123399004	123399154	MACS2_peak_97	145	8.0597	19.00079	14.56515	66	EMBP1	NR_003955	Downstream	+
chr1	123401220	123401321	MACS2_peak_98	104	7.07396	14.79131	10.49687	40	EMBP1	NR_003955	Downstream	+
chr1	123416206	123416360	MACS2_peak_99	176	9.49153	22.17989	17.66151	64	EMBP1	NR_003955	Downstream	+
chr1	123421970	123422087	MACS2_peak_100	115	7.69231	15.89278	11.55795	55	EMBP1	NR_003955	Downstream	+
chr1	123423002	123423102	MACS2_peak_101	62	5.90406	10.28958	6.20688	28	EMBP1	NR_003955	Downstream	+
chr1	123432535	123432647	MACS2_peak_102	170	9.44056	21.53814	17.03377	66	EMBP1	NR_003955	Downstream	+
chr1	123435588	123435732	MACS2_peak_103	89	6.66667	13.19086	8.95994	58	EMBP1	NR_003955	Downstream	+
chr1	123445776	123445879	MACS2_peak_104	40	4.96454	8.03415	4.09229	69	EMBP1	NR_003955	Downstream	+
chr1	123447481	123447583	MACS2_peak_105	73	6.16438	11.49477	7.34821	51	EMBP1	NR_003955	Downstream	+
chr1	123452739	123452869	MACS2_peak_106	60	6.04839	10.16343	6.08673	59	EMBP1	NR_003955	Downstream	+
chr1	123503953	123504112	MACS2_peak_107	157	8.99654	20.207	15.73739	68	EMBP1	NR_003955	Downstream	+
chr1	123507803	123507940	MACS2_peak_108	165	8.86076	21.02399	16.53478	50	EMBP1	NR_003955	Downstream	+
chr1	123513179	123513294	MACS2_peak_109	112	8.06452	15.59202	11.26629	47	EMBP1	NR_003955	Downstream	+
chr1	123548496	123548623	MACS2_peak_110	434	17.85714	48.54039	43.47359	43	EMBP1	NR_003955	Downstream	+
chr1	123558095	123558257	MACS2_peak_111	574	18.18182	62.84256	57.48838	80	EMBP1	NR_003955	Downstream	+
chr1	123564302	123564544	MACS2_peak_112	106	7.16612	14.95762	10.65806	52	EMBP1	NR_003955	Downstream	+
chr1	123567573	123567673	MACS2_peak_113	85	6.89655	12.74442	8.53705	35	EMBP1	NR_003955	Downstream	+
chr1	123580869	123580969	MACS2_peak_114	85	7.1741	12.78938	8.57249	37	EMBP1	NR_003955	Downstream	+
chr1	123598222	123598322	MACS2_peak_115	93	7.37705	13.54712	9.30344	37	EMBP1	NR_003955	Downstream	+
chr1	123603388	123603493	MACS2_peak_116	97	7.11744	13.97966	9.71282	58	EMBP1	NR_003955	Downstream	+
chr1	123604498	123604599	MACS2_peak_117	71	6.2963	11.3289	7.1895	84	EMBP1	NR_003955	Downstream	+
chr1	123605347	123605447	MACS2_peak_118	62	5.90406	10.28958	6.20688	35	EMBP1	NR_003955	Downstream	+
chr1	123641751	123641863	MACS2_peak_119	203	11.39241	24.91876	20.33171	58	EMBP1	NR_003955	Downstream	+
chr1	123647573	123647682	MACS2_peak_120	109	7.87402	15.27157	10.956	56	EMBP1	NR_003955	Downstream	+
chr1	123672703	123672842	MACS2_peak_121	55	5.51724	9.63462	5.58314	76	EMBP1	NR_003955	Downstream	+
chr1	123674744	123674848	MACS2_peak_122	90	6.48148	13.27956	9.04601	54	EMBP1	NR_003955	Downstream	+
chr1	123677919	123678026	MACS2_peak_123	143	7.9646	18.81958	14.38884	51	EMBP1	NR_003955	Downstream	+
chr1	123681869	123682116	MACS2_peak_124	83	6.06936	12.51587	8.31861	163	EMBP1	NR_003955	Downstream	+
chr1	123707342	123707478	MACS2_peak_125	290	13.61868	33.82025	29.04206	76	EMBP1	NR_003955	Downstream	+
chr1	123736075	123736208	MACS2_peak_126	138	9.09091	18.28952	13.87682	65	EMBP1	NR_003955	Downstream	+
chr1	123742199	123742485	MACS2_peak_127	63	6.19835	10.40875	6.32087	215	EMBP1	NR_003955	Downstream	+
chr1	123751168	123751345	MACS2_peak_128	353	13.39286	40.29886	35.38809	89	EMBP1	NR_003955	Downstream	+
chr1	123755622	123755775	MACS2_peak_129	334	13.10976	38.35706	33.48558	81	EMBP1	NR_003955	Downstream	+
chr1	123771358	123771496	MACS2_peak_130	178	10.55014	22.34688	17.82153	74	EMBP1	NR_003955	Downstream	+
chr1	123784492	123784665	MACS2_peak_131	33	4.81928	7.19684	3.32436	98	EMBP1	NR_003955	Downstream	+
chr1	123817776	123817893	MACS2_peak_132	116	7.7193	15.94015	11.60416	57	EMBP1	NR_003955	Downstream	+
chr1	123827936	123828055	MACS2_peak_133	101	7.63052	14.40816	10.12683	64	EMBP1	NR_003955	Downstream	+
chr1	123842000	123842127	MACS2_peak_134	103	7.49064	14.62123	10.33276	41	EMBP1	NR_003955	Downstream	+
chr1	123843055	123843201	MACS2_peak_135	156	9.22509	20.07892	15.61209	56	EMBP1	NR_003955	Downstream	+
chr1	123863314	123863445	MACS2_peak_136	96	7.5961	13.90991	9.64568	70	EMBP1	NR_003955	Downstream	+
chr1	123872458	123872608	MACS2_peak_137	82	6.99589	12.49562	8.29904	79	EMBP1	NR_003955	Downstream	+
chr1	123883571	123883736	MACS2_peak_138	133	8.48708	17.76423	13.36544	77	EMBP1	NR_003955	Downstream	+
chr1	123889737	123889862	MACS2_peak_139	162	9.61539	20.755	16.2717	73	EMBP1	NR_003955	Downstream	+
chr1	123919101	123919233	MACS2_peak_140	75	6.75209	11.69346	7.52784	50	EMBP1	NR_003955	Downstream	+
chr1	123957348	123957453	MACS2_peak_141	134	7.98722	17.86921	13.46695	53	EMBP1	NR_003955	Downstream	+
chr1	123988301	123988401	MACS2_peak_142	60	6.04839	10.16343	6.08673	43	EMBP1	NR_003955	Downstream	+
chr1	124007082	124007186	MACS2_peak_143	100	7.56972	14.30636	10.02841	30	EMBP1	NR_003955	Downstream	+
chr1	124022482	124022582	MACS2_peak_144	96	7.5961	13.90991	9.64568	80	EMBP1	NR_003955	Downstream	+
chr1	124031284	124031397	MACS2_peak_145	102	7.69231	14.51145	10.22647	76	EMBP1	NR_003955	Downstream	+
chr1	124038385	124038537	MACS2_peak_146	115	7.98479	15.92699	11.59139	86	EMBP1	NR_003955	Downstream	+

chr1	124054739	124054839	MACS2_peak_147	59	5.55556	10.05756	5.98577	65	EMBP1	NR_003955	Downstream	+
chr1	124061963	124062139	MACS2_peak_148	96	7.5961	13.90991	9.64568	95	EMBP1	NR_003955	Downstream	+
chr1	124071339	124071445	MACS2_peak_149	95	7.27969	13.81833	9.5635	37	EMBP1	NR_003955	Downstream	+
chr1	124084411	124084512	MACS2_peak_150	65	6.33008	10.6236	6.51494	17	EMBP1	NR_003955	Downstream	+
chr1	124098909	124099061	MACS2_peak_151	213	11.28405	25.92399	21.31132	68	EMBP1	NR_003955	Downstream	+
chr1	124119217	124119432	MACS2_peak_152	102	7.43494	14.52606	10.24063	52	EMBP1	NR_003955	Downstream	+
chr1	124123440	124123548	MACS2_peak_153	62	5.68562	10.28428	6.20226	50	EMBP1	NR_003955	Downstream	+
chr1	124129431	124129577	MACS2_peak_154	123	8.14815	16.68596	12.32295	80	EMBP1	NR_003955	Downstream	+
chr1	124168069	124168215	MACS2_peak_155	108	7.28477	15.17055	10.85751	75	EMBP1	NR_003955	Downstream	+
chr1	124169659	124169793	MACS2_peak_156	203	10.06494	24.92927	20.33906	54	EMBP1	NR_003955	Downstream	+
chr1	124178415	124178666	MACS2_peak_157	68	6.29921	10.9481	6.82456	195	EMBP1	NR_003955	Downstream	+
chr1	124181499	124181601	MACS2_peak_158	87	7.00389	12.92471	8.70317	41	EMBP1	NR_003955	Downstream	+
chr1	124197710	124197811	MACS2_peak_159	96	7.5961	13.90991	9.64568	29	EMBP1	NR_003955	Downstream	+
chr1	124204081	124204226	MACS2_peak_160	318	13.68421	36.67279	31.83624	72	EMBP1	NR_003955	Downstream	+
chr1	124225251	124225432	MACS2_peak_161	124	8.20896	16.79075	12.42432	82	EMBP1	NR_003955	Downstream	+
chr1	124250645	124250797	MACS2_peak_162	93	7.37705	13.54712	9.30344	59	EMBP1	NR_003955	Downstream	+
chr1	124268180	124268306	MACS2_peak_163	204	10.45296	25.05358	20.46068	54	EMBP1	NR_003955	Downstream	+
chr1	124270090	124270193	MACS2_peak_164	75	6.27178	11.68058	7.52562	17	EMBP1	NR_003955	Downstream	+
chr1	124281456	124281610	MACS2_peak_165	221	11.11111	26.82901	22.19486	85	EMBP1	NR_003955	Downstream	+
chr1	124290655	124290778	MACS2_peak_166	108	7.8125	15.16774	10.8548	54	EMBP1	NR_003955	Downstream	+
chr1	124297526	124297732	MACS2_peak_167	73	6.39098	11.48837	7.3421	162	EMBP1	NR_003955	Downstream	+
chr1	124306625	124306844	MACS2_peak_168	59	5.73477	10.00437	5.93498	67	EMBP1	NR_003955	Downstream	+
chr1	124309723	124309848	MACS2_peak_169	63	5.78231	10.45177	6.36175	86	EMBP1	NR_003955	Downstream	+
chr1	124315933	124316033	MACS2_peak_170	29	4.40678	6.80629	2.9587	59	EMBP1	NR_003955	Downstream	+
chr1	124320189	124320292	MACS2_peak_171	96	7.0922	13.93592	9.67063	46	EMBP1	NR_003955	Downstream	+
chr1	124330111	124330219	MACS2_peak_172	117	8.33333	16.04171	11.70237	79	EMBP1	NR_003955	Downstream	+
chr1	124331311	124331419	MACS2_peak_173	105	7.91667	14.88528	10.58836	47	EMBP1	NR_003955	Downstream	+
chr1	124347756	124347856	MACS2_peak_174	59	5.95238	10.0059	5.93621	70	EMBP1	NR_003955	Downstream	+
chr1	124377164	124377427	MACS2_peak_175	394	16	44.47373	39.48372	55	EMBP1	NR_003955	Downstream	+
chr1	124385184	124385323	MACS2_peak_176	152	9.33852	19.74486	15.29009	90	EMBP1	NR_003955	Downstream	+
chr1	124386343	124386457	MACS2_peak_177	131	8.62745	17.50642	13.11462	63	EMBP1	NR_003955	Downstream	+
chr1	124402506	124402630	MACS2_peak_178	167	9.88142	21.21155	16.71408	58	EMBP1	NR_003955	Downstream	+
chr1	124410747	124410847	MACS2_peak_179	85	6.89655	12.74442	8.53705	45	EMBP1	NR_003955	Downstream	+
chr1	124422784	124423040	MACS2_peak_180	92	6.84931	13.5125	9.27016	188	EMBP1	NR_003955	Downstream	+
chr1	124432206	124432329	MACS2_peak_181	85	7.1741	12.78938	8.57249	71	EMBP1	NR_003955	Downstream	+
chr1	124437320	124437495	MACS2_peak_182	145	9.2	18.98588	14.55054	88	EMBP1	NR_003955	Downstream	+
chr1	124438032	124438136	MACS2_peak_183	109	7.84314	15.21947	10.90522	75	EMBP1	NR_003955	Downstream	+
chr1	124450418	124450532	MACS2_peak_184	123	7.90378	16.74234	12.37748	50	EMBP1	NR_003955	Downstream	+
chr1	124469809	124469955	MACS2_peak_185	116	8.04598	16.03132	11.69251	80	EMBP1	NR_003955	Downstream	+
chr1	124486035	124486190	MACS2_peak_186	599	20.33898	65.35085	59.95988	84	EMBP1	NR_003955	Downstream	+
chr1	124493345	124493457	MACS2_peak_187	231	12.04819	27.85381	23.19711	56	EMBP1	NR_003955	Downstream	+
chr1	124511736	124511887	MACS2_peak_188	219	10.63123	26.56359	21.93476	65	EMBP1	NR_003955	Downstream	+
chr1	124512757	124513004	MACS2_peak_189	98	6.97674	14.17274	9.8999	70	EMBP1	NR_003955	Downstream	+
chr1	124514134	124514277	MACS2_peak_190	155	8.87372	19.9872	15.52245	84	EMBP1	NR_003955	Downstream	+
chr1	124524423	124524543	MACS2_peak_191	113	8.09717	15.64679	11.31989	71	EMBP1	NR_003955	Downstream	+
chr1	124540346	124540460	MACS2_peak_192	208	10.36789	25.487	20.8843	74	EMBP1	NR_003955	Downstream	+
chr1	124551087	124551229	MACS2_peak_193	163	9.65251	20.81891	16.33396	81	EMBP1	NR_003955	Downstream	+
chr1	124552922	124553026	MACS2_peak_194	85	6.92308	12.78903	8.57249	75	EMBP1	NR_003955	Downstream	+
chr1	124562406	124562513	MACS2_peak_195	104	7.85124	14.77645	10.48263	40	EMBP1	NR_003955	Downstream	+
chr1	124590765	124590873	MACS2_peak_196	75	6.31579	11.75645	7.58818	41	EMBP1	NR_003955	Downstream	+
chr1	124592947	124593061	MACS2_peak_197	164	9.12162	20.96585	16.47774	50	EMBP1	NR_003955	Downstream	+
chr1	124597806	124597969	MACS2_peak_198	109	7.33333	15.25735	10.94221	70	EMBP1	NR_003955	Downstream	+
chr1	124603575	124603866	MACS2_peak_199	183	10.22727	22.92229	18.3849	218	EMBP1	NR_003955	Downstream	+
chr1	124611121	124611263	MACS2_peak_200	115	7.69231	15.89278	11.55795	58	EMBP1	NR_003955	Downstream	+
chr1	124615120	124615271	MACS2_peak_201	77	6.20915	11.96933	7.79218	79	EMBP1	NR_003955	Downstream	+
chr1	124622369	124622513	MACS2_peak_202	80	6.59341	12.23159	8.04459	66	EMBP1	NR_003955	Downstream	+
chr1	124647253	124647358	MACS2_peak_203	114	7.63889	15.79887	11.46758	47	EMBP1	NR_003955	Downstream	+
chr1	124650487	124650598	MACS2_peak_204	111	7.69231	15.42527	11.10487	60	EMBP1	NR_003955	Downstream	+
chr1	124671718	124671822	MACS2_peak_205	83	6.79245	12.56896	8.36933	27	EMBP1	NR_003955	Downstream	+
chr1	124677013	124677255	MACS2_peak_206	65	6.33008	10.6236	6.51494	217	EMBP1	NR_003955	Downstream	+
chr1	124692630	124692730	MACS2_peak_207	87	6.53595	12.95882	8.73593	13	EMBP1	NR_003955	Downstream	+
chr1	124695316	124695431	MACS2_peak_208	132	7.86164	17.6381	13.24289	66	EMBP1	NR_003955	Downstream	+
chr1	124700701	124700817	MACS2_peak_209	240	11.80812	28.68387	24.0108	73	EMBP1	NR_003955	Downstream	+
chr1	124705812	124706056	MACS2_peak_210	86	6.97674	12.87917	8.65915	205	EMBP1	NR_003955	Downstream	+
chr1	124714874	124715034	MACS2_peak_211	140	8.91473	18.50008	14.08092	96	EMBP1	NR_003955	Downstream	+
chr1	124722907	124723013	MACS2_peak_212	113	7.86517	15.72241	11.39368	57	EMBP1	NR_003955	Downstream	+
chr1	124732265	124732496	MACS2_peak_213	643	18.32461	69.76807	64.30563	132	EMBP1	NR_003955	Downstream	+
chr1	124739311	124739428	MACS2_peak_214	68	5.67164	11.00147	6.87591	45	EMBP1	NR_003955	Downstream	+
chr1	124741362	124741500	MACS2_peak_215	497	16.28572	54.99177	49.7918	81	EMBP1	NR_003955	Downstream	+
chr1	124745588	124745724	MACS2_peak_216	123	7.35294	16.68578	12.32284	50	EMBP1	NR_003955	Downstream	+
chr1	124757619	124757774	MACS2_peak_217	270	12.83019	31.77135	27.03258	79	EMBP1	NR_003955	Downstream	+
chr1	124761942	124762061	MACS2_peak_218	41	4.82315	8.10778	4.16156	74	EMBP1	NR_003955	Downstream	+
chr1	124762819	124762941	MACS2_peak_219	107	7.47331	15.04603	10.74323	69	EMBP1	NR_003955	Downstream	+
chr1	124766744	124766853	MACS2_peak_220	64	5.64263	10.57662	6.48144	51	EMBP1	NR_003955	Downstream	+
chr1	124778970	124779207	MACS2_peak_221	207	10.6383	25.38571	20.78535	163	EMBP1	NR_003955	Downstream	+
chr1	124804424	124804532	MACS2_peak_222	118	8.44011	16.21965	11.87039	68	EMBP1	NR_003955	Downstream	+
chr1	124875878	124875987	MACS2_peak_223	96	7.5961	13.90991	9.64568	59	EMBP1	NR_003955	Downstream	+
chr1	124905437	124905621	MACS2_peak_224	96	7.0922	13.93592	9.67063	95	EMBP1	NR_003955	Downstream	+
chr1	124908813	124908978	MACS2_peak_225	102	7.16724	14.51013	10.22554	94	EMBP1	NR_003955	Downstream	+
chr1	124940597	124940698	MACS2_peak_226	101	7.63052	14.40816	10.12683	49	EMBP1	NR_003955	Downstream	+
chr1	125069444	125069571	MACS2_peak_227	167	9.88142	21.21155	16.71408	92	EMBP1	NR_003955	Downstream	+
chr1	125166261	125166365	MACS2_peak_228	184	5.15464	23.02285	18.48338	70	EMBP1	NR_003955	Downstream	+
chr1	125166912	125167027	MACS2_peak_229	376	7.3724	42.612	37.65781	52	EMBP1	NR_003955	Downstream	+
chr1	125167135	125167257	MACS2_peak_230	517	8.80682	56.98599	51.74411	44	EMBP1	NR_003955	Downstream	+
chr1	125167695	125168033	MACS2_peak_231	1116	14.09524	117.80161	111.62639	205	EMBP1	NR_003955	Downstream	+
chr1	125169945	125170204	MACS2_peak_232	140	4.33695	18.44728	14.03014	218	EMBP1	NR_003955	Downstream	+
chr1	125174022	125174170	MACS2_peak_233	638	7.30769	69.25733	63.80666	69	EMBP1	NR_003955	Downstream	+
chr1	125177332	125177433	MACS2_peak_234	99	2.19801	14.24762	9.9719	31	EMBP1	NR_003955	Downstream	+
chr1	125178863	125179436	MACS2_peak_235	325	2.8895	37.38799	32.53469	430	EMBP1	NR_003955	Downstream	+
chr1	125179591	125180703	MACS2_peak_236	330	2.89709	37.89738	33.03374	590	EMBP1	NR_003955	Downstream	+
chr1	125181537	125181890	MACS2_peak_237	163	2.34119	20.79454	16.31029	68	EMBP1	NR_003955	Downstream	+
chr1	125182120	125182712	MACS2_peak_238	376	3.13377	42.59783	37.64408	476	EMBP1	NR_003955	Downstream	+
chr1	125182952	125183117	MACS2_peak_239	383	3.17085	43.31749	38.3484	94	EMBP1	NR_003955	Downstream	+
chr1	125183533	125183823	MACS2_peak_240	282	2.88493	33.03716	28.27324	209	EMBP1	NR_003955	Downstream	+
chr1	1382650	1382756	MACS2_peak_10	107	8.01811	15.05372	10.74467	23	AURKAIP1	NM_017900	Upstream	-
chr1	143184625	143185365	MACS2_peak_241	1076	9.58617	113.78325	107.66439	537	LOC645166	NR_027354	Downstream	-
chr1	143185825	143185962	MACS2_peak_242	369	4.92958	41.90753	36.96734	58	LOC645166	NR_027354	Downstream	-
chr1	143187208	143187353	MACS2_peak_243	563	5.36518	61.66265	56.32976	70	LOC645166	NR_027354	Downstream	-
chr1	143187707	143187888	MACS2_peak_244	450	4.49506	50.18892	45.08778	76	LOC645166	NR_027354	Downstream	-
chr1	143190599	143190719	MACS2_peak_245	333	3.36961	38.16925	33.30058	54	LOC645166	NR_027354	Downstream	-
chr1	143191097	143191233	MACS2_peak_246	327	3.37751	37.61603	32.75766	62	LOC645166	NR_027354	Downstream	-
chr1	143191864	143191971	MACS2_peak_247	191	2.82034	23.66267	19.10529	63	LOC645166	NR_027354	Downstream	-
chr1	143192240	143192474	MACS2_peak_248	385	3.7329	43.4873	38.51506	171	LOC645166	NR_027354	Downstream	-
chr1	143192703	143193226	MACS2_peak_249	496	4.28255	54.85141	49.65366	382	LOC645166	NR_027354	Downstream	-
chr1	143193408	143193870	MACS2_peak_250	521	4.37454	57.35799	52.10812	322	LOC645166	NR_027354	Downstream	-
chr1	143194896	143195282	MACS2_peak_251	539	4.4439	59.26782	53.98338	114	LOC645166	NR_027354	Downstream	-
chr1	143195620	143195727	MACS2_peak_252	328	3.55086	37.74813	32.88756	62	LOC645166	NR_027354	Downstream	-
chr1	143200221	143200418	MACS2_peak_253	870	6.94087	92.88121	87.07584	114	LOC645166	NR_027354	Downstream	-
chr1	143200648	143200814	MACS2_peak_254	722	6.45535	77.83059	72.25176	81	LOC645166	NR_027354	Downstream	-
chr1	143201464	143201666	MACS2_peak_255	459	4.74621	51.07417	45.95132	94	LOC645166	NR_027354	Downstream	-
chr1	143202440	143202877	MACS2_peak_256	604	5.14728	65.82994	60.43182	238	LOC645166	NR_027354	Downstream	-
chr1	143203320	143203566	MACS2_peak_257	479	4.60761	53.12245	47.96181	69	LOC645166	NR_027354	Downstream	-
chr1	143206061	143206814	MACS2_peak_258	675	5.87219	73.0369	67.52438	72	LOC645166	NR_027354	Downstream	-
chr1	143207050	143207207	MACS2_peak_259	622	5.78603	67.64693	62.21824	75	LOC645166	NR_027354	Downstream	-
chr1	143211790	143211902	MACS2_peak_260	319	3.36737	36.79613	31.95755	53	LOC645166	NR_027354	Downstream	-
chr1	143212464	143212757	MACS2_peak_261	317	3.2644	36.56751	31.7332	89	LOC645166	NR_027354	Downstream	-
chr1	143212861	143212999	MACS2_peak_262	344	3.26577	39.36867	34.47772	78	LOC645166	NR_027354	Downstream	-
chr1	143213103	143213337	MACS2_peak_263	381	3.33274	43.11008	38.14513	163	LOC645166	NR_027354	Downstream	-
chr1	143214248	143214548	MACS2_peak_264	317	2.97125	36.60212	31.76713	205	LOC645166	NR_027354	Downstream	-
chr1	143214665	143214910	MACS2_peak_265	352	3.04671	40.11773	35.2104	115	LOC645166	NR_027354	Downstream	-
chr1	143215127	143215405	MACS2_peak_266	152	2.26431	19.68967	15.23652	239	LOC645166	NR_027354	Downstream	-
chr1	143215640	143215838	MACS2_peak_267	155	2.25058	20.00879	15.54361	133	LOC645166	NR_027354	Downstream	-
chr1	143216650	143216866	MACS2_peak_268	207	2.38581	25.35932	20.75947	170	LOC645166	NR_027354	Downstream	-
chr1	143217309	143217615	MACS2_peak_269	168	2.23081	21.29951	16.80005	219	LOC645166	NR_027354	Downstream	-
chr1	143217773	143217935	MACS2_peak_270	72	1.8474	11.36266	7.22196	130	LOC645166	NR_027354	Downstream	-
chr1	143218350	143218456	MACS2_peak_271	156	2.22222	20.14748	15.67931	43	LOC645166	NR_027354	Downstream	-
chr1	143218814	143218923	MACS2_peak_272	77	1.88902	11.90149	7.72737	60	LOC645166	NR_027354	Downstream	-
chr1	143219689	143219963	MACS2_peak_273	274	2.73152	32.22989	27.48295	94	LOC645166	NR_027354	Downstream	-
chr1	143220295	143220428	MACS2_peak_274	175	2.44918	22.11286	17.59586	70	LOC645166	NR_027354	Downstream	-

chr1	143220830	143221108	MACS2_peak_275	228	2.7226	27.5386	22.88978	186	LOC645166	NR_027354	Downstream	-
chr1	143221446	143221653	MACS2_peak_276	199	2.63344	24.52589	19.94871	124	LOC645166	NR_027354	Downstream	-
chr1	143222136	143222282	MACS2_peak_277	102	2.24676	14.56345	10.27682	44	LOC645166	NR_027354	Downstream	-
chr1	143222409	143222556	MACS2_peak_278	483	3.87581	53.50945	48.34184	75	LOC645166	NR_027354	Downstream	-
chr1	143222940	143223059	MACS2_peak_279	427	3.76632	47.83172	42.77847	60	LOC645166	NR_027354	Downstream	-
chr1	143227367	143227625	MACS2_peak_280	313	4.11682	36.13873	31.31275	198	LOC645166	NR_027354	Downstream	-
chr1	143227737	143228037	MACS2_peak_281	329	4.14402	37.85258	32.99028	231	LOC645166	NR_027354	Downstream	-
chr1	143230255	143230605	MACS2_peak_282	650	5.34648	70.48791	65.01665	76	LOC645166	NR_027354	Downstream	-
chr1	143231274	143231575	MACS2_peak_283	557	4.69083	61.05119	55.73198	140	LOC645166	NR_027354	Downstream	-
chr1	143232399	143232518	MACS2_peak_284	338	3.64493	38.75985	33.87858	55	LOC645166	NR_027354	Downstream	-
chr1	143232770	143233323	MACS2_peak_285	525	4.42832	57.80242	52.54385	176	LOC645166	NR_027354	Downstream	-
chr1	143233508	143233648	MACS2_peak_286	365	3.6984	41.48179	36.55099	88	LOC645166	NR_027354	Downstream	-
chr1	143235717	143235862	MACS2_peak_287	219	2.83393	26.60578	21.97622	82	LOC645166	NR_027354	Downstream	-
chr1	143237388	143237545	MACS2_peak_288	314	3.31492	36.24798	31.42041	102	LOC645166	NR_027354	Downstream	-
chr1	143238042	143238241	MACS2_peak_289	313	3.52477	36.20845	31.3815	135	LOC645166	NR_027354	Downstream	-
chr1	143238710	143238961	MACS2_peak_290	167	2.90761	21.19699	16.70014	50	LOC645166	NR_027354	Downstream	-
chr1	143239271	143239878	MACS2_peak_291	707	5.32932	76.27418	70.71727	258	LOC645166	NR_027354	Downstream	-
chr1	143240082	143240412	MACS2_peak_292	463	4.32447	51.49496	46.36551	213	LOC645166	NR_027354	Downstream	-
chr1	143245454	143245573	MACS2_peak_293	402	4.72822	45.20817	40.20393	65	LOC645166	NR_027354	Downstream	-
chr1	143245789	143245896	MACS2_peak_294	210	3.62595	25.69358	21.08629	56	LOC645166	NR_027354	Downstream	-
chr1	143246850	143247438	MACS2_peak_295	780	6.14982	83.67877	78.0058	503	LOC645166	NR_027354	Downstream	-
chr1	143249751	143250039	MACS2_peak_296	524	4.15846	57.73887	52.48172	93	LOC645166	NR_027354	Downstream	-
chr1	143251604	143251764	MACS2_peak_297	422	3.45438	47.29028	42.24784	64	LOC645166	NR_027354	Downstream	-
chr1	143251908	143252268	MACS2_peak_298	357	3.20624	40.6692	35.75192	62	LOC645166	NR_027354	Downstream	-
chr1	143252965	143253211	MACS2_peak_299	480	3.72162	53.24158	48.07811	64	LOC645166	NR_027354	Downstream	-
chr1	143253736	143253867	MACS2_peak_300	415	3.50746	46.58782	41.55721	63	LOC645166	NR_027354	Downstream	-
chr1	143254438	143254947	MACS2_peak_301	498	3.84096	55.05134	49.84948	420	LOC645166	NR_027354	Downstream	-
chr1	143255515	143256089	MACS2_peak_302	497	3.98323	54.97557	49.77599	407	LOC645166	NR_027354	Downstream	-
chr1	143256397	143256541	MACS2_peak_303	503	4	55.52079	50.30634	65	LOC645166	NR_027354	Downstream	-
chr1	143256772	143256933	MACS2_peak_304	523	4.18988	57.64102	52.387	77	LOC645166	NR_027354	Downstream	-
chr1	143262109	143263114	MACS2_peak_305	491	4.03138	54.34312	49.15579	615	LOC645166	NR_027354	Downstream	-
chr1	143263366	143263475	MACS2_peak_306	279	3.20122	32.68057	27.92525	50	LOC645166	NR_027354	Downstream	-
chr1	143263625	143263783	MACS2_peak_307	457	3.91086	50.84034	45.72225	82	LOC645166	NR_027354	Downstream	-
chr1	143264024	143264168	MACS2_peak_308	460	3.97217	51.22202	46.09716	79	LOC645166	NR_027354	Downstream	-
chr1	143264460	143264788	MACS2_peak_309	586	4.42796	64.01746	58.64354	191	LOC645166	NR_027354	Downstream	-
chr1	143265836	143265996	MACS2_peak_310	176	2.74336	22.15282	17.63493	61	LOC645166	NR_027354	Downstream	-
chr1	143266479	143267533	MACS2_peak_311	322	3.40482	37.11276	32.26831	75	LOC645166	NR_027354	Downstream	-
chr1	143270655	143270757	MACS2_peak_312	162	3.73057	20.7134	16.23149	76	LOC645166	NR_027354	Downstream	-
chr1	143275697	143275937	MACS2_peak_313	776	17.0778	83.26701	77.60081	84	LOC645166	NR_027354	Downstream	-
chr1	144103469	144103832	MACS2_peak_314	233	6.69192	28.05122	23.39112	211	SRGAP2D	NR_120535	Downstream	+
chr1	144103950	144104729	MACS2_peak_315	176	5.85242	22.18066	17.66219	373	SRGAP2D	NR_120535	Downstream	+
chr1	144852279	144852439	MACS2_peak_316	41	5.22088	8.14149	4.19349	21	SRGAP2-AS1	NR_104189	Within	+
chr1	148104177	148104281	MACS2_peak_317	79	5.88235	12.16226	7.97789	66	NBPF11	NM_001101663	Within	-
chr1	149023607	149023744	MACS2_peak_318	85	5.97826	12.74221	8.5352	78	NBPF9	NM_001277444	Downstream	-
chr1	152214722	152214824	MACS2_peak_319	38	3.50877	7.82101	3.89098	34	HRNR	NM_001009931	Within	-
chr1	152215821	152216205	MACS2_peak_320	50	3.79009	9.08013	5.06917	66	HRNR	NM_001009931	Within	-
chr1	161444898	161445048	MACS2_peak_321	59	4.79452	9.99717	5.9283	61	FCGR2A	NM_021642	Upstream	+
chr1	163552140	163552247	MACS2_peak_322	96	7.5961	13.90991	9.64568	69	LOC100422212	NR_104294	Upstream	-
chr1	16565439	16565564	MACS2_peak_15	78	5.26316	12.03773	7.85826	41	MIR3675	NR_037446	Downstream	+
chr1	16566163	16566329	MACS2_peak_16	41	4.19426	8.12701	4.18002	61	MIR3675	NR_037446	Downstream	+
chr1	168348969	168349074	MACS2_peak_323	118	8.44011	16.21965	11.87039	39	MIR557	NR_030284	Upstream	+
chr1	193832859	193832959	MACS2_peak_324	75	6.75209	11.69346	7.52784	19	LINC01031	NR_125789	Downstream	+
chr1	200816834	200816949	MACS2_peak_325	104	7.31707	14.77338	10.47989	58	CAMSAP2	NM_001297707	Within	+
chr1	202204532	202204643	MACS2_peak_326	85	5.59441	12.74793	8.54042	52	PTPRVP	NR_002930	Downstream	+
chr1	202205356	202205479	MACS2_peak_327	185	8.06452	23.09149	18.55018	78	PTPRVP	NR_002930	Downstream	+
chr1	206924095	206924508	MACS2_peak_328	39	4.33673	7.83165	3.90117	227	FCMR	NM_001142473	Upstream	-
chr1	210653911	210654017	MACS2_peak_329	130	8.86212	17.40662	13.01772	67	HHAT	NM_001170580	Within	+
chr1	228558119	228558304	MACS2_peak_330	492	18.05556	54.45123	49.26154	91	BTNL10	NM_001287262	Upstream	-
chr1	228622351	228622490	MACS2_peak_331	121	5.41796	16.51267	12.15488	67	RNA5S2	NR_023364	Upstream	-
chr1	228633560	228633662	MACS2_peak_332	61	4.12844	10.27334	6.19171	48	RNA5S15	NR_023377	Upstream	-
chr1	23392563	23392668	MACS2_peak_17	141	9.28412	18.61368	14.18773	56	TCEA3	NM_003196	Within	-
chr1	235078286	235078468	MACS2_peak_333	45	5.05051	8.49849	4.52995	73	LINC01348	NR_027454	Upstream	-
chr1	236097149	236097398	MACS2_peak_334	247	11.8705	29.42456	24.73459	93	NID1	NM_002508	Upstream	-
chr1	236713626	236713848	MACS2_peak_335	69	5.68862	11.03256	6.90564	70	ACTN2	NM_001278343	Within	+
chr1	236714012	236714175	MACS2_peak_336	52	5.12048	9.28589	5.26453	95	ACTN2	NM_001278343	Within	+
chr1	236714369	236714546	MACS2_peak_337	44	4.81928	8.41723	4.45365	78	ACTN2	NM_001278343	Within	+
chr1	236714689	236714952	MACS2_peak_338	79	6.06061	12.10036	7.91868	87	ACTN2	NM_001278343	Within	+
chr1	240184953	240185154	MACS2_peak_339	316	14.7541	36.47765	31.64552	77	FMN2	NM_020066	Within	+
chr1	246024914	246025069	MACS2_peak_340	52	5.71429	9.26714	5.24687	124	SMYD3	NM_022743	Within	-
chr1	246785848	246785951	MACS2_peak_341	96	7.36434	13.96121	9.69519	49	LINC01341	NR_015422	Upstream	+
chr1	248945902	248946098	MACS2_peak_342	167	9.88142	21.21155	16.71408	99	PGBD2	NM_001017434	Downstream	+
chr1	2652177	2652440	MACS2_peak_11	203	9.48276	24.96601	20.375	80	MMEL1	NM_033467	Upstream	-
chr1	2684023	2684195	MACS2_peak_12	78	5.83333	12.06878	7.88825	72	TTC34	NM_001242672	Within	-
chr1	2748975	2749092	MACS2_peak_13	84	6.34921	12.62443	8.42279	64	TTC34	NM_001242672	Within	-
chr1	31431773	31432143	MACS2_peak_18	446	15.56886	49.70407	44.6121	76	SERINC2	NM_001199039	Within	+
chr1	4144487	4144588	MACS2_peak_14	85	7.1741	12.78938	8.57249	59	LINC01346	NR_040065	Downstream	+
chr1	432860	433088	MACS2_peak_2	44	5.3719	8.38514	4.42332	55	OR4F29	NM_001005221	Downstream	-
chr1	53954675	53954776	MACS2_peak_19	42	5.24194	8.1755	4.22556	35	LRRC42	NM_052940	Within	+
chr1	629302	630005	MACS2_peak_3	591	6.05681	64.50813	59.12902	498	LOC101928626	NR_125957	Upstream	-
chr1	630345	630457	MACS2_peak_4	324	4.50041	37.26215	32.41282	48	MIR6723	NR_106781	Downstream	-
chr1	630781	631363	MACS2_peak_5	608	6.10813	66.24738	60.83922	510	MIR6723	NR_106781	Downstream	-
chr1	631888	632289	MACS2_peak_6	738	6.76568	79.48113	73.87846	327	MIR6723	NR_106781	Downstream	-
chr1	632522	632647	MACS2_peak_7	350	4.66172	39.95807	35.05578	65	MIR6723	NR_106781	Upstream	-
chr1	633408	633519	MACS2_peak_8	272	4.17183	32.00933	27.26657	58	MIR6723	NR_106781	Upstream	-
chr1	633914	634098	MACS2_peak_9	859	7.35537	91.75331	85.96548	106	MIR6723	NR_106781	Upstream	-
chr1	91387235	91387537	MACS2_peak_20	666	23.55212	72.09952	66.6048	92	HFM1	NM_001017975	Within	-
chr2	109199357	109199822	MACS2_peak_1225	360	12.40506	41.01761	36.09485	77	MIR4265	NR_036223	Upstream	-
chr2	10953689	10954110	MACS2_peak_1186	112	7.53425	15.61427	11.28816	337	KCNF1	NM_002236	Downstream	+
chr2	11113556	11113660	MACS2_peak_1187	63	6.01504	10.47547	6.3847	40	FLJ33534	NR_040080	Within	-
chr2	115439016	115439200	MACS2_peak_1226	118	8.44011	16.21965	11.87039	80	DPP10	NM_001178037	Within	+
chr2	120391412	120391555	MACS2_peak_1227	86	6.73759	12.89292	8.67244	49	INHBB	NM_002193	Downstream	+
chr2	135117479	135117594	MACS2_peak_1228	85	7.1741	12.78938	8.57249	33	RAB3GAP1	NM_001172435	Within	+
chr2	147369679	147369781	MACS2_peak_1229	96	7.5961	13.90991	9.64568	51	ACVR2A	NM_001278580	Upstream	+
chr2	161278659	161278770	MACS2_peak_1230	73	5.18018	11.49891	7.35225	63	LINC01806	NR_110163	Downstream	+
chr2	161279446	161279558	MACS2_peak_1231	144	7.02948	18.83183	14.40092	45	LINC01806	NR_110163	Downstream	+
chr2	161281217	161281360	MACS2_peak_1232	201	8.31461	24.69652	20.11499	79	LINC01806	NR_110163	Downstream	+
chr2	161281739	161281850	MACS2_peak_1233	88	5.58036	13.10048	8.87271	60	LINC01806	NR_110163	Downstream	+
chr2	161282407	161282511	MACS2_peak_1234	132	6.68151	17.60293	13.20884	52	LINC01806	NR_110163	Downstream	+
chr2	1861764	1861915	MACS2_peak_1181	186	10.38462	23.19511	18.65154	85	PXDN	NM_012293	Upstream	-
chr2	203987062	203987177	MACS2_peak_1235	141	9.28412	18.61368	14.18773	53	ICOS	NM_012092	Downstream	+
chr2	239644563	239644942	MACS2_peak_1236	186	10.38462	23.19511	18.65154	96	LOC150935	NR_037808	Upstream	+
chr2	240872253	240872436	MACS2_peak_1237	80	6.59341	12.23159	8.04459	88	AGXT	NM_000030	Within	+
chr2	241590491	241590682	MACS2_peak_1238	51	5.47445	9.2143	5.19637	139	THAP4	NM_001164356	Within	-
chr2	307609	307715	MACS2_peak_1180	50	5.57769	9.04477	5.03559	57	FAM150B	NM_001002919	Upstream	-
chr2	3180585	3181172	MACS2_peak_1182	188	9.22619	23.3465	18.80008	483	TSSC1	NM_001330530	Downstream	-
chr2	3221149	3221278	MACS2_peak_1183	107	7.47331	15.04603	10.74323	61	TSSC1	NM_001330530	Within	-
chr2	32916239	32916632	MACS2_peak_1188	251	11.76471	29.86721	25.1685	301	LINC00486	NR_027099	Within	+
chr2	5022649	5022790	MACS2_peak_1184	89	7.17131	13.20474	8.97317	75	LINC01249	NR_034134	Upstream	-
chr2	7488875	7488989	MACS2_peak_1185	65	6.33008	10.6236	6.51494	47	LOC100506274	NR_038432	Downstream	+
chr2	87398314	87398471	MACS2_peak_1189	74	5.81395	11.6447	7.49165	40	CYTOR	NR_024206	Upstream	+
chr2	87406962	87407077	MACS2_peak_1190	145	8.59107	18.96128	14.52655	64	CYTOR	NR_024206	Upstream	+
chr2	87417013	87417227	MACS2_peak_1191	31	3.78947	7.00815	3.14875	61	CYTOR	NR_024206	Upstream	+
chr2	87428550	87428666	MACS2_peak_1192	102	5.8952	14.57782	10.2905	77	CYTOR	NR_024206	Upstream	+
chr2	89822516	89822617	MACS2_peak_1193	100	5.45113	14.32652	10.04824	33	LOC101927050	NR_136329	Upstream	-
chr2	89826410	89826540	MACS2_peak_1194	301	6.39064	34.91748	30.11738	78	LOC101927050	NR_136329	Upstream	-
chr2	89828584	89828716	MACS2_peak_1195	409	6.8012	46.0073	40.98771	51	LOC101927050	NR_136329	Upstream	-
chr2	89829190	89829302	MACS2_peak_1196	243	5.19006	29.03117	24.34916	58	LOC101927050	NR_136329	Upstream	-
chr2	89829438	89829544	MACS2_peak_1197	159	4.30029	20.39853	15.92403	56	LOC101927050	NR_136329	Upstream	-
chr2	89830934	89831142	MACS2_peak_1198	741	9.23407	79.71465	74.10708	89	LOC101927050	NR_136329	Upstream	-
chr2	89833280	89833410	MACS2_peak_1199	365	6.07698	41.48077	36.55004	56	LOC101927050	NR_136329	Upstream	-
chr2	89836284	89836582	MACS2_peak_1200	350	6.26866	39.98987	35.08709	229	LOC101927050	NR_136329	Upstream	-
chr2	89838029	89838150	MACS2_peak_1201	261	5.7072	30.87466	26.15546	61	LOC101927050	NR_136329	Upstream	-
chr2	89838616	89838720	MACS2_peak_1202	92	3.85321	13.51773	9.27527	37	LOC101927050	NR_136329	Upstream	-
chr2	89840276	89840579	MACS2_peak_1203	468	8.42207	51.94748	46.80982	66	LOC101927050	NR_136329	Upstream	-
chr2	89841027	89841177	MACS2_peak_1204	802	11.8	85.94456	80.24205	68	LOC101927050	NR_136329	Upstream	-
chr2	90291780	90292063	MACS2_peak_1205	89	6.21469	13.19698	8.9658	150	LOC101927050	NR_136329	Upstream	-
chr2	90292260	90292373	MACS2_peak_1206	70	5.55556	11.15881	7.02632	40	LOC101927050	NR_136329	Upstream	-
chr2	90380704	90380846	MACS2_peak_1207	96	5.01618	13.94423	9.67881	63	LOC101927050	NR_136329	Upstream	-
chr2	90381068	90381190	MACS2_peak_1208	183	6.48567	22.89511	18.35865	62	LOC101927050	NR_136329	Upstream	-

chr2	90381604	90381719	MACS2_peak_1209	127	5.37791	17.15279	12.77583	64	LOC101927050	NR_136329	Upstream	-
chr2	90382565	90382745	MACS2_peak_1210	107	4.90196	15.01418	10.71261	84	LOC101927050	NR_136329	Upstream	-
chr2	90383748	90383857	MACS2_peak_1211	87	4.23729	12.93734	8.71532	67	LOC101927050	NR_136329	Upstream	-
chr2	90387747	90387849	MACS2_peak_1212	58	3.57942	9.95591	5.88913	50	LOC101927050	NR_136329	Upstream	-
chr2	90390539	90390804	MACS2_peak_1213	150	5.18433	19.49297	15.0453	196	LOC101927050	NR_136329	Upstream	-
chr2	90395522	90395748	MACS2_peak_1214	68	3.47349	11.00818	6.88229	67	LOC101927050	NR_136329	Upstream	-
chr2	90397483	90397636	MACS2_peak_1215	28	2.80193	6.69414	2.85356	54	LOC101927050	NR_136329	Upstream	-
chr2	90397748	90397913	MACS2_peak_1216	106	4.22535	14.97353	10.6734	43	LOC101927050	NR_136329	Upstream	-
chr2	90398204	90398348	MACS2_peak_1217	134	4.66059	17.89792	13.49467	74	LOC101927050	NR_136329	Upstream	-
chr2	90398458	90398711	MACS2_peak_1218	104	4.23117	14.69863	10.40772	109	LOC101927050	NR_136329	Upstream	-
chr2	90402159	90402261	MACS2_peak_1219	66	4.19162	10.73534	6.62157	58	LOC101927050	NR_136329	Upstream	-
chr2	91421910	91422096	MACS2_peak_1220	302	10.59908	35.06681	30.26388	88	LOC654342	NR_027238	Downstream	-
chr2	91422273	91422419	MACS2_peak_1221	207	8.62471	25.39209	20.79158	68	LOC654342	NR_027238	Downstream	-
chr2	92270852	92270954	MACS2_peak_1222	107	8.01811	15.05372	10.74467	58	ACTR3BP2	NR_027714	Downstream	+

Table S2: Annotated gene table for 100 MDA-MB-231 peak regions sorted according to chromosome number.

Chromosome	Start	End	Name	Score	Fold enrichment	Minus log(pvalue)	Minus log10(qvalue)	Summit position	Gene Name	Acc#	Relative position	dir
chr1	10009	10156	MACS2_peak_1	125	8.81734	16.84579	12.51139	62	DDX11L1	NR_046018	Upstream	+
chr1	1186274	1186373	MACS2_peak_10	69	6.613	11.0835	6.97562	25	TTLL10	NM_001130045	Within	+
chr1	121613171	121613277	MACS2_peak_27	131	8.32127	17.47608	13.12218	54	EMBP1	NR_003955	Downstream	+
chr1	121615163	121615266	MACS2_peak_28	157	8.96181	20.14495	15.71244	44	EMBP1	NR_003955	Downstream	+
chr1	121616033	121616249	MACS2_peak_29	471	17.28696	52.22697	47.10224	47	EMBP1	NR_003955	Downstream	+
chr1	121716971	121717085	MACS2_peak_30	186	11.02167	23.13957	18.62452	54	EMBP1	NR_003955	Downstream	+
chr1	121720711	121720815	MACS2_peak_31	125	8.81734	16.84579	12.51139	45	EMBP1	NR_003955	Downstream	+
chr1	121726308	121726413	MACS2_peak_32	148	9.69907	19.30642	14.89557	61	EMBP1	NR_003955	Downstream	+
chr1	121755109	121755409	MACS2_peak_33	124	7.92775	16.78482	12.45588	69	EMBP1	NR_003955	Downstream	+
chr1	121761412	121761537	MACS2_peak_34	57	5.84112	9.82278	5.78634	40	EMBP1	NR_003955	Downstream	+
chr1	121767587	121767685	MACS2_peak_35	41	4.78255	8.03743	4.10627	35	EMBP1	NR_003955	Downstream	+
chr1	121860427	121860544	MACS2_peak_36	212	11.2499	25.86484	21.28645	65	EMBP1	NR_003955	Downstream	+
chr1	121862421	121862525	MACS2_peak_37	182	10.12753	22.74871	18.24536	39	EMBP1	NR_003955	Downstream	+
chr1	121863287	121863407	MACS2_peak_38	433	17.31797	48.36509	43.31821	61	EMBP1	NR_003955	Downstream	+
chr1	121886779	121886982	MACS2_peak_39	136	9.23483	18.02724	13.65747	96	EMBP1	NR_003955	Downstream	+
chr1	122286120	122286231	MACS2_peak_40	103	7.4744	14.5935	10.33676	62	EMBP1	NR_003955	Downstream	+
chr1	122404302	122404438	MACS2_peak_41	405	15.74911	45.51648	40.52859	76	EMBP1	NR_003955	Downstream	+
chr1	122433682	122433890	MACS2_peak_42	205	10.83788	25.14697	20.58555	138	EMBP1	NR_003955	Downstream	+
chr1	122503852	122503951	MACS2_peak_43	90	6.4536	13.22851	9.03037	70	EMBP1	NR_003955	Downstream	+
chr1	122507920	122508023	MACS2_peak_44	90	6.69031	13.23266	9.03422	65	EMBP1	NR_003955	Downstream	+
chr1	122524651	122524766	MACS2_peak_45	103	6.74329	14.62402	10.36643	61	EMBP1	NR_003955	Downstream	+
chr1	122536001	122536131	MACS2_peak_46	429	15.08251	47.98608	42.94784	76	EMBP1	NR_003955	Downstream	+
chr1	122590925	122591024	MACS2_peak_47	116	8.29463	15.97712	11.67289	34	EMBP1	NR_003955	Downstream	+
chr1	122596164	122596328	MACS2_peak_48	190	10.27598	23.57795	19.05254	88	EMBP1	NR_003955	Downstream	+
chr1	122610249	122610353	MACS2_peak_49	31	4.84953	6.96861	3.11953	70	EMBP1	NR_003955	Downstream	+
chr1	122625057	122625162	MACS2_peak_50	95	7.01213	13.79685	9.57338	80	EMBP1	NR_003955	Downstream	+
chr1	122654049	122654149	MACS2_peak_51	84	6.82697	12.62721	8.45214	48	EMBP1	NR_003955	Downstream	+
chr1	122682610	122682731	MACS2_peak_52	46	4.91703	8.59103	4.62786	45	EMBP1	NR_003955	Downstream	+
chr1	122683208	122683331	MACS2_peak_53	129	7.68285	17.30646	12.95808	60	EMBP1	NR_003955	Downstream	+
chr1	122694986	122695094	MACS2_peak_54	107	6.94277	14.99794	10.72842	47	EMBP1	NR_003955	Downstream	+
chr1	122705180	122705303	MACS2_peak_55	41	4.81263	8.08956	4.15516	63	EMBP1	NR_003955	Downstream	+
chr1	122709779	122709878	MACS2_peak_56	92	7.07477	13.47074	9.25848	29	EMBP1	NR_003955	Downstream	+
chr1	122730660	122730877	MACS2_peak_57	36	4.84063	7.52333	3.63684	30	EMBP1	NR_003955	Downstream	+
chr1	122731820	122731981	MACS2_peak_58	27	4.4359	6.5792	2.776	12	EMBP1	NR_003955	Downstream	+
chr1	122756133	122756236	MACS2_peak_59	57	5.79688	9.74979	5.71706	72	EMBP1	NR_003955	Downstream	+
chr1	122798971	122799139	MACS2_peak_60	137	8.38574	18.08834	13.71354	107	EMBP1	NR_003955	Downstream	+
chr1	122806060	122806240	MACS2_peak_61	108	7.26408	15.13352	10.86012	109	EMBP1	NR_003955	Downstream	+
chr1	122809446	122809566	MACS2_peak_62	64	5.82032	10.51737	6.44065	53	EMBP1	NR_003955	Downstream	+
chr1	122815651	122815762	MACS2_peak_63	106	7.14843	14.92575	10.65898	68	EMBP1	NR_003955	Downstream	+
chr1	122818237	122818340	MACS2_peak_64	38	4.59587	7.7115	3.81233	43	EMBP1	NR_003955	Downstream	+
chr1	122822325	122822423	MACS2_peak_65	37	4.54133	7.61547	3.72275	38	EMBP1	NR_003955	Downstream	+
chr1	122846902	122847114	MACS2_peak_66	39	4.69454	7.88428	3.97363	11	EMBP1	NR_003955	Downstream	+
chr1	122853675	122853833	MACS2_peak_67	108	7.00493	15.11355	10.84074	63	EMBP1	NR_003955	Downstream	+
chr1	122862000	122862124	MACS2_peak_68	128	8.47001	17.23825	12.8917	63	EMBP1	NR_003955	Downstream	+
chr1	122865718	122865891	MACS2_peak_69	38	4.80934	7.77407	3.8703	36	EMBP1	NR_003955	Downstream	+
chr1	122872088	122872186	MACS2_peak_70	70	6.45213	11.2007	7.08792	34	EMBP1	NR_003955	Downstream	+
chr1	122887612	122887710	MACS2_peak_71	45	5.0844	8.5563	4.59543	83	EMBP1	NR_003955	Downstream	+
chr1	122897653	122897752	MACS2_peak_72	43	4.75936	8.31009	4.36361	54	EMBP1	NR_003955	Downstream	+
chr1	122904177	122904313	MACS2_peak_73	81	6.20232	12.35886	8.19248	92	EMBP1	NR_003955	Downstream	+
chr1	122911327	122911471	MACS2_peak_74	202	10.00904	24.82561	20.26973	79	EMBP1	NR_003955	Downstream	+
chr1	122913432	122913571	MACS2_peak_75	61	5.43347	10.20033	6.13866	63	EMBP1	NR_003955	Downstream	+
chr1	122916864	122916985	MACS2_peak_76	97	6.86723	13.97731	9.74763	42	EMBP1	NR_003955	Downstream	+
chr1	122920064	122920162	MACS2_peak_77	76	6.59477	11.82978	7.69102	48	EMBP1	NR_003955	Downstream	+
chr1	122935538	122935651	MACS2_peak_78	116	7.73994	15.97634	11.67228	35	EMBP1	NR_003955	Downstream	+
chr1	122943980	122944078	MACS2_peak_79	35	4.75389	7.38113	3.5045	45	EMBP1	NR_003955	Downstream	+
chr1	122951986	122952103	MACS2_peak_80	106	7.41368	14.94219	10.67466	34	EMBP1	NR_003955	Downstream	+
chr1	122966647	122966752	MACS2_peak_81	49	5.53622	8.9771	4.98365	56	EMBP1	NR_003955	Downstream	+
chr1	122969682	122969796	MACS2_peak_82	191	9.99586	23.65204	19.12506	42	EMBP1	NR_003955	Downstream	+
chr1	122981189	122981351	MACS2_peak_83	202	10.30574	24.7882	20.23336	84	EMBP1	NR_003955	Downstream	+
chr1	122998198	122998374	MACS2_peak_84	110	7.38354	15.34686	11.06623	85	EMBP1	NR_003955	Downstream	+
chr1	123001298	123001406	MACS2_peak_85	70	5.94334	11.10908	7.00051	77	EMBP1	NR_003955	Downstream	+
chr1	123009924	123010067	MACS2_peak_86	150	8.85645	19.43232	15.0183	51	EMBP1	NR_003955	Downstream	+
chr1	123013387	123013633	MACS2_peak_87	45	5.0844	8.5563	4.59543	38	EMBP1	NR_003955	Downstream	+
chr1	123022905	123023087	MACS2_peak_88	245	10.12308	29.16531	24.51373	77	EMBP1	NR_003955	Downstream	+
chr1	123027242	123027350	MACS2_peak_89	127	7.10003	17.12361	12.78092	51	EMBP1	NR_003955	Downstream	+
chr1	123048601	123048703	MACS2_peak_90	42	4.8895	8.22235	4.28101	46	EMBP1	NR_003955	Downstream	+
chr1	123055391	123055490	MACS2_peak_91	80	6.80163	12.17406	8.02176	42	EMBP1	NR_003955	Downstream	+
chr1	123075825	123075925	MACS2_peak_92	73	6.60884	11.45853	7.33449	62	EMBP1	NR_003955	Downstream	+
chr1	123096432	123096537	MACS2_peak_93	59	5.72779	9.99256	5.94806	42	EMBP1	NR_003955	Downstream	+
chr1	123105085	123105185	MACS2_peak_94	60	5.61315	10.15817	6.09834	40	EMBP1	NR_003955	Downstream	+
chr1	123108674	123108778	MACS2_peak_95	33	4.6214	7.16287	3.30054	72	EMBP1	NR_003955	Downstream	+
chr1	123115240	123115364	MACS2_peak_96	52	5.73629	9.30289	5.29259	55	EMBP1	NR_003955	Downstream	+
chr1	123120336	123120508	MACS2_peak_97	140	8.56164	18.39836	14.01581	92	EMBP1	NR_003955	Downstream	+
chr1	123130443	123130544	MACS2_peak_98	113	7.31041	15.67564	11.38064	53	EMBP1	NR_003955	Downstream	+
chr1	123145678	123145820	MACS2_peak_99	101	6.37687	14.3469	10.10402	59	EMBP1	NR_003955	Downstream	+
chr1	123146923	123147030	MACS2_peak_100	240	10.20296	28.73197	24.08885	58	EMBP1	NR_003955	Downstream	+
chr1	123148340	123148441	MACS2_peak_101	84	6.1043	12.58141	8.40808	43	EMBP1	NR_003955	Downstream	+
chr1	123153433	123153532	MACS2_peak_102	66	5.9399	10.72295	6.6371	41	EMBP1	NR_003955	Downstream	+
chr1	123159283	123159467	MACS2_peak_103	178	9.00252	22.36864	17.8745	77	EMBP1	NR_003955	Downstream	+
chr1	123169707	123169861	MACS2_peak_104	245	11.37352	29.15223	24.50078	73	EMBP1	NR_003955	Downstream	+
chr1	123176010	123176167	MACS2_peak_105	114	7.63465	15.79141	11.493	85	EMBP1	NR_003955	Downstream	+
chr1	123185072	123185236	MACS2_peak_106	27	4.4359	6.5792	2.776	37	EMBP1	NR_003955	Downstream	+
chr1	123195643	123195815	MACS2_peak_107	167	8.98357	21.25159	16.78815	96	EMBP1	NR_003955	Downstream	+
chr1	123200460	123200570	MACS2_peak_108	80	6.10948	12.18978	8.031	47	EMBP1	NR_003955	Downstream	+
chr1	123225781	123226018	MACS2_peak_109	197	10.34532	24.27752	19.73648	165	EMBP1	NR_003955	Downstream	+
chr1	123242280	123242384	MACS2_peak_110	53	5.58534	9.39923	5.38375	24	EMBP1	NR_003955	Downstream	+
chr1	123247330	123247441	MACS2_peak_111	91	7.25865	13.35029	9.14204	55	EMBP1	NR_003955	Downstream	+
chr1	123254502	123254600	MACS2_peak_112	78	6.91683	11.96289	7.81882	52	EMBP1	NR_003955	Downstream	+
chr1	123259649	123259848	MACS2_peak_113	67	5.79337	10.84472	6.75394	73	EMBP1	NR_003955	Downstream	+
chr1	123265152	123265263	MACS2_peak_114	115	7.40264	15.84352	11.54351	44	EMBP1	NR_003955	Downstream	+
chr1	123286016	123286173	MACS2_peak_115	146	8.3682	19.07022	14.66894	80	EMBP1	NR_003955	Downstream	+
chr1	123305763	123305861	MACS2_peak_116	74	6.42382	11.54356	7.41628	70	EMBP1	NR_003955	Downstream	+
chr1	123307767	123307888	MACS2_peak_117	122	7.82206	16.59722	12.27413	44	EMBP1	NR_003955	Downstream	+
chr1	123311411	123311536	MACS2_peak_118	144	8.83978	18.88441	14.48815	66	EMBP1	NR_003955	Downstream	+
chr1	123331452	123331550	MACS2_peak_119	83	6.75169	12.50007	8.32925	56	EMBP1	NR_003955	Downstream	+
chr1	123337838	123337953	MACS2_peak_120	136	8.65899	18.06122	13.69053	51	EMBP1	NR_003955	Downstream	+
chr1	123357820	123357921	MACS2_peak_121	126	8.06395	17.02531	12.6858	24	EMBP1	NR_003955	Downstream	+
chr1	123372510	123372718	MACS2_peak_122	89	6.89855	13.16975	8.97411	167	EMBP1	NR_003955	Downstream	+
chr1	123377726	123377824	MACS2_peak_123	55	5.87791	9.53258	5.51048	25	EMBP1	NR_003955	Downstream	+
chr1	123393579	123393747	MACS2_peak_124	181	9.74998	22.64488	18.1445	76	EMBP1	NR_003955	Downstream	+
chr1	123395180	123395278	MACS2_peak_125	90	6.71231	13.27152	9.07162	23	EMBP1	NR_003955	Downstream	+
chr1	123401221	123401328	MACS2_peak_126	166	8.89962	21.09616	16.63651	40	EMBP1	NR_003955	Downstream	+
chr1	123403344	123403448	MACS2_peak_127	111	7.40791	15.39022	11.10819	60	EMBP1	NR_003955	Downstream	+
chr1	123415310	123415408	MACS2_peak_128	70	6.0016	11.21118	7.09793	45	EMBP1	NR_003955	Downstream	+
chr1	123416264	123416392	MACS2_peak_129	72	6.10128	11.38512	7.26406	61	EMBP1	NR_003955	Downstream	+
chr1	123417316	123417535	MACS2_peak_130	53	5.16215	9.36077	5.34768	41	EMBP1	NR_003955	Downstream	+
chr1	123432541	123432651	MACS2_peak_131	130	7.49366	17.42836	13.07586	59	EMBP1	NR_003955	Downstream	+
chr1	123435592	123435721	MACS2_peak_132	132	7.36578	17.65489	13.29572	59	EMBP1	NR_003955	Downstream	+
chr1	123443424	123443531	MACS2_peak_133	86	6.00175	12.78774	8.60586	39	EMBP1	NR_003955	Downstream	+
chr1	123443747	123443875	MACS2_peak_134	103	6.49526	14.58042	10.32429	71	EMBP1	NR_003955	Downstream	+
chr1	123445703	123445857	MACS2_peak_135	101	6.37687	14.3469	10.10402	90	EMBP1	NR_003955	Downstream	+
chr1	123447456	123447602	MACS2_peak_136	181	8.35485	22.67731	18.17629	80	EMBP1	NR_003955	Downstream	+

chr1	123466782	123467000	MACS2_peak_137	80	6.55881	12.17269	8.02056	185	EMBP1	NR_003955	Downstream	+
chr1	123502758	123502856	MACS2_peak_138	48	5.05401	8.83263	4.85547	68	EMBP1	NR_003955	Downstream	+
chr1	123503955	123504072	MACS2_peak_139	99	6.72043	14.14548	9.90942	62	EMBP1	NR_003955	Downstream	+
chr1	123524648	123524746	MACS2_peak_140	82	6.48331	12.45154	8.28252	40	EMBP1	NR_003955	Downstream	+
chr1	123526588	123526722	MACS2_peak_141	55	5.45963	9.53602	5.51359	46	EMBP1	NR_003955	Downstream	+
chr1	123548497	123548623	MACS2_peak_142	373	14.39263	42.23498	37.31713	60	EMBP1	NR_003955	Downstream	+
chr1	123551655	123551785	MACS2_peak_143	125	6.7379	16.83049	12.50018	71	EMBP1	NR_003955	Downstream	+
chr1	123554256	123554361	MACS2_peak_144	132	6.46831	17.56053	13.20418	61	EMBP1	NR_003955	Downstream	+
chr1	123556420	123556582	MACS2_peak_145	451	12.55162	50.26278	45.17804	76	EMBP1	NR_003955	Downstream	+
chr1	123558104	123558565	MACS2_peak_146	424	12.2605	47.47979	42.4513	78	EMBP1	NR_003955	Downstream	+
chr1	123564293	123564392	MACS2_peak_147	89	6.64673	13.15557	8.96069	49	EMBP1	NR_003955	Downstream	+
chr1	123585955	123586057	MACS2_peak_148	117	7.76672	16.02324	11.71743	53	EMBP1	NR_003955	Downstream	+
chr1	123592904	123593019	MACS2_peak_149	18	3.82604	5.57514	1.89022	86	EMBP1	NR_003955	Downstream	+
chr1	123598617	123598715	MACS2_peak_150	77	6.37665	11.86111	7.72128	47	EMBP1	NR_003955	Downstream	+
chr1	123605343	123605449	MACS2_peak_151	83	7.02189	12.53855	8.36646	35	EMBP1	NR_003955	Downstream	+
chr1	123618661	123618875	MACS2_peak_152	59	5.54143	10.03284	5.97877	139	EMBP1	NR_003955	Downstream	+
chr1	123628315	123628415	MACS2_peak_153	101	7.63175	14.41021	10.16464	34	EMBP1	NR_003955	Downstream	+
chr1	123641759	123641862	MACS2_peak_154	170	9.75244	21.53801	17.06674	22	EMBP1	NR_003955	Downstream	+
chr1	123682060	123682182	MACS2_peak_155	120	7.16497	16.32667	12.01189	62	EMBP1	NR_003955	Downstream	+
chr1	123707352	123707485	MACS2_peak_156	325	13.32207	37.38467	32.55992	60	EMBP1	NR_003955	Downstream	+
chr1	123720077	123720224	MACS2_peak_157	243	10.61571	28.94989	24.30226	75	EMBP1	NR_003955	Downstream	+
chr1	123735110	123735208	MACS2_peak_158	102	6.90651	14.48703	10.23424	50	EMBP1	NR_003955	Downstream	+
chr1	123736057	123736251	MACS2_peak_159	170	8.83231	21.50504	17.03473	67	EMBP1	NR_003955	Downstream	+
chr1	123743209	123743316	MACS2_peak_160	125	8.00891	16.9283	12.59155	32	EMBP1	NR_003955	Downstream	+
chr1	123751167	123751268	MACS2_peak_161	70	5.75235	11.1489	7.0384	32	EMBP1	NR_003955	Downstream	+
chr1	123755628	123755791	MACS2_peak_162	295	12.07438	34.32538	29.56068	77	EMBP1	NR_003955	Downstream	+
chr1	123767965	123768166	MACS2_peak_163	122	8.3692	16.57934	12.25673	145	EMBP1	NR_003955	Downstream	+
chr1	123775473	123775586	MACS2_peak_164	74	6.18344	11.52781	7.40106	71	EMBP1	NR_003955	Downstream	+
chr1	123784434	123784668	MACS2_peak_165	131	8.05477	17.49887	13.14435	61	EMBP1	NR_003955	Downstream	+
chr1	123785908	123786014	MACS2_peak_166	107	7.21737	15.04976	10.77872	49	EMBP1	NR_003955	Downstream	+
chr1	123790292	123790395	MACS2_peak_167	38	4.80934	7.77407	3.8703	51	EMBP1	NR_003955	Downstream	+
chr1	123798464	123798569	MACS2_peak_168	98	7.18494	14.09641	9.86203	39	EMBP1	NR_003955	Downstream	+
chr1	123803584	123803686	MACS2_peak_169	44	5.36968	8.38157	4.43103	72	EMBP1	NR_003955	Downstream	+
chr1	123831845	123831943	MACS2_peak_170	98	7.15973	14.05285	9.82042	59	EMBP1	NR_003955	Downstream	+
chr1	123851022	123851251	MACS2_peak_171	288	12.02145	33.56779	28.81938	134	EMBP1	NR_003955	Downstream	+
chr1	123862396	123862494	MACS2_peak_172	53	5.58534	9.39923	5.38375	33	EMBP1	NR_003955	Downstream	+
chr1	123863366	123863479	MACS2_peak_173	119	7.90343	16.2619	11.94937	66	EMBP1	NR_003955	Downstream	+
chr1	123872783	123872883	MACS2_peak_174	70	5.98205	11.17696	7.06521	40	EMBP1	NR_003955	Downstream	+
chr1	123883555	123883705	MACS2_peak_175	95	7.01213	13.79685	9.57338	64	EMBP1	NR_003955	Downstream	+
chr1	123889758	123889860	MACS2_peak_176	91	6.73446	13.31059	9.10899	63	EMBP1	NR_003955	Downstream	+
chr1	123924486	123924604	MACS2_peak_177	61	5.64972	10.22186	6.15907	39	EMBP1	NR_003955	Downstream	+
chr1	123958288	123958393	MACS2_peak_178	59	5.33871	10.02809	5.97422	62	EMBP1	NR_003955	Downstream	+
chr1	123959505	123959665	MACS2_peak_179	342	13.49909	39.10832	34.25089	77	EMBP1	NR_003955	Downstream	+
chr1	123974513	123974621	MACS2_peak_180	46	4.91703	8.59103	4.62786	29	EMBP1	NR_003955	Downstream	+
chr1	123984141	123984269	MACS2_peak_181	113	7.53218	15.61063	11.32141	72	EMBP1	NR_003955	Downstream	+
chr1	123998886	123998995	MACS2_peak_182	80	6.35579	12.22811	8.06743	62	EMBP1	NR_003955	Downstream	+
chr1	124026292	124026426	MACS2_peak_183	81	6.88259	12.30826	8.14408	58	EMBP1	NR_003955	Downstream	+
chr1	124038407	124038569	MACS2_peak_184	117	7.54544	16.10186	11.79386	74	EMBP1	NR_003955	Downstream	+
chr1	124051702	124051808	MACS2_peak_185	87	6.30176	12.94902	8.76151	37	EMBP1	NR_003955	Downstream	+
chr1	124061946	124062154	MACS2_peak_186	110	7.38354	15.34686	11.06623	145	EMBP1	NR_003955	Downstream	+
chr1	124097016	124097122	MACS2_peak_187	45	5.0844	8.5563	4.59543	57	EMBP1	NR_003955	Downstream	+
chr1	124098948	124099078	MACS2_peak_188	56	5.53365	9.66265	5.63371	62	EMBP1	NR_003955	Downstream	+
chr1	124117495	124117673	MACS2_peak_189	202	9.97747	24.76696	20.21289	66	EMBP1	NR_003955	Downstream	+
chr1	124129462	124129565	MACS2_peak_190	64	6.02364	10.48985	6.41404	58	EMBP1	NR_003955	Downstream	+
chr1	124164408	124164506	MACS2_peak_191	53	5.19306	9.41616	5.39991	20	EMBP1	NR_003955	Downstream	+
chr1	124167358	124167462	MACS2_peak_192	75	6.02028	11.63286	7.50235	75	EMBP1	NR_003955	Downstream	+
chr1	124178501	124178662	MACS2_peak_193	111	7.70699	15.45058	11.16619	117	EMBP1	NR_003955	Downstream	+
chr1	124184466	124184579	MACS2_peak_194	98	7.45566	14.11493	9.87989	35	EMBP1	NR_003955	Downstream	+
chr1	124204093	124204210	MACS2_peak_195	273	12.24323	32.02579	27.31257	58	EMBP1	NR_003955	Downstream	+
chr1	124209021	124209129	MACS2_peak_196	62	5.29661	10.30522	6.23851	68	EMBP1	NR_003955	Downstream	+
chr1	124210959	124211092	MACS2_peak_197	188	8.96961	23.3953	18.8742	61	EMBP1	NR_003955	Downstream	+
chr1	124225283	124225439	MACS2_peak_198	202	9.97747	24.76696	20.21289	58	EMBP1	NR_003955	Downstream	+
chr1	124237638	124237760	MACS2_peak_199	178	9.87347	22.30421	17.81157	51	EMBP1	NR_003955	Downstream	+
chr1	124259761	124259869	MACS2_peak_200	110	6.89774	15.36002	11.07889	46	EMBP1	NR_003955	Downstream	+
chr1	124262774	124262908	MACS2_peak_201	146	7.84841	19.09234	14.69062	65	EMBP1	NR_003955	Downstream	+
chr1	124270095	124270216	MACS2_peak_202	130	8.00213	17.40432	13.05264	72	EMBP1	NR_003955	Downstream	+
chr1	124277900	124278006	MACS2_peak_203	124	7.92775	16.78482	12.45588	58	EMBP1	NR_003955	Downstream	+
chr1	124283374	124283592	MACS2_peak_204	59	5.50625	9.97117	5.92798	60	EMBP1	NR_003955	Downstream	+
chr1	124288236	124288345	MACS2_peak_205	100	7.02482	14.25819	10.01815	33	EMBP1	NR_003955	Downstream	+
chr1	124290663	124290780	MACS2_peak_206	134	8.53306	17.84382	13.47899	42	EMBP1	NR_003955	Downstream	+
chr1	124298835	124298933	MACS2_peak_207	41	5.01181	8.11298	4.17755	73	EMBP1	NR_003955	Downstream	+
chr1	124309703	124309849	MACS2_peak_208	50	4.96989	9.01344	5.01806	98	EMBP1	NR_003955	Downstream	+
chr1	124311779	124311910	MACS2_peak_209	105	7.08079	14.80366	10.54045	56	EMBP1	NR_003955	Downstream	+
chr1	124318155	124318301	MACS2_peak_210	131	7.80031	17.5247	13.16924	59	EMBP1	NR_003955	Downstream	+
chr1	124327241	124327408	MACS2_peak_211	20	3.92413	5.73896	2.03597	143	EMBP1	NR_003955	Downstream	+
chr1	124331308	124331427	MACS2_peak_212	170	9.75244	21.53801	17.06674	59	EMBP1	NR_003955	Downstream	+
chr1	124343441	124343720	MACS2_peak_213	137	7.84835	18.10371	13.72851	115	EMBP1	NR_003955	Downstream	+
chr1	124344382	124344541	MACS2_peak_214	86	6.2285	12.81318	8.63029	57	EMBP1	NR_003955	Downstream	+
chr1	124351624	124351761	MACS2_peak_215	73	6.40012	11.50374	7.37773	58	EMBP1	NR_003955	Downstream	+
chr1	124362898	124362996	MACS2_peak_216	69	6.12878	11.04525	6.94596	42	EMBP1	NR_003955	Downstream	+
chr1	124367022	124367120	MACS2_peak_217	51	5.05681	9.17107	5.16718	56	EMBP1	NR_003955	Downstream	+
chr1	124370050	124370284	MACS2_peak_218	179	9.05578	22.47037	17.97395	172	EMBP1	NR_003955	Downstream	+
chr1	124377161	124377363	MACS2_peak_219	590	18.52396	64.35867	59.01814	76	EMBP1	NR_003955	Downstream	+
chr1	124386351	124386449	MACS2_peak_220	64	6.04595	10.52711	6.45005	37	EMBP1	NR_003955	Downstream	+
chr1	124397246	124397443	MACS2_peak_221	43	5.34803	8.34669	4.3982	41	EMBP1	NR_003955	Downstream	+
chr1	124402479	124402627	MACS2_peak_222	119	7.93136	16.31048	11.99643	85	EMBP1	NR_003955	Downstream	+
chr1	124406454	124406624	MACS2_peak_223	94	6.69557	13.66869	9.44997	61	EMBP1	NR_003955	Downstream	+
chr1	124412941	124413039	MACS2_peak_224	44	5.15654	8.35323	4.4043	22	EMBP1	NR_003955	Downstream	+
chr1	124420950	124421160	MACS2_peak_225	789	20.40613	84.6301	78.95018	117	EMBP1	NR_003955	Downstream	+
chr1	124429954	124430052	MACS2_peak_226	70	5.96263	11.14293	7.03271	43	EMBP1	NR_003955	Downstream	+
chr1	124437293	124437445	MACS2_peak_227	136	8.09743	18.07082	13.69676	64	EMBP1	NR_003955	Downstream	+
chr1	124438045	124438160	MACS2_peak_228	93	6.65399	13.59348	9.37703	44	EMBP1	NR_003955	Downstream	+
chr1	124450828	124451086	MACS2_peak_229	103	6.74329	14.62402	10.36643	41	EMBP1	NR_003955	Downstream	+
chr1	124456549	124456682	MACS2_peak_230	135	8.02105	17.93121	13.56402	75	EMBP1	NR_003955	Downstream	+
chr1	124467033	124467217	MACS2_peak_231	79	6.27353	12.08318	7.93463	97	EMBP1	NR_003955	Downstream	+
chr1	124469799	124469929	MACS2_peak_232	145	8.31574	18.97384	14.57494	46	EMBP1	NR_003955	Downstream	+
chr1	124476926	124477120	MACS2_peak_233	205	9.83405	25.07389	20.51382	33	EMBP1	NR_003955	Downstream	+
chr1	124486038	124486198	MACS2_peak_234	581	19.80132	63.48911	58.16368	84	EMBP1	NR_003955	Downstream	+
chr1	124499454	124499606	MACS2_peak_235	213	9.68992	25.94565	21.36557	73	EMBP1	NR_003955	Downstream	+
chr1	124511731	124511895	MACS2_peak_236	152	9.01291	19.70778	15.28617	72	EMBP1	NR_003955	Downstream	+
chr1	124518942	124519228	MACS2_peak_237	94	7.20625	13.69404	9.47406	67	EMBP1	NR_003955	Downstream	+
chr1	124527101	124527206	MACS2_peak_238	137	8.12321	18.11784	13.74227	74	EMBP1	NR_003955	Downstream	+
chr1	124539536	124539654	MACS2_peak_239	129	7.95018	17.31078	12.96209	61	EMBP1	NR_003955	Downstream	+
chr1	124540371	124540479	MACS2_peak_240	89	6.66845	13.19401	8.9972	60	EMBP1	NR_003955	Downstream	+
chr1	124554008	124554109	MACS2_peak_241	81	6.62983	12.29353	8.1301	46	EMBP1	NR_003955	Downstream	+
chr1	124562069	124562187	MACS2_peak_242	77	5.96659	11.92756	7.7851	38	EMBP1	NR_003955	Downstream	+
chr1	124562415	124562525	MACS2_peak_243	149	8.2478	19.35645	14.94432	58	EMBP1	NR_003955	Downstream	+
chr1	124568507	124568745	MACS2_peak_244	144	7.47192	18.8265	14.43202	67	EMBP1	NR_003955	Downstream	+
chr1	124570212	124570644	MACS2_peak_245	170	8.54993	21.49269	17.02291	59	EMBP1	NR_003955	Downstream	+
chr1	124590686	124590792	MACS2_peak_246	69	5.90512	11.04193	6.94297	28	EMBP1	NR_003955	Downstream	+
chr1	124597093	124597222	MACS2_peak_247	91	6.26995	13.30218	9.10131	79	EMBP1	NR_003955	Downstream	+
chr1	124597798	124597986	MACS2_peak_248	52	4.93692	9.28429	5.27529	43	EMBP1	NR_003955	Downstream	+
chr1	124615087	124615228	MACS2_peak_249	172	8.38972	21.70002	17.22453	80	EMBP1	NR_003955	Downstream	+
chr1	124623434	124623540	MACS2_peak_250	98	6.68043	14.07157	9.83821	58	EMBP1	NR_003955	Downstream	+
chr1	124639245	124639406	MACS2_peak_251	186	9.44703	23.20961	18.69239	84	EMBP1	NR_003955	Downstream	+
chr1	124642317	124642457	MACS2_peak_252	186	8.84809	23.15317	18.63769	74	EMBP1	NR_003955	Downstream	+
chr1	124646511	124646612	MACS2_peak_253	126	7.28781	17.02977	12.6899	45	EMBP1	NR_003955	Downstream	+
chr1	124650488	124650595	MACS2_peak_254	118	7.3095	16.13697	11.82812	58	EMBP1	NR_003955	Downstream	+
chr1	124671715	124671813	MACS2_peak_255	80	5.49582	12.16277	8.0111	26	EMBP1	NR_003955	Downstream	+
chr1	124673082	124673367	MACS2_peak_256	181	7.88324	22.68597	18.18443	194	EMBP1	NR_003955	Downstream	+
chr1	124677071	124677247	MACS2_peak_257	117	7.04622	16.09764	11.78997	92	EMBP1	NR_003955	Downstream	+
chr1	124690522	124690653	MACS2_peak_258	72	5.4659	11.35683	7.23699	66	EMBP1	NR_003955	Downstream	+
chr1	124692621	124692724	MACS2_peak_259	131	7.53623	17.51016	13.15522	51	EMBP1	NR_003955	Downstream	+
chr1	124695328	124695475	MACS2_peak_260	62	5.48212	10.28832	6.222	62	EMBP1	NR_003955	Downstream	+
chr1	124700700	124700814	MACS2_peak_261	173	9.63185	21.87808	17.39507	74	EMBP1	NR_003955	Downstream	+
chr1	124714677	124715035	MACS2_peak_262	136	8.04635	17.97751	13.60917	292	EMBP1	NR_003955	Downstream	+
chr1	124729304	124729402	MACS2_peak_263	58	5.0881	9.90943	5.86906	24	EMBP1	NR_003955	Downstream	+
chr1	124732321	124732480	MACS2_peak_264	368	11.84294	41.77486	36.86584	85	EMBP1	NR_003955	Downstream	+
chr1	124736081	124736205	MACS2_peak_265	158	7.06538	20.3159	15.87918	72	EMBP1	NR_003955	Downstream	+

chr1	124739329	124739448	MACS2_peak_266	88	5.53318	12.99781	8.80897	64	EMBP1	NR_003955	Downstream	+
chr1	124740211	124740363	MACS2_peak_267	186	8.09773	23.16521	18.64928	82	EMBP1	NR_003955	Downstream	+
chr1	124741362	124741501	MACS2_peak_268	605	16.57118	65.86894	60.50084	77	EMBP1	NR_003955	Downstream	+
chr1	124745539	124745670	MACS2_peak_269	71	5.59065	11.22535	7.11156	54	EMBP1	NR_003955	Downstream	+
chr1	124757658	124757782	MACS2_peak_270	460	16.32448	51.18187	46.07763	41	EMBP1	NR_003955	Downstream	+
chr1	124763953	124764087	MACS2_peak_271	62	5.31112	10.33251	6.26435	69	EMBP1	NR_003955	Downstream	+
chr1	124766740	124766851	MACS2_peak_272	109	6.59109	15.20273	10.92677	69	EMBP1	NR_003955	Downstream	+
chr1	124768731	124768933	MACS2_peak_273	141	7.57781	18.55671	14.16988	55	EMBP1	NR_003955	Downstream	+
chr1	124774864	124774983	MACS2_peak_274	108	6.5741	15.16878	10.89406	53	EMBP1	NR_003955	Downstream	+
chr1	124778994	124779308	MACS2_peak_275	131	7.53623	17.51016	13.15522	61	EMBP1	NR_003955	Downstream	+
chr1	124804412	124804532	MACS2_peak_276	181	8.88456	22.68314	18.18174	69	EMBP1	NR_003955	Downstream	+
chr1	124902869	124902983	MACS2_peak_277	50	5.18235	9.05704	5.05947	36	EMBP1	NR_003955	Downstream	+
chr1	124903537	124903638	MACS2_peak_278	50	5.14966	9.00005	5.00562	45	EMBP1	NR_003955	Downstream	+
chr1	124905441	124905657	MACS2_peak_279	214	9.71706	25.99931	21.41769	143	EMBP1	NR_003955	Downstream	+
chr1	124908786	124908982	MACS2_peak_280	127	7.32807	17.10813	12.76574	114	EMBP1	NR_003955	Downstream	+
chr1	124925762	124925961	MACS2_peak_281	70	6.0016	11.21118	7.09793	78	EMBP1	NR_003955	Downstream	+
chr1	124934428	124934582	MACS2_peak_282	29	3.79549	6.76522	2.94805	138	EMBP1	NR_003955	Downstream	+
chr1	124937411	124937509	MACS2_peak_283	53	4.47322	9.31487	5.30424	23	EMBP1	NR_003955	Downstream	+
chr1	124940599	124940697	MACS2_peak_284	85	6.21045	12.77961	8.59816	67	EMBP1	NR_003955	Downstream	+
chr1	125080241	125080347	MACS2_peak_285	53	4.82233	9.39486	5.37976	27	EMBP1	NR_003955	Downstream	+
chr1	125108999	125109107	MACS2_peak_286	43	5.13799	8.32253	4.37517	57	EMBP1	NR_003955	Downstream	+
chr1	125166266	125166366	MACS2_peak_287	130	4.16646	17.37693	13.02636	58	EMBP1	NR_003955	Downstream	+
chr1	125166913	125167024	MACS2_peak_288	366	6.75308	41.60079	36.69607	58	EMBP1	NR_003955	Downstream	+
chr1	125167137	125167259	MACS2_peak_289	529	8.26884	58.23017	52.99808	68	EMBP1	NR_003955	Downstream	+
chr1	125167716	125168037	MACS2_peak_290	1262	14.07412	132.58952	126.27695	179	EMBP1	NR_003955	Downstream	+
chr1	125168141	125168247	MACS2_peak_291	170	4.73021	21.50895	17.03854	77	EMBP1	NR_003955	Downstream	+
chr1	125169953	125170212	MACS2_peak_292	257	5.45533	30.40868	25.72915	220	EMBP1	NR_003955	Downstream	+
chr1	125174014	125174165	MACS2_peak_293	673	7.07784	72.81708	67.33215	77	EMBP1	NR_003955	Downstream	+
chr1	125177332	125177449	MACS2_peak_294	154	2.4262	19.89665	15.47025	37	EMBP1	NR_003955	Downstream	+
chr1	125178842	125179433	MACS2_peak_295	284	2.69825	33.17147	28.42984	334	EMBP1	NR_003955	Downstream	+
chr1	125179592	125180707	MACS2_peak_296	280	2.6774	32.74374	28.01155	568	EMBP1	NR_003955	Downstream	+
chr1	125181548	125181884	MACS2_peak_297	135	2.18728	17.90611	13.53977	278	EMBP1	NR_003955	Downstream	+
chr1	125182117	125182719	MACS2_peak_298	320	2.88158	36.8747	32.06224	290	EMBP1	NR_003955	Downstream	+
chr1	125182941	125183118	MACS2_peak_299	336	2.96183	38.46171	33.61562	79	EMBP1	NR_003955	Downstream	+
chr1	125183517	125183833	MACS2_peak_300	247	2.71015	29.41397	24.75691	207	EMBP1	NR_003955	Downstream	+
chr1	1382639	1382737	MACS2_peak_11	58	6.07797	9.85614	5.81852	42	AURKAIP1	NM_017900	Upstream	-
chr1	143184629	143185364	MACS2_peak_301	1026	8.38677	108.66656	102.63931	530	LOC645166	NR_027354	Downstream	-
chr1	143185823	143185960	MACS2_peak_302	435	4.86948	48.62252	43.57087	63	LOC645166	NR_027354	Downstream	-
chr1	143186724	143186825	MACS2_peak_303	202	3.28252	24.76749	20.2134	44	LOC645166	NR_027354	Downstream	-
chr1	143187193	143187359	MACS2_peak_304	652	5.24235	70.67546	65.22616	85	LOC645166	NR_027354	Downstream	-
chr1	143187712	143187889	MACS2_peak_305	503	4.33153	55.53446	50.34732	74	LOC645166	NR_027354	Downstream	-
chr1	143190584	143190712	MACS2_peak_306	340	3.16622	38.93406	34.07961	70	LOC645166	NR_027354	Downstream	-
chr1	143191093	143191231	MACS2_peak_307	362	3.27075	41.16502	36.26971	69	LOC645166	NR_027354	Downstream	-
chr1	143191871	143191974	MACS2_peak_308	182	2.61352	22.70624	18.20425	59	LOC645166	NR_027354	Downstream	-
chr1	143192229	143192474	MACS2_peak_309	388	3.49344	43.76937	38.81955	183	LOC645166	NR_027354	Downstream	-
chr1	143192687	143193230	MACS2_peak_310	460	3.8482	51.20258	46.09785	395	LOC645166	NR_027354	Downstream	-
chr1	143193406	143193876	MACS2_peak_311	490	3.95196	54.2007	49.03644	283	LOC645166	NR_027354	Downstream	-
chr1	143194888	143195271	MACS2_peak_312	511	4.0337	56.33971	51.13923	311	LOC645166	NR_027354	Downstream	-
chr1	143195624	143195724	MACS2_peak_313	304	3.23511	35.2723	30.49065	53	LOC645166	NR_027354	Downstream	-
chr1	143200221	143200414	MACS2_peak_314	773	5.94766	82.96333	77.31339	84	LOC645166	NR_027354	Downstream	-
chr1	143200554	143200814	MACS2_peak_315	697	5.77888	75.24336	69.71907	172	LOC645166	NR_027354	Downstream	-
chr1	143201473	143201669	MACS2_peak_316	500	4.56936	55.23957	50.05752	96	LOC645166	NR_027354	Downstream	-
chr1	143202451	143202873	MACS2_peak_317	656	4.92606	71.0707	65.61383	245	LOC645166	NR_027354	Downstream	-
chr1	143203321	143203585	MACS2_peak_318	572	4.60744	62.53143	57.22213	98	LOC645166	NR_027354	Downstream	-
chr1	143206063	143206812	MACS2_peak_319	669	5.34909	72.40073	66.92248	71	LOC645166	NR_027354	Downstream	-
chr1	143206941	143207209	MACS2_peak_320	661	5.47244	71.63802	66.17213	184	LOC645166	NR_027354	Downstream	-
chr1	143211791	143211906	MACS2_peak_321	339	3.20812	38.82669	33.9737	52	LOC645166	NR_027354	Downstream	-
chr1	143212462	143212760	MACS2_peak_322	354	3.17754	40.36432	35.48315	82	LOC645166	NR_027354	Downstream	-
chr1	143212862	143213340	MACS2_peak_323	324	2.93054	37.29975	32.4771	390	LOC645166	NR_027354	Downstream	-
chr1	143214247	143214559	MACS2_peak_324	249	2.56781	29.59999	24.93898	216	LOC645166	NR_027354	Downstream	-
chr1	143214661	143214908	MACS2_peak_325	249	2.53896	29.58399	24.92331	111	LOC645166	NR_027354	Downstream	-
chr1	143215058	143215403	MACS2_peak_326	168	2.222	21.34299	16.8775	132	LOC645166	NR_027354	Downstream	-
chr1	143215638	143215841	MACS2_peak_327	127	2.03272	17.07934	12.73813	126	LOC645166	NR_027354	Downstream	-
chr1	143216644	143216862	MACS2_peak_328	153	2.08224	19.74629	15.32396	174	LOC645166	NR_027354	Downstream	-
chr1	143217304	143217628	MACS2_peak_329	146	2.04883	19.0224	14.62215	270	LOC645166	NR_027354	Downstream	-
chr1	143217763	143217938	MACS2_peak_330	91	1.85294	13.32245	9.11563	140	LOC645166	NR_027354	Downstream	-
chr1	143218347	143218459	MACS2_peak_331	140	2.0545	18.4184	14.03533	43	LOC645166	NR_027354	Downstream	-
chr1	143218805	143218926	MACS2_peak_332	126	2.00644	16.98893	12.65077	34	LOC645166	NR_027354	Downstream	-
chr1	143219679	143219959	MACS2_peak_333	211	2.37131	25.68857	21.11642	109	LOC645166	NR_027354	Downstream	-
chr1	143220293	143220431	MACS2_peak_334	215	2.45652	26.12167	21.53753	59	LOC645166	NR_027354	Downstream	-
chr1	143220813	143221105	MACS2_peak_335	263	2.68565	31.02282	26.33144	239	LOC645166	NR_027354	Downstream	-
chr1	143221445	143221651	MACS2_peak_336	248	2.66205	29.47998	24.82178	47	LOC645166	NR_027354	Downstream	-
chr1	143222140	143222316	MACS2_peak_337	77	2.00692	11.92628	7.78391	36	LOC645166	NR_027354	Downstream	-
chr1	143222421	143222553	MACS2_peak_338	362	3.21959	41.11529	36.22087	62	LOC645166	NR_027354	Downstream	-
chr1	143222943	143223055	MACS2_peak_339	386	3.37398	43.60411	38.65775	57	LOC645166	NR_027354	Downstream	-
chr1	143223327	143223438	MACS2_peak_340	142	2.43678	18.60265	14.21456	64	LOC645166	NR_027354	Downstream	-
chr1	143227369	143227628	MACS2_peak_341	296	3.6973	34.39954	29.63378	214	LOC645166	NR_027354	Downstream	-
chr1	143227736	143228038	MACS2_peak_342	391	4.0672	44.14418	39.18647	233	LOC645166	NR_027354	Downstream	-
chr1	143230263	143230601	MACS2_peak_343	609	4.71333	66.33247	60.95665	70	LOC645166	NR_027354	Downstream	-
chr1	143231264	143231581	MACS2_peak_344	536	4.21306	58.8785	53.63619	144	LOC645166	NR_027354	Downstream	-
chr1	143232388	143232515	MACS2_peak_345	391	3.54657	44.148	39.19027	65	LOC645166	NR_027354	Downstream	-
chr1	143232755	143233320	MACS2_peak_346	486	3.91352	53.85431	48.69675	212	LOC645166	NR_027354	Downstream	-
chr1	143233505	143233643	MACS2_peak_347	362	3.38633	41.11946	36.22501	55	LOC645166	NR_027354	Downstream	-
chr1	143235721	143235865	MACS2_peak_348	291	2.90285	33.89227	29.13754	73	LOC645166	NR_027354	Downstream	-
chr1	143237393	143237543	MACS2_peak_349	340	3.16186	38.85818	34.0047	98	LOC645166	NR_027354	Downstream	-
chr1	143238027	143238247	MACS2_peak_350	343	3.36685	39.24324	34.38366	159	LOC645166	NR_027354	Downstream	-
chr1	143238707	143238960	MACS2_peak_351	246	3.07469	29.29362	24.63938	48	LOC645166	NR_027354	Downstream	-
chr1	143239269	143239887	MACS2_peak_352	582	4.39673	63.53439	58.20752	254	LOC645166	NR_027354	Downstream	-
chr1	143240085	143240592	MACS2_peak_353	454	3.91025	50.56096	45.46736	217	LOC645166	NR_027354	Downstream	-
chr1	143240765	143240970	MACS2_peak_354	99	2.31658	14.15255	9.91619	152	LOC645166	NR_027354	Downstream	-
chr1	143245453	143245575	MACS2_peak_355	409	4.42819	45.97583	40.9778	63	LOC645166	NR_027354	Downstream	-
chr1	143246843	143247438	MACS2_peak_356	745	5.62878	80.19749	74.59303	508	LOC645166	NR_027354	Downstream	-
chr1	143249734	143250044	MACS2_peak_357	461	3.73866	51.23985	46.13424	107	LOC645166	NR_027354	Downstream	-
chr1	143251610	143251775	MACS2_peak_358	363	3.10263	41.19606	36.30028	58	LOC645166	NR_027354	Downstream	-
chr1	143251907	143252259	MACS2_peak_359	331	2.97759	38.02504	33.18806	70	LOC645166	NR_027354	Downstream	-
chr1	143252964	143253212	MACS2_peak_360	393	3.26807	44.32797	39.36515	117	LOC645166	NR_027354	Downstream	-
chr1	143253737	143253865	MACS2_peak_361	380	3.22986	42.98112	38.04778	58	LOC645166	NR_027354	Downstream	-
chr1	143254438	143254949	MACS2_peak_362	412	3.37337	46.27628	41.27085	408	LOC645166	NR_027354	Downstream	-
chr1	143255289	143255395	MACS2_peak_363	145	2.43572	18.98437	14.58528	55	LOC645166	NR_027354	Downstream	-
chr1	143255519	143256107	MACS2_peak_364	442	3.61137	49.33125	44.26555	392	LOC645166	NR_027354	Downstream	-
chr1	143256399	143256551	MACS2_peak_365	469	3.69724	52.05656	46.93594	59	LOC645166	NR_027354	Downstream	-
chr1	143256768	143256934	MACS2_peak_366	483	3.84193	53.48213	48.3326	80	LOC645166	NR_027354	Downstream	-
chr1	143262107	143263116	MACS2_peak_367	464	3.77411	51.57277	46.46038	65	LOC645166	NR_027354	Downstream	-
chr1	143263355	143263488	MACS2_peak_368	363	3.32475	41.21716	36.32113	63	LOC645166	NR_027354	Downstream	-
chr1	143263622	143263781	MACS2_peak_369	427	3.54695	47.74012	42.70646	94	LOC645166	NR_027354	Downstream	-
chr1	143264014	143264167	MACS2_peak_370	465	3.72101	51.70626	46.59219	91	LOC645166	NR_027354	Downstream	-
chr1	143264458	143264782	MACS2_peak_371	476	3.75725	52.83642	47.69999	183	LOC645166	NR_027354	Downstream	-
chr1	143265824	143266016	MACS2_peak_372	193	2.67031	23.85599	19.32474	58	LOC645166	NR_027354	Downstream	-
chr1	143266490	143267537	MACS2_peak_373	360	3.33439	40.93838	36.04669	361	LOC645166	NR_027354	Downstream	-
chr1	143275700	143275925	MACS2_peak_374	956	17.9963	101.61275	95.68008	85	LOC645166	NR_027354	Downstream	-
chr1	144103481	144103794	MACS2_peak_375	200	6.61071	24.61362	20.06276	191	SRGAP2D	NR_120535	Downstream	+
chr1	144104042	144104686	MACS2_peak_376	229	7.11196	27.5528	22.93676	266	SRGAP2D	NR_120535	Downstream	+
chr1	149023643	149023741	MACS2_peak_377	50	5.57947	9.04767	5.05072	40	NBPF9	NM_001277444	Downstream	-
chr1	152216156	152216254	MACS2_peak_378	31	3.92428	7.02437	3.17141	37	HRNR	NM_001009931	Within	-
chr1	163552184	163552282	MACS2_peak_379	69	6.613	11.0835	6.97562	42	LOC100422212	NR_104294	Upstream	-
chr1	168348962	168349066	MACS2_peak_380	113	8.37647	15.64658	11.35266	41	MIR557	NR_030284	Upstream	+
chr1	182348147	182348245	MACS2_peak_381	39	5.2544	7.89112	3.97969	65	GLUL	NM_001033056	Downstream	-
chr1	202205324	202205468	MACS2_peak_382	100	6.80188	14.29544	10.05441	74	PTPRVP	NR_002930	Downstream	+
chr1	202624927	202625048	MACS2_peak_383	19	3.83803	5.59522	1.90768	70	SYT2	NM_001136504	Within	-
chr1	202775369	202775469	MACS2_peak_384	69	6.613	11.0835	6.97562	17	KDM5B	NM_001314042	Within	-
chr1	206924116	206924375	MACS2_peak_385	30	4.46582	6.90478	3.07645	90	FCMR	NM_001142473	Upstream	-
chr1	210653885	210654070	MACS2_peak_386	273	12.61807	32.05101	27.33717	89	HHAT	NM_001170580	Within	+
chr1	2124199	2124329	MACS2_peak_12	75	6.74536	11.68244	7.54949	68	PRKCZ	NM_001033581	Within	+
chr1	224013780	224013879	MACS2_peak_387	27	4.06953	6.50008	2.70359	13	LOC100287497	NR_136593	Within	+
chr1	228557120	228557225	MACS2_peak_388	99	7.78143	14.21572	9.977	33	BTNL10	NM_001287262	Upstream	-
chr1	228558011	228558135	MACS2_peak_389	49	5.73127	8.96241	4.96972	51	BTNL10	NM_001287262	Upstream	-
chr1	228644910	228645008	MACS2_peak_390	34	3.83885	7.37186	3.49596	73	MIR7641-2	NR_106988	Downstream	-
chr1	23392551	23392661	MACS2_peak_23	130	8.89002	17.4532	13.09996	50	TCEA3	NM_003196	Within	-
chr1	236097119	236097396	MACS2_peak_391	604	18.00524	65.77343	60.40646	185	NID1	NM_002508	Upstream	-

chr1	236713828	236713926	MACS2_peak_392	32	4.42226	7.11537	3.25636	90	ACTN2	NM_001278343	Within	+
chr1	236714081	236714827	MACS2_peak_393	65	5.65078	10.59118	6.51141	697	ACTN2	NM_001278343	Within	+
chr1	240184932	240185127	MACS2_peak_394	367	16.06684	41.61972	36.71473	89	FMN2	NM_020066	Within	+
chr1	246024956	246025063	MACS2_peak_395	91	7.25865	13.35029	9.14204	34	SMYD3	NM_022743	Within	-
chr1	248945757	248946097	MACS2_peak_396	113	8.37647	15.64658	11.35266	271	PGBD2	NM_001017434	Downstream	+
chr1	2652183	2652473	MACS2_peak_13	163	7.46572	20.75447	16.30343	82	MMEL1	NM_033467	Upstream	-
chr1	2653901	2654196	MACS2_peak_14	109	5.9893	15.19442	10.91866	256	MMEL1	NM_033467	Upstream	-
chr1	2684023	2684203	MACS2_peak_15	140	6.64977	18.42507	14.04186	86	TTC34	NM_001242672	Within	-
chr1	2697648	2697870	MACS2_peak_16	105	7.08079	14.80366	10.54045	156	TTC34	NM_001242672	Within	-
chr1	2748336	2748501	MACS2_peak_17	31	4.21609	7.03457	3.18109	51	TTC34	NM_001242672	Within	-
chr1	2748972	2749083	MACS2_peak_18	119	6.89034	16.24489	11.93321	60	TTC34	NM_001242672	Within	-
chr1	2758712	2758819	MACS2_peak_19	80	5.88631	12.16979	8.01792	43	TTC34	NM_001242672	Within	-
chr1	2759535	2759645	MACS2_peak_20	135	7.73395	17.8874	13.52173	70	TTC34	NM_001242672	Within	-
chr1	2849318	2849429	MACS2_peak_21	58	6.13013	9.94037	5.89853	63	TTC34	NM_001242672	Upstream	-
chr1	31431804	31432130	MACS2_peak_24	168	9.64606	21.35275	16.88683	226	SERINC2	NM_001199039	Within	+
chr1	5668385	5668528	MACS2_peak_22	33	4.6214	7.16287	3.30054	126	MIR4417	NR_039612	Downstream	+
chr1	629308	629442	MACS2_peak_2	370	5.30867	41.99562	37.08259	64	LOC101928626	NR_125957	Upstream	-
chr1	629652	630002	MACS2_peak_3	450	5.82644	50.16528	45.08242	74	LOC101928626	NR_125957	Upstream	-
chr1	630350	630454	MACS2_peak_4	240	4.31432	28.65878	24.01752	53	MIR6723	NR_106781	Downstream	-
chr1	630792	631410	MACS2_peak_5	676	7.18088	73.159	67.66682	542	MIR6723	NR_106781	Downstream	-
chr1	631893	632286	MACS2_peak_6	701	7.32072	75.72824	70.1982	316	MIR6723	NR_106781	Downstream	-
chr1	632520	632646	MACS2_peak_7	321	4.91874	36.95415	32.13792	67	MIR6723	NR_106781	Upstream	-
chr1	633413	633513	MACS2_peak_8	214	4.11568	26.06888	21.48565	55	MIR6723	NR_106781	Upstream	-
chr1	633922	634099	MACS2_peak_9	781	7.76436	83.78383	78.11775	88	MIR6723	NR_106781	Upstream	-
chr1	67159065	67159163	MACS2_peak_25	49	5.73127	8.96241	4.96972	52	C1orf141	NM_001276351	Upstream	-
chr1	91387255	91387528	MACS2_peak_26	291	12.88391	33.85614	29.1022	188	HFM1	NM_001017975	Within	-
chr2	109199347	109199844	MACS2_peak_1482	444	14.03861	49.49715	44.42813	137	MIR4265	NR_036223	Upstream	-
chr2	10953734	10953851	MACS2_peak_1437	33	4.8583	7.25931	3.3907	92	KCNF1	NM_002236	Downstream	+
chr2	115439030	115439165	MACS2_peak_1483	22	4.40867	6.02587	2.26765	59	DPP10	NM_001178037	Within	+
chr2	132270789	132270888	MACS2_peak_1484	91	7.49474	13.31573	9.10899	63	MIR663B	NR_031608	Upstream	-
chr2	132394071	132394238	MACS2_peak_1485	69	6.613	11.0835	6.97562	71	FAM201B	NR_135203	Downstream	+
chr2	135117445	135117699	MACS2_peak_1486	161	10.13994	20.56598	16.11957	61	RAB3GAP1	NM_001172435	Within	+
chr2	147369671	147369784	MACS2_peak_1487	225	12.34427	27.12572	22.51836	71	ACVR2A	NM_001278580	Upstream	+
chr2	148881730	148881849	MACS2_peak_1488	573	22.16089	62.61768	57.30709	37	KIF5C	NM_004522	Within	+
chr2	161281233	161281352	MACS2_peak_1489	105	7.08079	14.80366	10.54045	54	LINC01806	NR_110163	Downstream	+
chr2	161281743	161281871	MACS2_peak_1490	145	8.31574	18.97384	14.57494	56	LINC01806	NR_110163	Downstream	+
chr2	1861773	1861934	MACS2_peak_1430	238	12.44194	28.52868	23.89027	81	PXDN	NM_012293	Upstream	-
chr2	203987066	203987180	MACS2_peak_1491	125	8.81734	16.84579	12.51139	73	ICOS	NM_012092	Downstream	+
chr2	239644548	239644934	MACS2_peak_1492	165	9.7725	21.02501	16.567	64	LOC150935	NR_037808	Upstream	+
chr2	241590889	241591014	MACS2_peak_1493	32	4.7824	7.13774	3.27727	97	THAP4	NM_001164356	Within	-
chr2	242183423	242183535	MACS2_peak_1494	219	12.00274	26.54915	21.95715	26	LOC728323	NR_130699	Downstream	+
chr2	2721390	2721488	MACS2_peak_1431	40	5.2904	7.94841	4.02217	19	LINC01250	NR_110228	Downstream	-
chr2	3180646	3181168	MACS2_peak_1432	156	8.90167	20.03731	15.60769	448	TSSC1	NM_001330530	Downstream	-
chr2	3221217	3221368	MACS2_peak_1433	113	8.09717	15.64679	11.35275	91	TSSC1	NM_001330530	Within	-
chr2	32916243	32916632	MACS2_peak_1438	218	11.55747	26.39594	21.80675	242	LINC00486	NR_027099	Within	+
chr2	3331919	3332017	MACS2_peak_1434	42	5.02946	8.14235	4.20526	69	TSSC1	NM_001330530	Within	-
chr2	5022665	5022801	MACS2_peak_1435	69	6.613	11.0835	6.97562	59	LINC01249	NR_034134	Upstream	-
chr2	64375742	64376186	MACS2_peak_1439	151	9.24001	19.57592	15.15829	376	MIR4433A	NR_039632	Downstream	+
chr2	7488694	7488986	MACS2_peak_1436	148	9.69907	19.30642	14.89557	213	LOC100506274	NR_038432	Downstream	+
chr2	87398367	87398493	MACS2_peak_1440	104	6.58256	14.75134	10.49005	54	CYTOR	NR_024206	Upstream	+
chr2	87428552	87428691	MACS2_peak_1441	74	4.6146	11.54431	7.41701	79	CYTOR	NR_024206	Upstream	+
chr2	89822495	89822594	MACS2_peak_1442	78	4.94015	12.01032	7.86475	42	LOC101927050	NR_136329	Upstream	-
chr2	89826412	89826540	MACS2_peak_1443	326	6.28171	37.45565	32.62902	70	LOC101927050	NR_136329	Upstream	-
chr2	89827984	89828083	MACS2_peak_1444	151	4.18543	19.51767	15.1016	43	LOC101927050	NR_136329	Upstream	-
chr2	89828586	89828717	MACS2_peak_1445	461	6.6742	51.24278	46.13704	66	LOC101927050	NR_136329	Upstream	-
chr2	89829177	89829297	MACS2_peak_1446	364	5.85414	41.38462	36.48409	67	LOC101927050	NR_136329	Upstream	-
chr2	89829428	89829546	MACS2_peak_1447	301	5.32101	34.96552	30.18933	59	LOC101927050	NR_136329	Upstream	-
chr2	89830930	89831153	MACS2_peak_1448	797	8.71352	85.48158	79.78845	82	LOC101927050	NR_136329	Upstream	-
chr2	89833265	89833414	MACS2_peak_1449	548	6.82513	60.07074	54.80592	60	LOC101927050	NR_136329	Upstream	-
chr2	89836278	89836578	MACS2_peak_1450	226	4.63249	27.27821	22.6685	231	LOC101927050	NR_136329	Upstream	-
chr2	89838020	89838156	MACS2_peak_1451	456	6.90379	50.77978	45.68259	71	LOC101927050	NR_136329	Upstream	-
chr2	89838615	89838717	MACS2_peak_1452	74	3.34422	11.61341	7.48346	33	LOC101927050	NR_136329	Upstream	-
chr2	89839641	89839750	MACS2_peak_1453	228	5.3102	27.41592	22.80287	52	LOC101927050	NR_136329	Upstream	-
chr2	89840269	89840595	MACS2_peak_1454	783	10.44609	83.99078	78.3215	77	LOC101927050	NR_136329	Upstream	-
chr2	89841016	89841178	MACS2_peak_1455	915	11.66141	97.3705	91.50064	86	LOC101927050	NR_136329	Upstream	-
chr2	90288842	90288940	MACS2_peak_1456	24	4.08112	6.25672	2.48042	36	LOC101927050	NR_136329	Upstream	-
chr2	90291861	90292003	MACS2_peak_1457	41	4.61148	8.04363	4.11222	49	LOC101927050	NR_136329	Upstream	-
chr2	90292243	90292361	MACS2_peak_1458	49	4.91358	8.91079	4.92951	33	LOC101927050	NR_136329	Upstream	-
chr2	90380697	90380897	MACS2_peak_1459	174	5.88666	21.88544	17.40217	58	LOC101927050	NR_136329	Upstream	-
chr2	90381045	90381197	MACS2_peak_1460	263	6.89754	31.08526	26.39286	72	LOC101927050	NR_136329	Upstream	-
chr2	90381630	90381880	MACS2_peak_1461	76	3.88604	11.76245	7.62639	38	LOC101927050	NR_136329	Upstream	-
chr2	90382573	90382760	MACS2_peak_1462	118	4.53976	16.14471	11.83561	65	LOC101927050	NR_136329	Upstream	-
chr2	90383745	90383852	MACS2_peak_1463	78	3.67446	12.03354	7.88694	58	LOC101927050	NR_136329	Upstream	-
chr2	90384412	90384632	MACS2_peak_1464	101	4.00328	14.39344	10.14875	51	LOC101927050	NR_136329	Upstream	-
chr2	90385871	90385993	MACS2_peak_1465	137	4.26523	18.12225	13.74663	55	LOC101927050	NR_136329	Upstream	-
chr2	90390528	90390796	MACS2_peak_1466	161	4.82357	20.56748	16.12106	207	LOC101927050	NR_136329	Upstream	-
chr2	90392102	90392201	MACS2_peak_1467	102	4.04014	14.54093	10.28641	56	LOC101927050	NR_136329	Upstream	-
chr2	90395536	90395742	MACS2_peak_1468	86	3.51584	12.84112	8.65749	61	LOC101927050	NR_136329	Upstream	-
chr2	90397457	90397916	MACS2_peak_1469	141	4.3527	18.51823	14.13251	331	LOC101927050	NR_136329	Upstream	-
chr2	90398050	90398337	MACS2_peak_1470	110	3.95363	15.29401	11.01509	33	LOC101927050	NR_136329	Upstream	-
chr2	90398448	90398748	MACS2_peak_1471	147	4.48516	19.10842	14.70638	73	LOC101927050	NR_136329	Upstream	-
chr2	90400996	90401257	MACS2_peak_1472	53	3.46075	9.32393	5.31283	178	LOC101927050	NR_136329	Upstream	-
chr2	909774	909916	MACS2_peak_1428	115	8.19471	15.81014	11.51105	67	LOC101060385	NR_110179	Upstream	-
chr2	910034	910132	MACS2_peak_1429	35	4.95663	7.41652	3.53681	66	LOC101060385	NR_110179	Upstream	-
chr2	91421793	91422106	MACS2_peak_1473	295	10.0115	34.30682	29.54247	191	LOC654342	NR_027238	Downstream	-
chr2	91422267	91422414	MACS2_peak_1474	261	9.27566	30.78804	26.10051	79	LOC654342	NR_027238	Downstream	-
chr2	92270838	92270950	MACS2_peak_1475	136	9.2582	18.06606	13.69225	61	ACTR3BP2	NR_027714	Downstream	+
chr2	92792626	92792724	MACS2_peak_1476	59	6.17214	10.00816	5.95538	37	ACTR3BP2	NR_027714	Downstream	+
chr2	92994374	92994472	MACS2_peak_1477	80	7.05387	12.18657	8.02795	38	ACTR3BP2	NR_027714	Downstream	+
chr2	93697241	93697339	MACS2_peak_1478	22	4.40867	6.02587	2.26765	47	LOC100133920	NR_024443	Downstream	-
chr2	93825332	93825485	MACS2_peak_1479	80	7.05387	12.18657	8.02795	60	LOC100133920	NR_024443	Downstream	-
chr2	93994100	93994230	MACS2_peak_1480	91	7.49474	13.31573	9.10899	86	LOC100133920	NR_024443	Downstream	-
chr2	94502413	94502521	MACS2_peak_1481	105	7.61383	14.83107	10.56682	55	LOC100133920	NR_024443	Downstream	-
chr3	195483656	195483788	MACS2_peak_1854	149	8.27256	19.40301	14.98966	67	PPP1R2	NM_001291504	Downstream	-
chr3	195504114	195504212	MACS2_peak_1855	21	3.98804	5.84515	2.13091	52	PPP1R2	NM_001291504	Downstream	-
chr3	196898764	196898901	MACS2_peak_1856	507	20.75047	55.97255	50.77765	62	SENP5	NM_001308045	Within	+
chr3	197459816	197459922	MACS2_peak_1857	102	7.9356	14.4695	10.21727	35	BDH1	NM_004051	Downstream	-
chr3	198100983	198101087	MACS2_peak_1858	80	5.3234	12.16853	8.01675	46	ANKRD18DP	NR_003291	Upstream	-
chr3	262289	262466	MACS2_peak_1811	77	6.6453	11.91407	7.77209	83	CHL1	NR_045572	Downstream	+
chr3	38084438	38084571	MACS2_peak_1812	121	7.77027	16.50496	12.18484	58	DLEC1	NM_007335	Within	+
chr3	64696464	64696579	MACS2_peak_1813	230	11.58532	27.6686	23.05031	61	ADAMTS9	NM_182920	Upstream	-
chr3	75668869	75669010	MACS2_peak_1814	206	7.11491	25.17707	20.61507	66	FRG2C	NM_001124759	Downstream	+
chr3	75669122	75669615	MACS2_peak_1815	1353	22.44107	141.76746	135.35916	178	FRG2C	NM_001124759	Downstream	+
chr3	90489344	90489451	MACS2_peak_1816	166	10.16002	21.13824	16.6772	57	EPHA3	NM_005233	Downstream	+
chr3	90490236	90490342	MACS2_peak_1817	144	9.43073	18.85891	14.46339	64	EPHA3	NM_005233	Downstream	+
chr3	90523346	90523453	MACS2_peak_1818	67	6.23053	10.83427	6.74392	82	EPHA3	NM_005233	Downstream	+
chr3	90538715	90538819	MACS2_peak_1819	107	7.72917	15.02678	10.75658	52	EPHA3	NM_005233	Downstream	+
chr3	90542691	90542815	MACS2_peak_1820	130	8.29187	17.4248	13.07241	42	EPHA3	NM_005233	Downstream	+
chr3	90583041	90583180	MACS2_peak_1821	48	5.2591	8.85258	4.87431	79	EPHA3	NM_005233	Downstream	+
chr3	90610216	90610315	MACS2_peak_1822	85	7.13746	12.72907	8.5497	43	EPHA3	NM_005233	Downstream	+
chr3	90654972	90655097	MACS2_peak_1823	143	9.35215	18.72755	14.33535	73	EPHA3	NM_005233	Downstream	+
chr3	90673628	90673745	MACS2_peak_1824	139	8.82172	18.34087	13.95983	65	EPHA3	NM_005233	Downstream	+
chr3	90824997	90825095	MACS2_peak_1825	49	5.73127	8.96241	4.96972	59	EPHA3	NM_005233	Downstream	+
chr3	90846191	90846565	MACS2_peak_1826	31	4.84953	6.96861	3.11953	227	EPHA3	NM_005233	Downstream	+
chr3	90876117	90876215	MACS2_peak_1827	49	5.73127	8.96241	4.96972	39	EPHA3	NM_005233	Downstream	+
chr3	90883040	90883147	MACS2_peak_1828	64	6.29776	10.57093	6.49192	37	EPHA3	NM_005233	Downstream	+
chr3	90908029	90908209	MACS2_peak_1829	194	11.19242	23.99786	19.46297	97	EPHA3	NM_005233	Downstream	+
chr3	90914316	90914473	MACS2_peak_1830	47	5.5727	8.70803	4.73749	84	EPHA3	NM_005233	Downstream	+
chr3	91006891	91007003	MACS2_peak_1831	80	7.03606	12.15752	8.00598	49	EPHA3	NM_005233	Downstream	+
chr3	91007612	91007720	MACS2_peak_1832	69	6.613	11.0835	6.97562	36	EPHA3	NM_005233	Downstream	+
chr3	91182721	91182873	MACS2_peak_1833	99	7.7486	14.16161	9.92496	72	EPHA3	NM_005233	Downstream	+
chr3	91232602	91232702	MACS2_peak_1834	91	7.49474	13.31573	9.10899	52	EPHA3	NM_005233	Downstream	+
chr3	91262046	91262144	MACS2_peak_1835	59	6.17214	10.00816	5.95538	34	EPHA3	NM_005233	Downstream	+
chr3	91282809	91282907	MACS2_peak_1836	85	6.39672	12.70986	8.53171	55	EPHA3	NM_005233	Downstream	+
chr3	91283996	91284205	MACS2_peak_1837	31	4.49609	6.95517	3.11953	186	EPHA3	NM_005233	Downstream	+
chr3	91550221	91550371	MACS2_peak_1838	93	5.59669	13.52026	9.30613	87	EPHA3	NM_005233	Downstream	+
chr3	91550507	91550924	MACS2_peak_1839	149	7.06714	19.38252	14.9698	54	EPHA3	NM_005233	Downstream	+
chr3	91553033	91553182	MACS2_peak_1840	24	3.64398	6.21319	2.44038	59	EPHA3	NM_005233	Downstream	+
chr3	91572768	91572915	MACS2_peak_1841	364	16.75294	41.33307	36.43348	80	EPHA3	NM_005233	Downstream	+

chr3	91648534	91648782	MACS2_peak_1842	80	7.05387	12.18657	8.02795	185	PROS1	NM_001314077	Downstream	-
chr3	91891214	91891499	MACS2_peak_1843	320	14.99125	36.88617	32.07345	195	PROS1	NM_001314077	Downstream	-
chr3	92036088	92036186	MACS2_peak_1844	59	6.17214	10.00816	5.95538	25	PROS1	NM_001314077	Downstream	-
chr3	92153183	92153281	MACS2_peak_1845	80	7.05387	12.18657	8.02795	38	PROS1	NM_001314077	Downstream	-
chr3	92279190	92279350	MACS2_peak_1846	505	19.6959	55.78169	50.59005	79	PROS1	NM_001314077	Downstream	-
chr3	92590038	92590191	MACS2_peak_1847	453	19.39814	50.42184	45.33138	84	PROS1	NM_001314077	Downstream	-
chr3	93090476	93090583	MACS2_peak_1848	80	7.05387	12.18657	8.02795	59	PROS1	NM_001314077	Downstream	-
chr3	93381818	93381918	MACS2_peak_1849	80	7.05387	12.18657	8.02795	38	PROS1	NM_001314077	Downstream	-
chr3	93404853	93404951	MACS2_peak_1850	31	4.84953	6.96861	3.11953	27	PROS1	NM_001314077	Downstream	-
chr3	93470358	93470804	MACS2_peak_1851	1862	21.84907	193.37982	186.207	256	PROS1	NM_001314077	Downstream	-
chr3	93706480	93706665	MACS2_peak_1852	36	4.17957	7.52791	3.64128	43	PROS1	NM_001314077	Downstream	-
chr3	93712288	93712576	MACS2_peak_1853	152	6.97114	19.62893	15.2098	207	PROS1	NM_001314077	Downstream	-
chr3_KI270782v1_alt	105149	105280	MACS2_peak_1859	59	6.17214	10.00816	5.95538	68	BDH1	NM_203315	Downstream	+
chr4	109624896	109624998	MACS2_peak_1961	69	6.613	11.0835	6.97562	35	MCUB	NM_017918	Within	+
chr4	1434997	1435562	MACS2_peak_1861	378	17.19381	42.82129	37.89103	446	NKX1-1	NM_001290079	Upstream	-
chr4	1441746	1441918	MACS2_peak_1862	49	5.73127	8.96241	4.96972	84	NKX1-1	NM_001290079	Upstream	-
chr4	146286138	146286488	MACS2_peak_1962	96	7.03631	13.83889	9.61434	250	SLC10A7	NM_001317816	Within	-
chr4	146286728	146286841	MACS2_peak_1963	76	6.31092	11.74806	7.61238	48	SLC10A7	NM_001317816	Within	-
chr4	166580279	166580441	MACS2_peak_1964	69	6.613	11.0835	6.97562	114	SPOCK3	NM_001204353	Downstream	-
chr4	181239232	181239417	MACS2_peak_1965	38	4.16455	7.78096	3.87703	30	LINC00290	NR_033918	Upstream	-
chr4	181240047	181240390	MACS2_peak_1966	39	4.2123	7.87716	3.967	19	LINC00290	NR_033918	Upstream	-
chr4	181241289	181241387	MACS2_peak_1967	46	4.44632	8.6453	4.67832	37	LINC00290	NR_033918	Upstream	-
chr4	186432745	186432879	MACS2_peak_1968	104	6.78226	14.69744	10.43772	61	F11-AS1	NR_033901	Within	-
chr4	186433701	186433840	MACS2_peak_1969	134	7.68912	17.80225	13.43879	70	F11-AS1	NR_033901	Within	-
chr4	186434112	186434249	MACS2_peak_1970	175	8.84643	22.0689	17.58153	69	F11-AS1	NR_033901	Within	-
chr4	186436365	186436465	MACS2_peak_1971	108	7.0259	15.15245	10.87828	58	F11-AS1	NR_033901	Within	-
chr4	188631212	188631340	MACS2_peak_1972	49	5.73127	8.96241	4.96972	69	LINC01060	NR_033869	Downstream	+
chr4	189036123	189036302	MACS2_peak_1973	140	8.85501	18.3979	14.01548	88	LINC01060	NR_033869	Downstream	+
chr4	189308957	189309070	MACS2_peak_1974	38	5.16573	7.74996	3.84768	42	LINC01262	NR_121679	Upstream	+
chr4	189881273	189881432	MACS2_peak_1975	80	7.05387	12.18657	8.02795	34	LINC01596	NR_132380	Upstream	+
chr4	190021667	190021781	MACS2_peak_1976	189	7.57274	23.46406	18.94124	62	FRG2	NM_001286820	Downstream	-
chr4	190022035	190022751	MACS2_peak_1977	824	18.29826	88.15855	82.43139	445	FRG2	NM_001286820	Downstream	-
chr4	190122838	190123083	MACS2_peak_1978	265	13.66687	31.24884	26.55088	189	DUX4	NM_001293798	Upstream	+
chr4	190177621	190178501	MACS2_peak_1979	941	8.32683	100.02699	94.11623	798	DUX4	NR_137167	Within	+
chr4	190178743	190178872	MACS2_peak_1980	472	5.74204	52.33414	47.20634	62	DUX4	NR_137167	Within	+
chr4	190179250	190180000	MACS2_peak_1981	1056	8.91602	111.72755	105.6666	446	DUX4	NR_137167	Within	+
chr4	190181009	190181166	MACS2_peak_1982	662	6.88489	71.71381	66.24647	72	DUX4	NR_137167	Within	+
chr4	26244260	26244394	MACS2_peak_1872	136	9.2582	18.06606	13.69225	88	RBPJ	NM_005349	Upstream	+
chr4	26646000	26646121	MACS2_peak_1873	325	14.86182	37.36473	32.54045	71	TBC1D19	NM_001292054	Within	+
chr4	3814932	3815090	MACS2_peak_1863	134	7.97092	17.8393	13.47473	95	ADRA2C	NM_000683	Downstream	+
chr4	40294643	40294759	MACS2_peak_1874	115	7.1599	15.85694	11.5567	28	LOC101060498	NR_121641	Upstream	+
chr4	40295192	40295564	MACS2_peak_1875	60	5.38567	10.1136	6.05566	201	LOC101060498	NR_121641	Upstream	+
chr4	49091359	49091470	MACS2_peak_1876	236	4.63001	28.24419	23.61316	41	CWH43	NM_001286791	Downstream	+
chr4	49091778	49092439	MACS2_peak_1877	196	4.088	24.16085	19.62243	143	CWH43	NM_001286791	Downstream	+
chr4	49092751	49092891	MACS2_peak_1878	570	6.09515	62.32	57.01507	74	CWH43	NM_001286791	Downstream	+
chr4	49093284	49093401	MACS2_peak_1879	149	3.281	19.35094	14.93884	51	CWH43	NM_001286791	Downstream	+
chr4	49094902	49095015	MACS2_peak_1880	208	3.30755	25.45791	20.88998	52	CWH43	NM_001286791	Downstream	+
chr4	49096160	49096263	MACS2_peak_1881	260	3.52671	30.76775	26.08076	58	CWH43	NM_001286791	Downstream	+
chr4	49096785	49096900	MACS2_peak_1882	302	3.71106	35.06316	30.28517	45	CWH43	NM_001286791	Downstream	+
chr4	49097152	49097379	MACS2_peak_1883	411	4.2787	46.10645	41.10571	88	CWH43	NM_001286791	Downstream	+
chr4	49097671	49097773	MACS2_peak_1884	114	2.61424	15.77688	11.47894	73	CWH43	NM_001286791	Downstream	+
chr4	49098413	49098839	MACS2_peak_1885	347	3.87422	39.59121	34.72522	179	CWH43	NM_001286791	Downstream	+
chr4	49099644	49099755	MACS2_peak_1886	147	2.76574	19.11851	14.71604	62	CWH43	NM_001286791	Downstream	+
chr4	49102460	49102644	MACS2_peak_1887	330	3.71729	37.93074	33.09525	97	CWH43	NM_001286791	Downstream	+
chr4	49103541	49103651	MACS2_peak_1888	280	3.36854	32.79206	28.05756	57	CWH43	NM_001286791	Downstream	+
chr4	49103884	49103986	MACS2_peak_1889	170	2.83168	21.5661	17.09428	64	CWH43	NM_001286791	Downstream	+
chr4	49104374	49104498	MACS2_peak_1890	382	3.84396	43.1972	38.26024	66	CWH43	NM_001286791	Downstream	+
chr4	49107735	49107898	MACS2_peak_1891	115	2.50074	15.87747	11.57665	74	CWH43	NM_001286791	Downstream	+
chr4	49108264	49108549	MACS2_peak_1892	513	4.40788	56.58891	51.38477	70	CWH43	NM_001286791	Downstream	+
chr4	49108654	49108756	MACS2_peak_1893	129	2.64257	17.29423	12.94615	39	CWH43	NM_001286791	Downstream	+
chr4	49109495	49110065	MACS2_peak_1894	336	3.80683	38.5134	33.66616	456	CWH43	NM_001286791	Downstream	+
chr4	49110685	49110846	MACS2_peak_1895	511	4.68417	56.34058	51.14005	72	CWH43	NM_001286791	Downstream	+
chr4	49118502	49118713	MACS2_peak_1896	66	2.40292	10.76553	6.6779	36	CWH43	NM_001286791	Downstream	+
chr4	49118848	49119091	MACS2_peak_1897	602	5.3808	65.61266	60.2489	111	CWH43	NM_001286791	Downstream	+
chr4	49119508	49119672	MACS2_peak_1898	229	3.51634	27.57246	22.95621	78	CWH43	NM_001286791	Downstream	+
chr4	49119835	49120325	MACS2_peak_1899	416	4.54615	46.62493	41.61362	381	CWH43	NM_001286791	Downstream	+
chr4	49120455	49120631	MACS2_peak_1900	126	2.86216	16.99711	12.65848	131	CWH43	NM_001286791	Downstream	+
chr4	49120908	49121180	MACS2_peak_1901	178	3.2115	22.36548	17.87164	79	CWH43	NM_001286791	Downstream	+
chr4	49123606	49123719	MACS2_peak_1902	256	3.77535	30.35568	25.67736	59	CWH43	NM_001286791	Downstream	+
chr4	49129979	49130189	MACS2_peak_1903	308	5.49789	35.64823	30.85688	162	CWH43	NM_001286791	Downstream	+
chr4	49130632	49130731	MACS2_peak_1904	92	3.39674	13.43889	9.22782	38	CWH43	NM_001286791	Downstream	+
chr4	49130876	49131002	MACS2_peak_1905	140	3.96663	18.38778	14.00565	59	CWH43	NM_001286791	Downstream	+
chr4	49131674	49131804	MACS2_peak_1906	117	3.72049	16.04812	11.74184	61	CWH43	NM_001286791	Downstream	+
chr4	49137047	49137310	MACS2_peak_1907	601	6.74886	65.51512	60.15229	186	CWH43	NM_001286791	Downstream	+
chr4	49138088	49138217	MACS2_peak_1908	165	3.77921	21.03482	16.57667	60	CWH43	NM_001286791	Downstream	+
chr4	49139633	49139808	MACS2_peak_1909	918	8.30716	97.70544	91.83286	89	CWH43	NM_001286791	Downstream	+
chr4	49140537	49140662	MACS2_peak_1910	380	5.10655	43.00504	38.0713	54	CWH43	NM_001286791	Downstream	+
chr4	49140812	49140920	MACS2_peak_1911	251	4.18598	29.78533	25.1202	60	CWH43	NM_001286791	Downstream	+
chr4	49141838	49141954	MACS2_peak_1912	260	4.27055	30.74284	26.05648	59	PIGG	NM_001286791	Downstream	+
chr4	49142089	49142233	MACS2_peak_1913	410	5.34579	46.03229	41.03325	70	CWH43	NM_001286791	Downstream	+
chr4	49144568	49144776	MACS2_peak_1914	329	4.44858	37.75066	32.91846	116	CWH43	NM_001286791	Downstream	+
chr4	49147292	49147562	MACS2_peak_1915	652	6.26131	70.68261	65.23319	69	CWH43	NM_001286791	Downstream	+
chr4	49149150	49149853	MACS2_peak_1916	701	6.03742	75.65714	70.12765	629	CWH43	NM_001286791	Downstream	+
chr4	49150448	49150579	MACS2_peak_1917	378	4.41566	42.73366	37.80662	74	CWH43	NM_001286791	Downstream	+
chr4	49153181	49153295	MACS2_peak_1918	341	4.38639	39.04103	34.18484	38	CWH43	NM_001286791	Downstream	+
chr4	49153499	49153780	MACS2_peak_1919	108	2.84691	15.11062	10.83808	229	CWH43	NM_001286791	Downstream	+
chr4	49154167	49154268	MACS2_peak_1920	196	3.56042	24.15361	19.61539	27	CWH43	NM_001286791	Downstream	+
chr4	49154401	49154587	MACS2_peak_1921	670	6.55415	72.51655	67.0359	81	CWH43	NM_001286791	Downstream	+
chr4	49154854	49154983	MACS2_peak_1922	424	5.6325	47.49357	42.46492	62	CWH43	NM_001286791	Downstream	+
chr4	49155930	49156028	MACS2_peak_1923	104	3.34841	14.75666	10.49529	65	CWH43	NM_001286791	Downstream	+
chr4	49156423	49156546	MACS2_peak_1924	403	6.14991	45.31707	40.33308	71	CWH43	NM_001286791	Downstream	+
chr4	49292418	49292516	MACS2_peak_1925	91	5.53828	13.38988	9.18021	37	CWH43	NM_001286791	Downstream	+
chr4	49297666	49297795	MACS2_peak_1926	90	6.4536	13.22851	9.03037	61	CWH43	NM_001286791	Downstream	+
chr4	49303814	49304024	MACS2_peak_1927	214	9.74435	26.0532	21.4703	130	CWH43	NM_001286791	Downstream	+
chr4	49311891	49311995	MACS2_peak_1928	43	4.27005	8.28427	4.33926	78	CWH43	NM_001286791	Downstream	+
chr4	49316750	49316898	MACS2_peak_1929	179	7.16684	22.4561	17.95998	75	CWH43	NM_001286791	Downstream	+
chr4	49317574	49317701	MACS2_peak_1930	102	5.52044	14.49489	10.24193	58	CWH43	NM_001286791	Downstream	+
chr4	49322786	49322969	MACS2_peak_1931	119	5.76868	16.28312	11.96996	106	CWH43	NM_001286791	Downstream	+
chr4	49513498	49513717	MACS2_peak_1932	56	3.48842	9.65094	5.62272	73	CWH43	NM_001286791	Downstream	+
chr4	49514182	49514351	MACS2_peak_1933	72	3.77115	11.36521	7.24521	118	CWH43	NM_001286791	Downstream	+
chr4	49514849	49515012	MACS2_peak_1934	137	4.79795	18.08624	13.71167	70	CWH43	NM_001286791	Downstream	+
chr4	49570431	49570577	MACS2_peak_1935	21	3.98804	5.84515	2.13091	128	CWH43	NM_001286791	Downstream	+
chr4	49632625	49632772	MACS2_peak_1936	411	7.38142	46.10484	41.10413	71	CWH43	NM_001286791	Downstream	+
chr4	49632882	49633001	MACS2_peak_1937	333	6.52564	38.21513	33.3749	65	CWH43	NM_001286791	Downstream	+
chr4	49633348	49633490	MACS2_peak_1938	239	5.3169	28.57198	23.93267	67	CWH43	NM_001286791	Downstream	+
chr4	49634259	49634412	MACS2_peak_1939	644	8.61264	69.88551	64.4486	74	CWH43	NM_001286791	Downstream	+
chr4	49634751	49634856	MACS2_peak_1940	174	4.42402	21.95562	17.47127	54	CWH43	NM_001286791	Downstream	+
chr4	49635160	49635269	MACS2_peak_1941	145	4.07309	18.94224	14.54409	75	CWH43	NM_001286791	Downstream	+
chr4	49635484	49635601	MACS2_peak_1942	259	5.14591	30.61333	25.92997	52	CWH43	NM_001286791	Downstream	+
chr4	49637072	49637170	MACS2_peak_1943	125	3.63762	16.864	12.52901	42	CWH43	NM_001286791	Downstream	+
chr4	49638059	49638160	MACS2_peak_1944	159	4.06861	20.3401	15.90273	55	CWH43	NM_001286791	Downstream	+
chr4	49639102	49639200	MACS2_peak_1945	157	4.14678	20.19209	15.75862	49	CWH43	NM_001286791	Downstream	+
chr4	49640297	49640395	MACS2_peak_1946	155	4.40224	19.98105	15.55257	53	CWH43	NM_001286791	Downstream	+
chr4	49641032	49641135	MACS2_peak_1947	110	3.89023	15.28581	11.00704	37	CWH43	NM_001286791	Downstream	+
chr4	49646255	49646387	MACS2_peak_1948	267	6.08901	31.40484	26.70349	51	CWH43	NM_001286791	Downstream	+
chr4	49648669	49648779	MACS2_peak_1949	308	6.00221	35.61188	30.82119	58	CWH43	NM_001286791	Downstream	+
chr4	49651366	49651549	MACS2_peak_1950	1222	13.72141	128.5	122.22701	90	CWH43	NM_001286791	Downstream	+
chr4	49652252	49652540	MACS2_peak_1951	152	4.40507	19.68417	15.2636	75	CWH43	NM_001286791	Downstream	+
chr4	49657506	49657775	MACS2_peak_1952	1673	23.14063	174.1162	167.3383	93	CWH43	NM_001286791	Downstream	+
chr4	49709331	49709571	MACS2_peak_1953	1180	10.14604	124.24937	118.01959	90	CWH43	NM_001286791	Downstream	+
chr4	49709674	49710161	MACS2_peak_1954	692	7.45728	74.81573	69.29738	61	CWH43	NM_001286791	Downstream	+
chr4	49710526	49711023	MACS2_peak_1955	380	5.44206	42.97146	38.03833	180	CWH43	NM_001286791	Downstream	+
chr4	49711273	49711932	MACS2_peak_1956	878	8.48154	93.70045	87.88678	290	CWH43	NM_001286791	Downstream	+
chr4	50328458	50328574	MACS2_peak_1957	31	4.84953	6.96861	3.11953	32	CWH43	NM_001286791	Downstream	+
chr4	50699087	50699195	MACS2_peak_1958	59	5.95474	10.00978	5.95673	31	DCUN1D4	NM_001040402	Upstream	+
chr4	51107250	51107548	MACS2_peak_1959	705	20.0759	76.04803	70.51384	95	DCUN1D4	NM_001040402	Upstream	+
chr4	555051	555638	MACS2_peak_1860	227	10.08762	27.31403	22.70335	79	PIGG	NR_144333	Downstream	+
chr4	59519378	59519476	MACS2_peak_1960	69	6.613	11.0835	6.97562	44	LOC105377247	NR_133942	Downstream	+
chr4	6004104	6004271	MACS2_peak_1864	86	6.47794	12.85533	8.67102	82	MIR378D1	NR_039608	Upstream	-

chr4	6879820	6880002	MACS2_peak_1865	166	8.89962	21.09616	16.63651	61	KIAA0232	NM_001100590	Within	+
chr4	6880105	6880309	MACS2_peak_1866	106	7.12574	14.88484	10.61893	147	KIAA0232	NM_001100590	Within	+
chr4	7416581	7416679	MACS2_peak_1867	30	4.43595	6.855	3.0303	17	PSAPL1	NM_001085382	Downstream	-
chr4	7863963	7864150	MACS2_peak_1868	237	11.98021	28.35947	23.72581	73	AFAP1	NM_198595	Within	-
chr4	9234323	9234428	MACS2_peak_1869	86	6.00175	12.78774	8.60586	58	USP17L15	NM_001256894	Upstream	+
chr4	9271824	9271998	MACS2_peak_1870	205	10.13734	25.06318	20.50323	91	USP17L20	NM_001256861	Downstream	+
chr4	9324326	9324454	MACS2_peak_1871	101	7.33999	14.36336	10.11977	59	USP17L25	NM_001242326	Upstream	+
ır4_GL000008v2_randc	1967	3421	MACS2_peak_1984	220	4.6381	26.65675	22.06214	917	ANKRD20A12P	NR_046228	Downstream	-
ır4_GL000008v2_randc	472	628	MACS2_peak_1983	28	2.516	6.69152	2.87984	86	ANKRD20A12P	NR_046228	Downstream	-
chr4_KI270786v1_alt	218288	218457	MACS2_peak_1985	125	8.81734	16.84579	12.51139	86	LINC01596	NR_132380	Upstream	+
chr4_KI270896v1_alt	169375	169473	MACS2_peak_1986	31	4.84953	6.96861	3.11953	78	LINC01596	NR_132380	Upstream	+
chr4_KI270896v1_alt	181540	181693	MACS2_peak_1987	14	3.9678	5.12358	1.48693	88	LINC01596	NR_132380	Upstream	+
chr4_KI270896v1_alt	218916	219033	MACS2_peak_1988	102	7.9356	14.4695	10.21727	43	LINC01596	NR_132380	Upstream	+
chr4_KI270896v1_alt	359196	359309	MACS2_peak_1989	182	7.68443	22.7345	18.23173	65	FRG2	NM_001286820	Downstream	-
chr4_KI270896v1_alt	359557	360295	MACS2_peak_1990	844	19.42452	90.16985	84.41718	455	FRG2	NM_001286820	Downstream	-
chr4_KI270925v1_alt	529985	530172	MACS2_peak_1991	125	8.81734	16.84579	12.51139	80	LINC01596	NR_132380	Upstream	+
chr5	10039	11754	MACS2_peak_1992	285	6.87518	33.33554	28.59089	241	PLEKHG4B	NM_052909	Upstream	+
chr5	118539017	118539227	MACS2_peak_2313	31	4.84953	6.96861	3.11953	187	LOC102467225	NR_104998	Upstream	+
chr5	1192013	1192194	MACS2_peak_1993	131	8.08135	17.54652	13.19057	71	CTD-3080P12.3	NR_109911	Upstream	-
chr5	131436042	131436140	MACS2_peak_2314	40	5.2904	7.94841	4.02217	43	RAPGEF6	NM_001164386	Within	-
chr5	1333559	1333779	MACS2_peak_1994	76	6.80156	11.77445	7.63766	189	CLPTM1L	NM_030782	Within	-
chr5	14347072	14347291	MACS2_peak_2002	154	8.52865	19.88102	15.45512	119	TRIO	NM_007118	Within	+
chr5	1539804	1540159	MACS2_peak_1995	47	5.20544	8.76186	4.78866	209	MIR6075	NR_106723	Upstream	-
chr5	17517693	17517827	MACS2_peak_2003	54	3.65313	9.45031	5.43233	55	LOC102723526	NR_134270	Downstream	+
chr5	17518510	17518695	MACS2_peak_2004	19	2.75154	5.68308	1.98596	177	LOC102723526	NR_134270	Downstream	+
chr5	17526610	17526767	MACS2_peak_2005	97	3.89316	13.94806	9.71943	92	LOC102723526	NR_134270	Downstream	+
chr5	17585096	17585378	MACS2_peak_2006	56	3.80424	9.64273	5.6148	74	LOC102723526	NR_134270	Downstream	+
chr5	17585513	17585635	MACS2_peak_2007	28	3.10374	6.70612	2.89352	59	LOC102723526	NR_134270	Downstream	+
chr5	17597502	17597852	MACS2_peak_2008	48	3.38242	8.83737	4.86008	52	LOC102723526	NR_134270	Downstream	+
chr5	17599137	17599324	MACS2_peak_2009	96	4.63797	13.88392	9.65767	70	LOC102723526	NR_134270	Downstream	+
chr5	176591804	176591939	MACS2_peak_2315	365	14.34253	41.41985	36.5184	65	GPRIN1	NM_052899	Downstream	-
chr5	177961488	177961661	MACS2_peak_2316	113	8.37647	15.64658	11.35266	62	PROP1	NM_006261	Downstream	-
chr5	178585500	178585717	MACS2_peak_2317	249	11.60172	29.57047	24.91009	127	CLK4	NM_020666	Downstream	-
chr5	181045978	181046253	MACS2_peak_2318	263	12.06397	31.05042	26.35858	86	BTNL9	NM_001308245	Within	+
chr5	2145627	2145975	MACS2_peak_1996	207	8.59945	25.33598	20.77058	63	LOC100506858	NR_104616	Downstream	-
chr5	2146446	2146590	MACS2_peak_1997	76	5.29734	11.74759	7.61206	77	LOC100506858	NR_104616	Downstream	-
chr5	2146742	2146911	MACS2_peak_1998	52	4.5754	9.21923	5.21333	56	LOC100506858	NR_104616	Downstream	-
chr5	21496238	21496343	MACS2_peak_2010	52	4.72825	9.20979	5.20412	85	GUSBP1	NR_027028	Within	+
chr5	3323629	3323865	MACS2_peak_1999	129	7.70606	17.34969	12.99997	105	LINC01377	NR_104617	Downstream	+
chr5	3324071	3324230	MACS2_peak_2000	82	6.25939	12.46232	8.29282	47	LINC01377	NR_104617	Downstream	+
chr5	3659973	3660079	MACS2_peak_2001	69	6.37653	11.07596	6.97552	43	IRX1	NM_024337	Downstream	+
chr5	46499116	46499355	MACS2_peak_2011	69	6.613	11.0835	6.97562	29	HCN1	NM_021072	Upstream	-
chr5	46741418	46741525	MACS2_peak_2012	91	7.49474	13.31573	9.10899	39	HCN1	NM_021072	Upstream	-
chr5	47042042	47042162	MACS2_peak_2013	113	8.37647	15.64658	11.35266	46	HCN1	NM_021072	Upstream	-
chr5	47297035	47297158	MACS2_peak_2014	186	11.02167	23.13957	18.62452	57	HCN1	NM_021072	Upstream	-
chr5	47309779	47309889	MACS2_peak_2015	156	9.2081	20.0493	15.61947	26	HCN1	NM_021072	Upstream	-
chr5	47311816	47311926	MACS2_peak_2016	101	7.09459	14.38184	10.13738	45	HCN1	NM_021072	Upstream	-
chr5	47323567	47323665	MACS2_peak_2017	14	3.59712	4.96667	1.41216	31	HCN1	NM_021072	Upstream	-
chr5	47330595	47330716	MACS2_peak_2018	124	7.39339	16.76251	12.43416	69	HCN1	NM_021072	Upstream	-
chr5	47340372	47340480	MACS2_peak_2019	152	8.13906	19.65794	15.23791	32	HCN1	NM_021072	Upstream	-
chr5	47341937	47342068	MACS2_peak_2020	412	14.21722	46.23777	41.23339	61	HCN1	NM_021072	Upstream	-
chr5	47343511	47343609	MACS2_peak_2021	72	5.66829	11.37193	7.25131	41	HCN1	NM_021072	Upstream	-
chr5	47345664	47345762	MACS2_peak_2022	31	4.34162	6.97608	3.12651	36	HCN1	NM_021072	Upstream	-
chr5	47359819	47359917	MACS2_peak_2023	92	6.59258	13.48209	9.26945	48	HCN1	NM_021072	Upstream	-
chr5	47367545	47367685	MACS2_peak_2024	131	8.32127	17.47608	13.12218	69	HCN1	NM_021072	Upstream	-
chr5	47393830	47393931	MACS2_peak_2025	85	7.13746	12.72907	8.5497	46	HCN1	NM_021072	Upstream	-
chr5	47402144	47402269	MACS2_peak_2026	195	10.93117	24.13445	19.59661	43	HCN1	NM_021072	Upstream	-
chr5	47426322	47426431	MACS2_peak_2027	99	7.23589	14.18431	9.94644	65	HCN1	NM_021072	Upstream	-
chr5	47431000	47431110	MACS2_peak_2028	103	7.21402	14.59251	10.33592	62	HCN1	NM_021072	Upstream	-
chr5	47463021	47463130	MACS2_peak_2029	76	6.54501	11.74663	7.6111	21	HCN1	NM_021072	Upstream	-
chr5	47488514	47488694	MACS2_peak_2030	41	4.61148	8.04363	4.11222	97	HCN1	NM_021072	Upstream	-
chr5	47489109	47489262	MACS2_peak_2031	106	6.67223	14.92583	10.659	78	HCN1	NM_021072	Upstream	-
chr5	47500920	47501018	MACS2_peak_2032	63	5.54836	10.40767	6.33576	79	HCN1	NM_021072	Upstream	-
chr5	47507030	47507143	MACS2_peak_2033	131	8.05477	17.49887	13.14435	39	HCN1	NM_021072	Upstream	-
chr5	47515725	47515823	MACS2_peak_2034	82	6.72696	12.45823	8.2889	32	HCN1	NM_021072	Upstream	-
chr5	47531288	47531538	MACS2_peak_2035	45	5.27069	8.5417	4.58137	43	HCN1	NM_021072	Upstream	-
chr5	47554827	47554925	MACS2_peak_2036	75	6.74536	11.68244	7.54949	40	HCN1	NM_021072	Upstream	-
chr5	47562069	47562232	MACS2_peak_2037	89	6.8741	13.12781	8.93391	95	HCN1	NM_021072	Upstream	-
chr5	47565376	47565476	MACS2_peak_2038	63	6.0015	10.45284	6.37859	37	HCN1	NM_021072	Upstream	-
chr5	47604942	47605106	MACS2_peak_2039	109	7.59768	15.26181	10.98381	87	HCN1	NM_021072	Upstream	-
chr5	47612005	47612141	MACS2_peak_2040	37	4.72973	7.63982	3.74573	76	HCN1	NM_021072	Upstream	-
